# High-precision genetic mapping of behavioral traits in the diversity outbred mouse population

**DOI:** 10.1111/gbb.12029

**Published:** 2013-03-20

**Authors:** R W Logan, R F Robledo, J M Recla, V M Philip, J A Bubier, J J Jay, C Harwood, T Wilcox, D M Gatti, C J Bult, G A Churchill, E J Chesler

**Affiliations:** The Jackson LaboratoryBar Harbor, ME, 04609, USA

**Keywords:** Collaborative Cross, complex traits, fine-mapping, heterogeneous stock, mouse populations, QTL mapping

## Abstract

Historically our ability to identify genetic variants underlying complex behavioral traits in mice has been limited by low mapping resolution of conventional mouse crosses. The newly developed Diversity Outbred (DO) population promises to deliver improved resolution that will circumvent costly fine-mapping studies. The DO is derived from the same founder strains as the Collaborative Cross (CC), including three wild-derived strains. Thus the DO provides more allelic diversity and greater potential for discovery compared to crosses involving standard mouse strains. We have characterized 283 male and female DO mice using open-field, light–dark box, tail-suspension and visual-cliff avoidance tests to generate 38 behavioral measures. We identified several quantitative trait loci (QTL) for these traits with support intervals ranging from 1 to 3 Mb in size. These intervals contain relatively few genes (ranging from 5 to 96). For a majority of QTL, using the founder allelic effects together with whole genome sequence data, we could further narrow the positional candidates. Several QTL replicate previously published loci. Novel loci were also identified for anxiety- and activity-related traits. Half of the QTLs are associated with wild-derived alleles, confirming the value to behavioral genetics of added genetic diversity in the DO. In the presence of wild-alleles we sometimes observe behaviors that are qualitatively different from the expected response. Our results demonstrate that high-precision mapping of behavioral traits can be achieved with moderate numbers of DO animals, representing a significant advance in our ability to leverage the mouse as a tool for behavioral genetics

Quantitative trait locus (QTL) mapping is a powerful phenotype-driven approach to identify genetic variants that influence behavioral traits. However, successful identification of causal polymorphisms underlying QTL has been limited (Milner & Buck, 2010), leading some to question the utility of this strategy.

Conventional crosses, widely used for behavioral QTL mapping (Flint 2003), suffer from low mapping resolution and a relative lack of genetic diversity (Yang *et al*. 2007; Roberts *et al*. 2007). Moreover, intense selection for ease of handling is likely to have eliminated many behavioral genetic variants from common laboratory mouse strains. As a result, previous QTL mapping studies have yielded relatively few important findings and have required expensive fine-mapping efforts to resolve the causative loci (Darvasi & Soller 1997).

Advanced intercross lines (AILs) (Darvasi & Soller 1995) and heterogeneous stocks (HS) (Hitzemann *et al*. 2002, Valdar *et al*. 2006) represent strategies that improve mapping resolution. However, the genetic diversity of existing AIL and HS populations is limited due to their derivation from common laboratory strains (Roberts *et al*. 2007). Another strategy exploits existing high diversity and the small ancestral haplotype blocks among common inbred strains by conducting association mapping with strain panels (Pletcher *et al*. 2004; Bennett *et al*. 2010). This strategy has been used successfully in behavioral studies (Park *et al*. 2011; Segall *et al*. 2010). However the complex population history of inbred lines can lead to spurious linkages (Payseur and Place 2007). All of these approaches, including the use of AIL, HS and strain panels, require statistical corrections for population structure that can affect the power of mapping analysis (Kang *et al*. 2008; Cheng *et al*. 2011).

The Collaborative Cross (CC) (Churchill *et al*. 2004; Collaborative Cross Consortium 2012), Diversity Outbred (DO) (Svenson *et al*. 2012) and CC-heterogenous stock (Hitzemann *et al*. 2009) provide alternative mapping populations that encompass a greater level of genetic variation, relatively small haplotype blocks and a uniform population structure that eliminates spurious linkages and provides better power to detect QTL. Early studies with the CC (Aylor *et al*. 2011; Durrant *et al*. 2011; Philip *et al*. 2011) and DO (Svenson *et al*. 2012) demonstrate the wide range of phenotypic diversity and precision of QTL that are obtained using these new resource populations.

The narrow range of behavioral variation in conventional populations has made them sufficiently docile for laboratory tests of mouse behavior. CC mice reveal a greater range of behavioral diversity but, as we have previously demonstrated, this does not make them unsuitable for common behavioral assays (Philip *et al*. 2011). In this study, we characterize behavioral variation in the DO and assess their utility for quantitative genetic analysis using standard behavioral tests. We demonstrate pronounced behavioral variation in the DO, and obtain high-precision QTL mapping results with a moderately sized sample of DO mice.

## Methods

### Subjects

Male and female DO mice (*n =* 283; J:DO, JAX stock number 009376) from generations 4 and 5 (G4 and G5) of outcrossing were obtained from The Jackson Laboratory at 6 weeks of age and transferred to the housing facility via wheeled cart. Mice from the eight inbred founder strains (eight males and eight females per strain) were also obtained from The Jackson Laboratory and were housed and tested under the same conditions as the DO mice.

Mice were housed in duplex polycarbonate cages with a Shepherd Shack on ventilated racks providing 99.997% HEPA filtered air to each cage in a climate-controlled room under a standard 12:12 light–dark cycle (lights on at 0600 h). Pine cob bedding was changed weekly and mice were provided ad-libitum access to food (NIH31 5K52 chow, LabDiet/PMI Nutrition, St. Louis, MO, USA) and acidified water. Initially, all mice were housed in a cage density of five males or females. During the course of the study, ∼20% of G4 and 46% of G5 pens of male mice were separated into smaller groups (1–4) due to aggressive behaviors. All procedures and protocols were approved by The Jackson Laboratory Animal Care and Use Committee, and were conducted in compliance with the National Institutes of Health Guidelines for the Care and Use of Laboratory Animals.

### Genotyping

DNA was prepared from tail biopsies and genotyping was outsourced to GeneSeek (http://www.neogen.com/GeneSeek) for analysis using the Mouse Universal Genotyping Array (MUGA), a 7,851 SNP array built on the Illumina Infinium platform (Collaborative Cross Consortium, 2012). Markers on the MUGA are distributed genome-wide with average spacing of 325 Kb and standard deviation of 191 Kb. The markers uniquely identify any of the CC founders within a window of four to five SNPs. This marker panel provides an average effective sampling sensitivity of just over 1 Mb. Recombination segments smaller than 1 Mb may go undetected.

### General behavioral testing procedures

Mice were subject to a battery of noninvasive behavioral tests to assess activity, anxiety and response to novelty. Tests were arranged by perceived stressfulness in an effort to minimize potential carry-over effects as follows: day 1, open-field; day 3, light–dark box; day 4, visual-cliff avoidance; day 5, hot-plate (data submitted in separate publication) and day 9, tail-suspension test. Mice were randomly assigned to testing groups, such that an equal number of male and female mice were tested each day (*n =* 24 per sex). Mice were between 12–16 weeks of age on the first day of testing. For the open-field, light–dark box and visual-cliff tests, mice were habituated to the testing room for 1 h prior to testing, and 30 min was used for the tail-suspension tests. For each assay, mice were removed by the tail then returned to the clean side of a duplex home-cage until each cage-mate had completed testing. Several experimenters participated in the testing, but a single experimenter handled the mice for each test and the same individuals were in the room during all sessions of a particular test. Behavioral measures were recorded and analyzed by real-time video tracking using Ethovision XT (Noldus Information Technology, Wageningen, The Netherlands).

### Open-field

The open-field arena was an opaque Plexiglass box (39 × 39 × 39 cm) with a dark gray floor, illuminated at 43 ± 4 lux in a 10 × 15 ft room. Zones of the arena were delineated as follows – center, 10 × 10 cm; corners, 4 × 4 cm and periphery, 31 × 4 cm. Each mouse was placed into the center of the arena and allowed to explore for 20 min. The following behaviors were recorded: distance traveled in first 4 min (locomotor activity response to novelty); total distance traveled (general locomotor activity); distance traveled slope over time (habituation); percent time in corners, periphery, and center, and defecation (anxiety-like behaviors); and time in corners, periphery and center slopes (habituation and anxiety-like behaviors).

### Light–dark box

The light–dark box consisted of an insert evenly dividing the open-field apparatus into light–dark compartments, with the light compartment illuminated at 17 ± 2 lux. The compartments were separated with a sliding door that is closed during placement of mice into the chamber. Mice were placed into the dark compartment and a 20-min recording began when the lid was closed (Henderson *et al*. 2004). The following behaviors were measured: distance traveled in the light (habituation and anxiety-like behaviors); number of light–dark transitions, percent duration spent in light and defecations (anxiety-like behaviors); and percent duration in light first 4 min and time in light slope over time (habituation).

### Visual-cliff

A visual-cliff avoidance test was conducted in open-field boxes with clear Plexiglas bottoms that were secured, so half of the floor overhung the table-top to create an appearance of a ledge drop-off. A checkerboard tablecloth draped from table-top to floor served to enhance the visual appearance of the cliff. The vertical distance between the box floor and the testing room floor was 93 cm. An opaque neutral zone (10 × 10 cm) was located in the center of the box floor. The entire field of view was covered with black and white checkerboard to emphasize changes in depth. To initiate a trial, mice were placed onto the neutral center region and allowed to freely explore for 20 min. The following behaviors were recorded: total distance traveled (general locomotor activity); distance traveled in the top and bottom during the first 4 min (response to novelty) and entire session; number of entries into top and bottom; number of top–bottom transitions; percent duration in neutral, top and bottom portions of the arena (avoidance and anxiety-like behaviors); slopes of time and distance in top and bottom (habituation); mean velocity in top and bottom; and duration spent immobile in top and bottom of the arena. The test was performed in an effort to recapitulate elevated plus maze anxiety while ensuring that wild-derived mice would remain in an enclosed apparatus with minimal interference from the investigator during trials.

### Tail-suspension test

The test consisted of two consecutive days with each trial lasting 5 min. A paper cone was placed on the tail to limit the mice from climbing during the testing session. Using masking tape, individual mice were suspended by a point near the tip of the tail on a horizontal ring-stand bar elevated ∼30 cm above the floor of the apparatus. Several behaviors were measured: latency to first immobility and duration of immobility (depression-like behaviors); and frequency of climbing behavior, which is not typically studied as a depression-related measure but which is an interesting wildness-related behavior.

Behavioral measures in the progenitor strains were compared using two-way anova to estimate main effects of strain and sex, and strain × sex interactions. Heritability estimates were calculated as the percent of variance attributed to strain using the restricted maximum likelihood (REML) variance components with strain as a random effect (JMP 9, SAS Institute Inc., Cary, NC, USA).

### QTL mapping in the DO and phenotypic analyses in the DO progenitors

QTL mapping was carried out as described by Svenson *et al*. (2012). Founder haplotypes were reconstructed using a Hidden Markov Model (HMM) that produced a matrix of 36 genotype probabilities for each sample at each SNP. Genotype probabilities at each SNP were then collapsed to an eight-state allele dosage matrix by summing the probabilities contributed by each founder. Each behavioral phenotype was assessed for normality and logarithmic or square-root transformations were applied as needed to achieve approximate normality. Mapping was performed using QTLRel software (http://www.palmerlab.org/software) (Cheng *et al*. 2011). A mixed model was fit with sex and experimental group as additive covariates and a random effect was included to account for kinship. Regression coefficients for additive effects of founder alleles were estimated at each genomic location. Regions with shared haplotypes were identified using the Mouse Phylogeny Viewer (http://www.msub.csbio.unc.edu) and allelic effects were compared to all known genomic variants from the Wellcome Trust Sanger mouse genomes project (http://www.sanger.ac.uk) (Keane *et al*. 2011; Yalcin *et al*. 2011; Yang *et al*. 2011) to identify positional candidates (Churchill *et al*. 2012). Significance thresholds were obtained by performing 1000 permutations of the genome scans with phenotype data being shuffled among individuals and 1.5 LOD support intervals from the linear model were determined for significant (*P* < 0.05) and suggestive (*P* < 0.10) QTL peaks.

Each of the different behavioral assays shares an underlying relation to constructs of activity, anxiety, stress response and other traits. To directly assess genetic regulators of derived constructs, we performed a principal components analysis of behavioral measures from each of the tests, excluding those that were arithmetically derived from one another, and individual time points from sets of repeated measures. Traits with extremely low genetic variance (<10% of total variance) in the founder strains were also excluded from the analysis. The factor scores derived from this analysis were used for QTL mapping.

To directly assess the possible influence of locomotor activity in wild-derived mice on behavioral variation and QTL detection, we performed a separate mapping analysis for each wild-derived QTL using locomotor activity measurements from their respective testing apparatus as a covariate. For percent time light in the light–dark box, total distance traveled in the open-field was used a covariate because activity in the dark side, and thus total activity in the light–dark box, could not be measured using our equipment.

### Pharmacological validation of anxiety-like behavior

The light–dark box test is intended to measure anxiety-like behavior and has been pharmacologically validated using several anxiolytic drugs (Bourin & Hascoët, 2003). An independent cohort of DO mice (*n =* 16 per sex) was tested in the light–dark box following an *i.p*. injection of either saline or diazepam (4 mg/kg) on two separate days. A crossover design was used, such that on day 1, male (*n =* 8) and female (*n* = 8) mice received either saline or diazepam, followed by the opposite treatment on day 2. Mice were placed in the light–dark box ∼30 min postinjection, and percent time in light was measured over 20 min.

## Results

### Heritability of behavioral measures

Heritability estimates for the 38 behavioral measurements were calculated from the progenitor strain data using variance components from a mixed model with strain as a random effect. In general, heritability estimates were consistent with other studies (Brown *et al*. 2012; Koide *et al*. 2000; Mhyre *et al*. 2005; Miller *et al*. 2010; Philip *et al*. 2010, 2011; Wahlsten *et al*. 2006). A majority of the traits (29 of 38) had heritability estimates ≥20% (Table 1). General locomotor activity was highly heritable across each of the arena-based tests (open-field, 82%; light–dark box, 68%; and visual-cliff, 80%). Anxiety- and depression-related traits showed moderate heritability (percent time in center in the open-field, 15%; percent time in light in the light–dark box, 24%; and on the tail suspension test, duration immobile, 7% and frequency of immobility, 22%). Heritability estimates for slope of behavioral measures over time intervals ranged from low (time in periphery of open-field, 5%) to high (distance traveled in suspended half or ‘bottom’ of visual-cliff, 74%), indicating that habituation-related behaviors are strain dependent. Of the traits for which significant loci were mapped, the heritability estimates were lowest for center time slope (28%) and highest for duration of immobility (91%) in the open-field. Heritability analysis confirmed that most of the traits in this study were subject to substantial genetic influence (Table 1).

**Table 1:** Heritability estimates of behaviors subject to QTL mapping

Traits	% Heritability
*Open field*	
Distance (cm) traveled in first 4 min	82.27
Total distance (cm) traveled	85.36
Distance traveled slope	9.30
% time in corners	28.11
Time in corners slope	22.36
% time in periphery	35.92
Time in periphery slope	5.28
% time in center	15.45
Time in center slope*	25.30
% time immobile*	91.50
*Light–dark box*	
Distance (cm) traveled in light	68.45
Number of light–dark transitions	28.89
% time in light†	24.04
% time in light first 4 min	2.66
Time in light slope*	25.19
*Visual-cliff avoidance arena*	
Total distance (cm) traveled	80.25
Total duration (s) immobile	87.97
Total transitions between top and bottom	72.48
Distance (cm) traveled in top	68.06
Distance (cm) traveled in top first 4 min	51.42
Entries into top	47.70
Duration (s) immobile in top	68.87
Mean velocity in top	64.17
Distance (cm) traveled in bottom	72.11
Distance (cm) traveled in bottom first 4 min	50.75
% time in bottom	51.32
% time in top	48.62
% time in neutral	5.23
Entries into bottom	62.70
Duration (s) immobile in bottom	19.60
Mean velocity in bottom	16.08
Distance (cm) traveled in bottom slope	73.56
Time in bottom slope	69.31
Distance in bottom to total arena (ratio)†	47.98
*Tail suspension test*	
Climbing frequency*	85.08
Duration (s) immobile	7.50
Frequency of immobility	22.26
Latency to first immobile	0.78

* Sig. QTL *P <* 0.05.

† Suggestive QTL *P <* 0.10.

### Phenotypic analyses in the progenitor strains of the DO mouse population

Generalized linear models were used to test main effects of strain and sex, and strain × sex interactions for each behavior in the eight progenitor strains. Behaviors measured in the open-field, light–dark box, visual-cliff and tail-suspension test were all influenced by strain. Effects due to sex and strain × sex interactions (Tables 2 and S1–S4) were observed for some traits.

**Table 2:** Summary of phenotypes for QTL in progenitor and DO mice

			Open-field				Light–dark box				Tail-suspension		Visual-cliff	
						
			Time in center slope		% time immobile		% time in light		Time in light slope		Climbing frequency		Distance bottom (ratio)	
								
Strain	Sex	N	Mean ± SEM	Min–Max	Mean ± SEM	Min–Max	Mean ± SEM	Min–Max	Mean ± SEM	Min–Max	Mean ± SEM	Min–Max	Mean ± SEM	Min–Max
Diversity Outbred	♀	144	−0.35 ± 0.06	−2.07–2.12	81.31 ± 0.96	38.09–99.22	36.85 ± 1.35	0.04–84.17	−0.08 ± 0.05	−2.37–1.74	1.58 ± 0.5	0–59	0.37 ± 0.01	0.01–0.66
	♂	139	−0.43 ± 0.05	−1.87–0.69	85.97 ± 0.78	52.49–99.4	38.63 ± 1.44	1.21–67.3	−0.13 ± 0.05	−2.4–1.84	0.56 ± 0.22	0–25	0.37 ± 0.01	0.01–0.63
129S1/SvImJ	♀	8	−0.44 ± 0.24	−1.35–0.39	79.25 ± 11.08	59.32–97.55	5.37 ± 2.17	0.02–12.44	−1.41 ± 0.23	−2.18 to –0.66	0 ± 0	0–0	0.17 ± 0.02	0.1–0.29
	♂	8	−0.63 ± 0.31	−1.96–0.59	82.4 ± 5.84	72.69–90.77	22.82 ± 9.12	0.64–52.04	−0.98 ± 0.39	−2.41–0.15	0 ± 0	0–0	0.23 ± 0.07	0.07–0.52
A/J	♀	8	−0.93 ± 0.36	−2.74–0	96.57 ± 1.83	93.77–99.11	3.76 ± 2.09	0.13–16.79	−0.93 ± 0.37	−1.94–0.86	0 ± 0	0–0	0.22 ± 0.04	0.1–0.26
	♂	8	−1.02 ± 0.27	−2.4–0	97.05 ± 0.92	95.23–98.12	6.08 ± 3.92	0.21–24.91	−1.63 ± 0.33	−2.89 to –0.54	0 ± 0	0–0	0.25 ± 0.09	0.07–0.58
C57BL/6 J	♀	8	0.21 ± 0.10	−0.33–0.58	55.91 ± 5.94	48.95–62.91	46.06 ± 3.23	28.14–57.70	−0.03 ± 0.03	−0.14–0.14	0 ± 0	0–0	0.45 ± 0.01	0.38–0.5
	♂	8	0.11 ± 0.09	−0.29–0.57	54.7 ± 6.72	45.91–65.55	61.13 ± 7.83	24.82–97.10	0.10 ± 0.04	−0.01–0.32	0.13 ± 0.13	0–1	0.42 ± 0.02	0.31–0.51
Cast/EiJ	♀	8	0.08 ± 0.09	−0.38–0.43	31.76 ± 4.73	27.57–38.24	53.18 ± 2.94	45.98–71.72	0.03 ± 0.04	−0.11–0.25	6 ± 0.53	4–8	0.47 ± 0.02	0.37–0.53
	♂	8	0.10 ± 0.09	−0.15–0.58	41.02 ± 8.54	25.58–51.07	35.75 ± 9.01	10.69–52.56	0.26 ± 0.20	−0.02–0.85	2.25 ± 1.65	0–7	0.44 ± 0.04	0.3–0.61
NOD/ShiLtJ	♀	8	−0.09 ± 0.06	−0.31–0.08	42.44 ± 7.31	32.83–53.59	54.13 ± 4.92	37.19–75.35	−0.03 ± 0.03	−0.19–0.05	0 ± 0	0–0	0.41 ± 0.02	0.33–0.51
	♂	8	0.07 ± 0.11	−0.25–0.77	44.69 ± 5.6	36.61–55.07	61.03 ± 5.87	40.96–95.52	−0.01 ± 0.01	−0.05–0.06	0 ± 0	0–0	0.43 ± 0.03	0.26–0.55
NZO/H1LTJ	♀	8	0.17 ± 0.33	−1.57–1.25	76.73 ± 9.88	62.63–86.91	26.24 ± 6.84	4.35–57.54	−0.43 ± 0.21	−1.73–0.18	0 ± 0	0–0	0.4 ± 0.04	0.29–0.56
	♂	8	−0.21 ± 0.2	−0.92–0.79	85.15 ± 5.13	77.64–91.3	76.22 ± 8.2	45.96–98.17	0.03 ± 0.11	−0.58–0.54	0 ± 0	0–0	0.39 ± 0.04	0.22–0.55
PWK/PhJ	♀	8	0.08 ± 0.20	−0.83–1.04	50.00 ± 10.21	37.38–63.29	42.07 ± 6.41	20.31–67.37	0.12 ± 0.22	−0.34–1.07	13 ± 1.79	5–20	0.41 ± 0.04	0.22–0.54
	♂	8	−0.13 ± 0.17	−0.94–0.67	52.86 ± 5.92	40.84–58.98	55.77 ± 11.67	24.12–90.61	−0.58 ± 0.44	−2.94–0.14	11 ± 0.72	8–14	0.44 ± 0.04	0.29–0.6
WSB/EiJ	♀	8	−0.19 ± 0.12	−0.88–0.19	44.28 ± 9.27	34.96–61.66	64.54 ± 8.55	37.97–93.9	0.11 ± 0.03	0–0.24	14.75 ± 1.5	9–23	0.41 ± 0.02	0.34–0.47
	♂	8	−0.18 ± 0.15	−1.09–0.12	39.63 ± 2.51	35.77–42.9	46.89 ± 3.13	35.74–59.87	0.04 ± 0.08	−0.24–0.37	4.38 ± 1.4	0–11	0.4 ± 0.03	0.3–0.51

For the open-field test, we observed a significant effect of strain on center time slope (F_(1,7)_ = 7.14, *P* < 0.0001). Strains 129S1/SvlmJ and A/J spent less time in the center of the open-field over the testing session (negative slope) and C57BL6/J mice spent the most time in the center (positive slope; Table 2). There were main effects of strain (F1_(1,7)_ = 163.26, *P* < 0.0001) and sex (F_(1,7)_ = 4.35, *P* = 0.04) for percent time immobile. The wild-derived strains, PWK/PhJ, WSB/EiJ and CAST/EiJ, were among the most mobile of the progenitor strains (Table 2). NZO/H1LtJ, 129S1/SvlmJ and A/J were the least mobile strains (Table 2). Females of all strains exhibited greater duration of immobility in the open-field than their male counterparts (Table 2).

For the light–dark box, we observed a significant effect due to strain (F_(1,7)_ = 13.56, *P* < 0.0001) and a strain × sex interaction (F_(1,7)_ = 2.17, *P* = 0.04) for change (slope) in time spent in the light side. Strains 129S1/SvlmJ, A/J, and PWK/PhJ spent the least time in the light side (negative slope), while WSB/EiJ and C57BL6/J spent the most (Table 2). Females of strains 129S1/SvlmJ and A/J spent less time in the light compared to males, whereas the male CAST/EiJ and PWK/PhJ mice spent less time in the light compared to their female counterparts (Table 2). We observed significant main effects of strain (F_(1,7)_ = 18.8431, *P* < 0.0001) and sex (F_(1,7)_ = 7.13, *P* = 0.0048), and an interaction (F_(1,7)_ = 5.55, *P* < 0.0001) for percent time in the light side of the light–dark box. The 129S1/SvlmJ and A/J strains spent the least amount of time in the light side, with the males of these two strains showing more time in the light than the females (Table S2). In contrast, CAST/EiJ females spent more time in the light compared to males.

During the tail-suspension test, climbing frequency varied widely among progenitor strains. We observed significant main effects of strain (F_(1,7)_ = 86.42, *P* < 0.0001) and sex (F_(1,7)_ = 27.98, *P* < 0.0001), and a strain × sex interaction (F_(1,7)_ = 12.57, *P* < 0.0001). Only the three wild-derived strains, CAST/EiJ, WSB/EiJ and PWK/PhJ, displayed climbing behavior during the tail-suspension test. PWK/PhJ mice climbed most frequently, followed by the WSB/EiJ and CAST/EiJ mice. The CAST/EiJ and WSB/EiJ females climbed more than their male counterparts.

In the visual cliff avoidance arena, there was a main effect of strain (F _(1,7)_ = 13.40, *P* < 0.0001) for locomotor activity in the bottom area of the arena. The CAST/EiJ strain spent the greatest amount of time in the bottom of the arena, followed by the 129S1/SvlmJ and A/J strains. No other sex effects or interactions were detected.

### Phenotypic variation in the DO population

We expected phenotypic variation in the DO to expand beyond the range of the parental strains due to heterozygosity. Our sample of 283 DO mice recapitulated the range of variation observed in the eight progenitor strains for most traits (Fig. 5). DO phenotype values spanned the entire range of the progenitors for center time slope and percent time immobile in the open-field (Fig. 2a,b), percent time in the light and light time slope in the light–dark box (Fig. 3a,b), distance traveled in the bottom of the visual cliff (Fig. 4a), and climbing frequency during the tail-suspension test (Fig. 5a).

**Figure 1:**
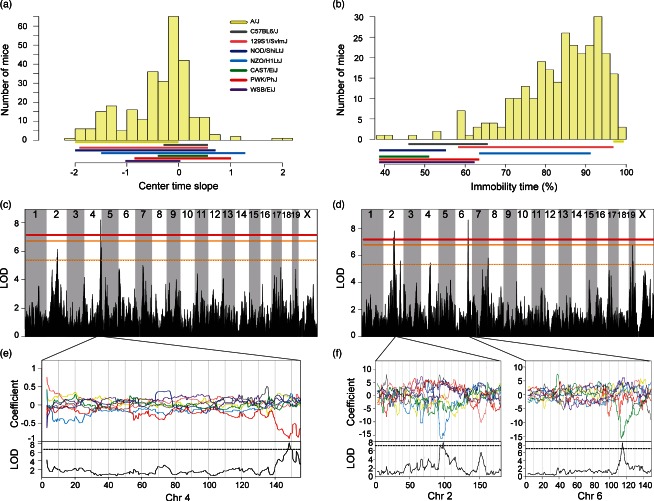
Significant genome-wide QTL for behaviors in the open-field arena Phenotypic distributions of DO mice for (a) center time slope and (b) percent time immobile. Solid colored bar below histograms represent phenotype ranges (min–max) of each progenitor strain. Significant genome-wide QTL for (c) center time slope and (d) percent time immobile. Horizontal lines represent permuted significance thresholds as follows, solid red line (significant, *P* < 0.05), solid (highly suggestive, *P* < 0.10) and dashed orange lines (suggestive, *P* < 0.63). Allelic effect plots of eight coefficients of the QTL mixed model representing the effect of each founder haplotype on phenotype. (e) The PWK/PhJ allele on chromosome 4 was associated with less time spent in the center of the open-field over the testing session. (f) The NZO/H1LtJ and CAST/EiJ alleles on Chrs 2 and 6 respectively, were associated with decreased mobility in the open-field. Dashed line is the maximum LOD −1.5, defining the 95% support interval of the QTL.

**Figure 2:**
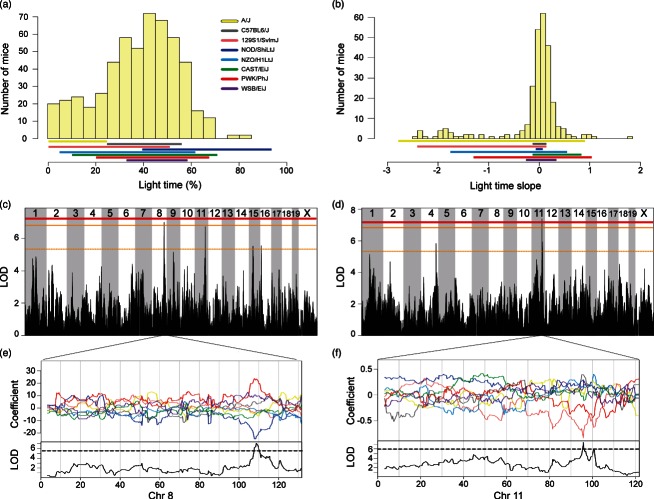
Significant genome-wide QTL for behaviors in the light–dark box Phenotypic distributions of DO mice for (a) percent time spent in the light and (b) time spent in the light slope. Solid colored bar below histograms represent phenotype ranges (min–max) of each progenitor strain. Significant genome-wide QTL for (c) percent time spent in the light and (d) time spent in the light slope. Horizontal lines represent permuted significance thresholds as follows, solid red line (significant, *P* < 0.05), solid (highly suggestive, *P* < 0.10) and dashed orange lines (suggestive, *P* < 0.63). Allelic effect plots of eight coefficients of the QTL mixed model representing the effect of each founder haplotype on phenotype. (e) An increasor PWK/PhJ allele and a decreasor NOD/ShiLtJ on Chromosome 8 was associated with time spent in the light. (f) The 129S1/SvlmJ allele on Chromosome 11 was associated with a decreased amount of time spent in the light side over the testing session. Dashed line is the maximum LOD −1.5, defining the 95% support interval of the QTL.

**Figure 3:**
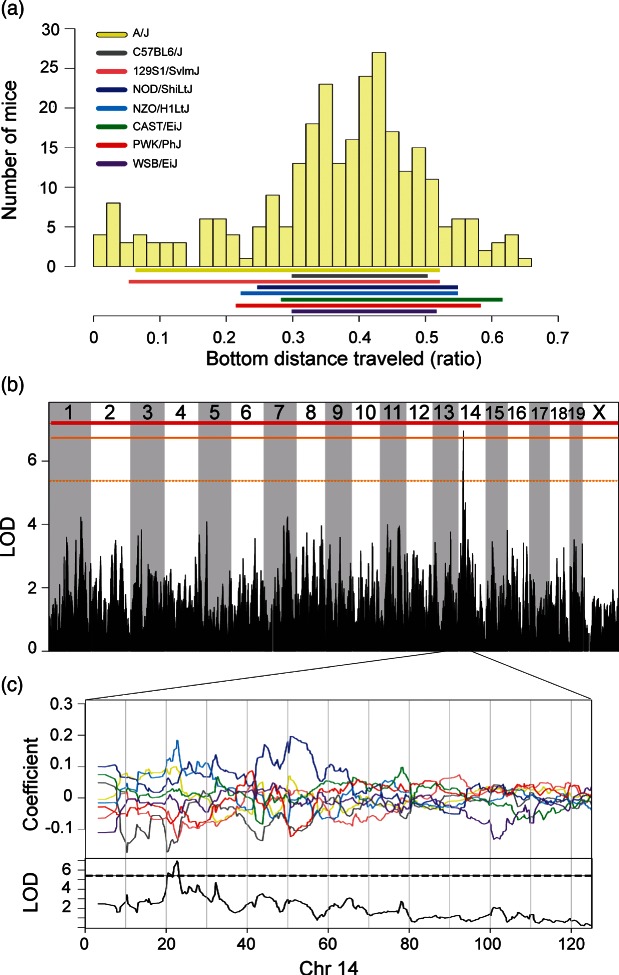
Significant genome-wide QTL for behaviors in the visual-cliff avoidance test Phenotypic distribution of DO mice for (a) ratio of distance traveled in the bottom area. Solid colored bar below histograms represent phenotype ranges (min–max) of each progenitor strain. Significant genome-wide QTL for (b) bottom distance traveled. Horizontal lines represent permuted significance thresholds as follows, solid red line (significant, *P* < 0.05), solid (highly suggestive, *P* < 0.10) and dashed orange lines (suggestive, *P* < 0.63). Allelic effect plots of eight coefficients of the QTL mixed model representing the effect of each founder haplotype on phenotype. (c) An increasor NZO/H1LtJ allele and a decreasor 129S1/SvlmJ allele on Chromosome 14 were associated with distance traveled in the bottom of the visual-cliff. Dashed line is the maximum LOD −1.5, defining the 95% support interval of the QTL.

**Figure 4:**
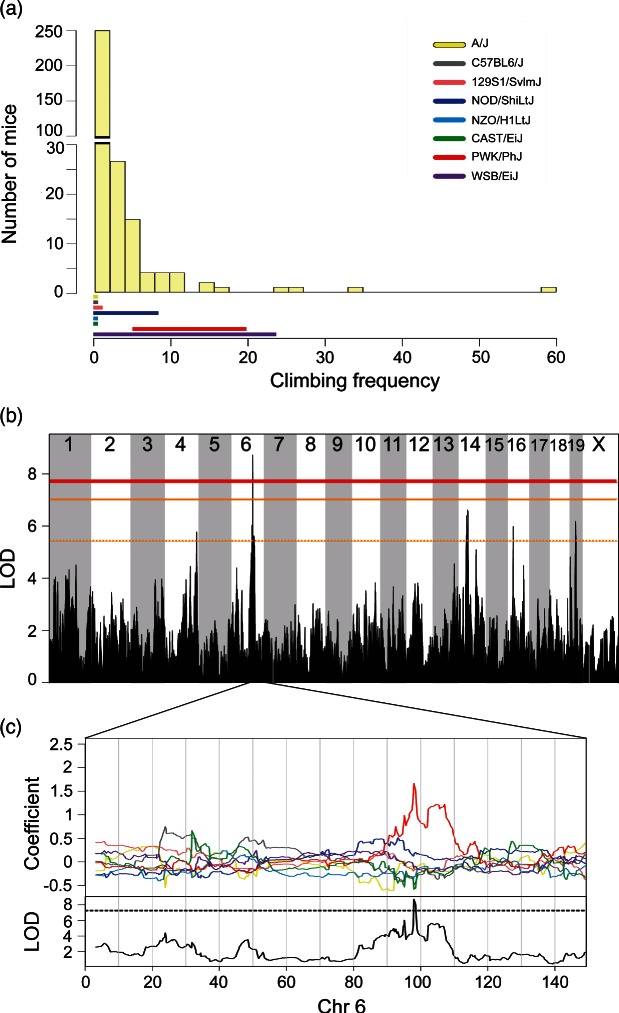
Significant genome-wide QTL for behaviors during the tail-suspension test Phenotypic distribution of DO mice for (a) climbing behavior. Solid colored bar below histograms represent phenotype ranges (min–max) of each progenitor strain. Note that this trait was log transformed before mapping to satisfy model assumptions. Significant genome-wide QTL of (b) frequency of climbing. Horizontal lines represent permuted significance thresholds as follows, solid red line (significant, *P* < 0.05), solid (highly suggestive, *P* < 0.10) and dashed orange lines (suggestive, *P* < 0.63). Allelic effect plots of eight coefficients of the QTL mixed model representing the effect of each founder haplotype on phenotype. (c) The PWK/PhJ allele on Chromosome 6 was associated with increased frequency of climbing. Dashed line is the maximum LOD −1.5, defining the 95% support interval of the QTL.

**Figure 5:**
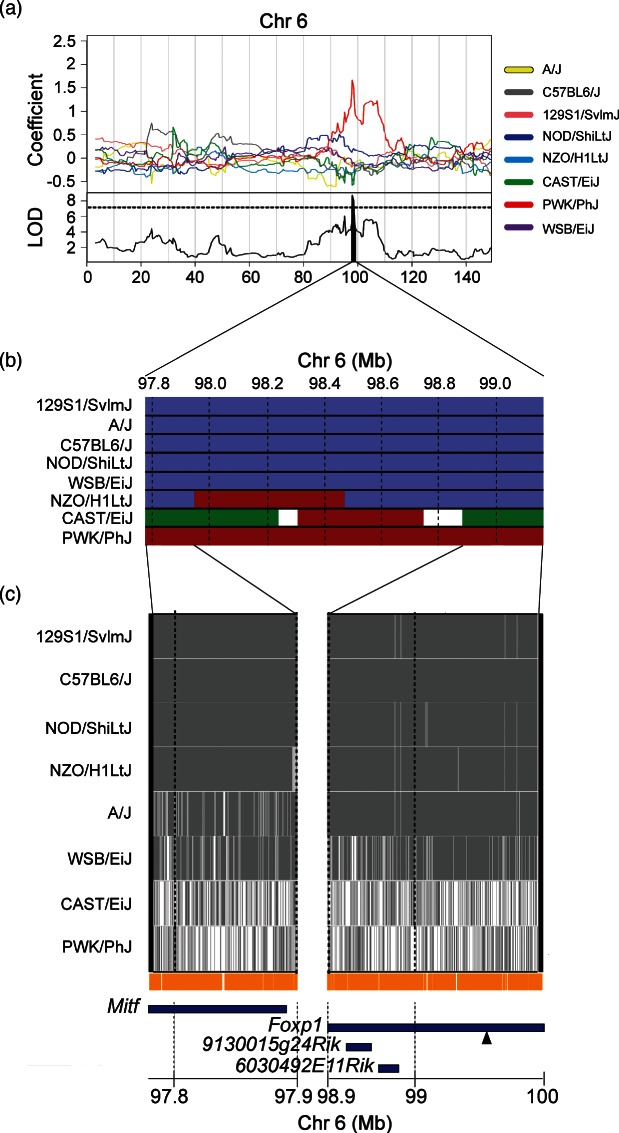
Narrowing QTL support interval using phylogeny and sequencing information based on allele effect estimates (a) Allelic effect plot displaying PWK/PhJ allele association with increased climbing frequency during tail-suspension test. (b) Comparison of IBD regions between eight founder strains reveals two regions where PWK/PhJ haplotypes are different from the remaining seven founder strains. Solid lines extending below haplotype plot anchor boundaries of these regions. (c) SNP distribution plots across two regions of polymorphisms segregating only in the PWK/PhJ strain. Numerous private PWK/PhJ polymorphisms are present in these regions (vertical orange bars). Positional candidates (blue bars) in the narrowed interval are displayed below. The single Sanger SNP matching the founder effect pattern is marked with a triangle.

### QTL mapping of behavioral phenotypes in the DO population

#### Open-field arena

We mapped two large-effect QTL for open field measures. A single QTL (12.8% VAF) for center time slope was detected on Chromosome 4. This QTL has a 1.61 Mb (147.68–149.29) support interval (Fig. 1c), containing 32 genes (Tables 3 and S5). The PWK/PhJ allele is associated with decreased time spent in the center of the open-field arena (Fig. 1e), which is consistent with less time spent in the center of the open field by the PWK/PhJ progenitor strain. Numerous SNPs unique to PWK/PhJ are located in 3′ and 5′ UTR, intronic, and intergenic regions of genes within the interval. This, together with the lack of informative recombination breakpoints in the DO, precluded further narrowing of the candidates in the interval. Additional QTL for duration immobile in the open-field were detected on chromosomes 2 and 6 (11.4% and 12.5% VAF, respectively). The Chr 2 support interval spans 7 Mb (93.2–100.21) (Fig. 1d), and despite being the largest support interval found in this study, contains only 35 genes (Tables 3 and S6). The allele effect plots indicate that NZO/H1LtJ alleles on chromosome 2 are associated with increased immobility in the open-field (Fig. 1f). Among the progenitors, NZO/H1LtJ was among the least mobile strains in the open-field (*P* < 0.0001). Based on haplotype analyses, the larger 7 Mb interval on Chromosome 2 was parsed into three smaller regions (93.89–94.13, 96.13–96.46 and 97.85–98.02 Mb). The first region (240 kb) contains a nonsynonymous coding SNP in *Hsd17b12* that is unique to the NZO/H1LtJ strain. The QTL on Chr 6 has a support interval of 1.87 Mb (114.07–115.94) (Fig. 1d) containing 15 genes (Table 3 and S6). CAST/EiJ alleles on chromosome 6 are associated with decreased immobility in the open-field (Fig. 1f). CAST/EiJ mice were the most mobile of progenitor strains (*P* < 0.0001), resembling the pattern of allele effect estimates at the QTL. Based on haplotype comparisons between the CAST/EiJ and all other strains, the QTL support interval on Chromosome 6 was parsed into two separate regions (114.07–114.39 and 115.03–115.93 Mb). In the first interval, there are several SNPs unique to the CAST/EiJ strain, including two adjacent non-synonymous coding SNPs in the *Slc6a1* gene, along with several 3′ UTR SNPs in the *Hrh1* gene. The second region was rich in CAST/EiJ SNPs, with the following SNPs found in the 5′ and 3′ UTR of the following genes: *Syn2*, *Pparg*, *Mkrn2*, *Cand2*, *Rpl32*, *Mbd4* and *Rho*. Nonsynonymous coding SNPs were found in *Tsen2*, *Raf1*, *Tmem40*, *Mbd4*, *Ift122*, *H1foo* and *Plxnd1*. A few of these genes have been implicated in neurobehavioral phenomena, including the GABA transporter 1 gene *Slc6a1*, which is a candidate for anxiety-related disorders (Thoeringer *et al*. 2009), and *Syn2*, which has been previously implicated in schizophrenia (Dyck *et al*. 2009, 2011).

**Table 3:** Summary of behavior QTL in DO mice

						Positional Candidates				
							
Traits	Chr	Peak Marker	LOD	1.5 LOD CI (Mb)	Interval Width (Mb)	Protein Coding	Pseudo- gene	miRNA	Total	% VAF QTL
Time in center slope in the open-field	4	backupUNC040363260	8.20	147.68–149.29	1.61	25	7	0	32	12.81
Immobility in	2	backupUNC021331957	7.82	93.2–100.21	7	12	21	2	35	11.43
open-field	6	JAX00145678	8.63	114.07–115.94	1.87	12	3	0	15	12.53
	19*	UNC190040392	6.80	34.53–36.27	1.74	14	5	2	21	
% Time spent in light side of light–dark box*	8	JAX00679421	7.14	107.54–110.44	2.89	82	11	3	96	10.83
Time in light slope in the light–dark box	11	UNC110549757	7.40	95.01–96.55	1.53	33	5	3	41	11.1
Climbing frequency during tail-suspension test	6	UNC060396166	8.69	97.77–98.9	1.12	3	2	0	5	13.74
Distance traveled (ratio) in bottom area of visual-cliff*	14	UNC140101805	6.95	21.55–23.18	1.63	13	1	0	14	10.89

* Suggestive QTL *P <* 0.10.

#### Light–dark box

A significant QTL for duration in the light side (slope) was mapped to Chr 11 (11.1% VAF) with a 1.53 Mb (95.01–96.55) (Fig. 2d) support interval containing 41 genes (Table 3 and S7). The 129S1/SvlmJ allele is associated with a pronounced decrease in time spent in the light (Fig. 2f). SNPs unique to 129S1/SvlmJ are present in the 3′ and 5′ UTR, intronic and intergenic regions of *Zfp652* and *Skap1*, and a synonymous coding SNP was found in *Calcoco2*. The 129S1/SvlmJ progenitor strain spent progressively less time in the light side (negative slope) over the testing session (*P* < 0.0001; Table 2). A highly suggestive QTL for the percentage of time spent in the light was detected on Chr 8 (10.83% VAF), with a 2.89 Mb (107.54–110.44) support interval (Fig. 2c) containing 96 genes (Table 3 and S8). An increasing effect is associated with PWK/PhJ alleles and a decreasing effect is associated NOD/ShiLtJ alleles (Fig. 2f), which is consistent with the observation that PWK/PhJ progenitors spent more time in the light compared to other strains. However, the NOD/ShiLtJ were also among the highest strains for this trait (*P* = 0.04; Table 2). Interestingly, there are non-synonymous coding SNPs in genes that segregate in either the NOD/ShiLtJ strain (*Cdh1*,*Terf2*), or the PWK/PhJ strain (*Ces2h*, *Ces4a*, *Exoc3l*, *E2f4*, *Elmo3*, *Fhod1*, *Plekhg4*, *Kctd19*, *Hsd11b2*, *Ritpr*, *Acd*, *Pard6a*, *Ranbp10*, *Cenpt*, *Nm1l*, *Psmb10*, *Ddx28*, *Dus2l*, *Nfatc3*, *Pla2g15*, *Slc7a6*, *Prmt7*, *Zfp90*, *Cdh3*, *Tmed6*, *Nfat5*), but not both. There are several intronic polymorphisms private to both PWK/PhJ and NOD/ShiLtJ in the *Cdh1* gene. *Cdh1* has been implicated in neuronal function, including axonal growth (Konishi *et al*. 2004) and long-term potentiation in the hippocampus (Fonseca *et al*. 2006), as well as hippocampal-dependent behaviors, such as contextual fear conditioning (Kim *et al*. 1992; Li *et al*. 2008).

Anxiety-like behavior in the light–dark box has been historically validated by sensitivity to known anxiolytics, such as benzodiazepines. To determine whether light–dark box behavior in DO mice is responsive to diazepam, mice were injected with the drug 30-min prior to light–dark box testing. A moderate dose of diazepam (4 mg/kg) significantly increased the time mice spent in the light side compared to their respective saline trial (33.4 vs. 26.68%; *P* < 0.019). There were no significant treatment order or sex effects. Of the 32 DO mice, eight of the mice never left the dark compartment after diazepam injection. On their saline trial, light–dark behavior for these mice was similar to that of mice that entered the light side following diazepam suggesting that they were not extremely anxious but rather sedated by the drug. The order of diazepam vs. saline administration did not appear to influence this behavior. Saline treated mice displayed a negative percent time slope, indicating a slight decrease in time spent in light over the testing session, whereas mice on diazepam trial showed an increase in time (slope difference of 0.53, *P* < 0.0097).

#### Visual-cliff avoidance test

Among 18 measured traits for the visual-cliff test, only a single suggestive QTL for distance traveled in the bottom of the arena was detected. This mapped to chromosome 14 (10.89% VAF) (Fig. 3b) with a 1.63 Mb (21.55–23.18) support interval containing 14 genes (Tables 3 and S9). The 129S1/SvlmJ and NOD/ShiLtJ alleles were associated with low and high distance traveled in the bottom, respectively (Fig. 3c). The other founder alleles were associated with moderate trait values. Similarly, the 129S1/SvlmJ and NOD/ShiLtJ progenitors were among the lowest and highest strains for distance traveled in the bottom (*P <* 0.0001; Table 2). There are 55 non-synonymous coding SNPs in this region, of which two are private to either the 129S1/SvlmJ and NOD/ShiLtJ strains (Fig. 3c) and lie within *Myst4* (Kraft *et al*. 2011), a gene involved in transcription and histone acetylation. Additional polymorphisms consistent with this pattern are located in the 3′ UTR of *Myst4*, *Comtd1* and *Zfp503*.

#### Tail-suspension test

A significant QTL for climbing behavior was detected on chromosome 6 (Fig. 4b) with a 1.40 Mb (97.77–99.17) support interval containing only three protein-coding genes and two pseudogenes (Tables 3 and S10). PWK/PhJ alleles are associated with an increase in climbing frequency (Fig. 4c). The PWK/PhJ strain climbed more frequently than all of the other strains (*P* < 0.0001; Table 2). Within the QTL interval, there were two regions in the PWK/PhJ haplotype that were not shared with other strains (97.77–97.94 and 98.79–99.17) (Fig. 5b). Within the latter region, there were two non-synonymous coding SNPS, one of which is consistent with the allelic effects in the PWK/PhJ (Fig. 6c). The SNP is located in the *Foxp1* gene (Fig. 5c). Unlike time spent immobile, climbing behavior is not interpreted as a depression model. For the conventional immobility phenotype, a suggestive locus was identified on Chr 7 (data not shown).

### High-precision QTL intervals in the DO population for complex behavioral traits

We performed QTL analysis on 38 traits from 4 behavioral assays and identified 5 significant (*P <* 0.05) and 3 suggestive (*P <* 0.10) QTL (Table 3). The median support intervals for the significant and highly suggestive QTL were 1.61 and 1.74 Mb, respectively. The largest interval, 7 Mb, contained 34 genes and the smallest, 1.12 Mb, contained only three genes (Table 3, S5-10). Thus we have demonstrated that QTL mapping of behavioral traits using the DO mouse population can provide precise QTL support intervals containing small numbers of genes. Genes can be prioritized and further investigated using known genomic variants that match allele effects within the support interval.

### Assessing the influence of activity on behavior in the DO

Behavioral testing procedures in mice have largely been developed for applications to common laboratory strains. The introduction of wild-derived alleles, as in the DO mice, raises concerns that increased locomotor activity associated with these alleles may invalidate testing results. We found that measures of total activity in each apparatus are correlated but that anxiety and habituation measures were not correlated with activity within or across tests (Table S11).

Principal component mapping can be used to map global mediators of related behaviors, and to isolate independent factors of behavioral variation that may be influencing the outcomes of correlated measures of behavior. In particular, we sought to isolate genetic effects on activity from ‘emotionality’ related measures such as anxiety. Factor loadings from the principal component analysis (Table S12) reveal that the first factor can be interpreted as activity related, and accounts for 27.8% of the variance, whereas the remaining factors capture various facets of anxiety and depression related behaviors. QTL mapping was performed for each component (Fig. S1) For example, PC2, which accounts for 10.3% of the variance and has high loadings for visual cliff avoidance, maps to a significant QTL on chromosome 14, as does a suggestive QTL on distal chromosome 5. The chromosome 14 QTL was found for the simple measure of this phenotype. PC3, which accounts for 8.3% of the variance and has high loadings on fecal boli and poor habituation to the anxiety tests (increased slopes), appears to be influenced by multiple loci, though a single suggestive locus on proximal chromosome 10 is detectable. PC4 accounts for 7.7% of the variance, has a high loading on TST immobility and low transitions in the LD test. No QTLs were detectable for this component. PC5, which accounts for 7.4 % of the variance and has positive loadings on TST climbing and open field center time, with a negative loading on TST immobility, suggesting some relation to ‘emotionality’, and maps to chromosomes 11 and 14.

We reanalyzed traits with significant QTL driven by wild-derived alleles using locomotor activity as a covariate (Fig. S2–S6), to evaluate the potential influence of ‘wildness’ on behavior. We detected the same QTL for most traits, indicating that these are not due to polymorphisms that have primary effects on activity. An expected exception is immobility in the open field, for which the QTL on chromosome 6 is reduced to suggestive level of significance with the same allelic effects. The chromosome 2 locus for this trait remains significant when an activity covariate is included in the mapping model. Climbing behavior on the tail suspension test also revealed some changes in its QTL profile, in which the chromosome 6 PWK allele effect is reduced to suggestive significance and two additional suggestive loci are detected. We conclude that this behavior is mediated in part through an effect on activity due to PWK alleles on chromosome 6. For percent time in the light on the light dark test, the previously detected loci are reduced to suggestive significance but are found in the same location, again indicating that their effects are partially accounted for by locomotor activity. In general, mice with wild-derived alleles at QTL do not have systematically elevated locomotor activity that could account for QTL effects (Fig. S7).

## Discussion

The DO population provides extensive new genetic and phenotypic variation for behavioral genetic analysis. Each DO genome consists of a heterozygous mosaic of the eight founder strains representing a unique combination from more than 45 million SNPs and several million structural variants present in the founder strains (Keane *et al*. 2011). This high genetic diversity drives higher levels of behavioral trait variation in the DO compared to other mapping populations. Most QTL effects were explained by a single founder allele, although in some instances, more complex allelic patterns were also detectable.

High-recombination density in the DO is ideal for precise QTL mapping of behavior. In most cases, QTL support intervals were narrowed by matching SNP distribution patterns to estimated allelic effects. Existing HS and AIL provide high mapping resolution with QTL confidence intervals of ∼2 Mb for open-field behaviors and composites of ‘emotionality’ (Demarest *et al*. 2001; Mott *et al*. 2000; Talbot *et al*. 1999). High precision QTL for drug-related behaviors have also been identified in AIL populations (Parker *et al*. 2012a,b). However, these studies required hundreds of mice to fine-map the QTL. With a modest mapping population of 283 DO mice, we identified narrow QTL intervals, most in the 1–3 Mb range, for several behavioral traits.

Behavioral QTLs detected in another study using similar numbers of CC (partially) inbred strains were larger (Philip *et al*. 2011). These included a 15 Mb locus for hot-plate nociception, 9 Mb locus for novelty-induced open-field locomotor behavior, and a 4 Mb locus for average distance from the center of the open field (Philip *et al*. 2011). QTL confidence intervals for behavioral phenotypes obtained from standard intercrosses are often 20–40 cM (∼40-80 Mb), while many can span an entire chromosome (Flint 2003). The effect sizes for the much more precise loci we detected were similar to those reported for light–dark box and open-field activity in F2 crosses (Flint 2003), with each locus accounting for 10.9–13.7% of the trait variance, or 5.4–6.9% in an additive genetic model. The three largest allelic effects are associated with wild-derived alleles.

We identified QTL on chromosomes 2, 4, 6 and 11 associated with various measures from open-field, light–dark box, visual-cliff avoidance and tail-suspension tests. These chromosomes are well populated by previously reported QTL for locomotor activity, drug response, anxiety and stress related behaviors. Our chromosome 2 locus for duration of immobility in the open-field overlaps with *Hylaq1* (Umemori *et al*. 2009), and several ethanol-related loci (*Etohc*, Phillips *et al*. 1994, Saba *et al*. 2006; *Etohila*, Hitzemann *et al*. 1998; *Etohr*, Demarest *et al*. 1999; *Vacq3*, Gill & Boyle, 2005). Our chromosome 4 habituation locus overlaps with *Start2* (Le Roy *et al*. 1999). Previously identified QTL on chromosome 6 for anxiety and depression behaviors do not overlap with our open-field immobility QTL (*Rear1*, Kelly *et al*. 2003; *Hcga4*, Nishi *et al*. 2010; *Axtq2*, Singer *et al*. 2005). However, we did find a locus for activity in the bottom of the visual-cliff on chromosome 11 that overlaps with several drug-related locomotor activity loci (*Eiwa2*, Drews *et al*. 2010; *Nilac2*, Gill & Boyle 2005; *Etax10*, Kirstein *et al*. 2002; *Marq3*, Palmer *et al*. 2005), and novelty-induced locomotor activity loci (*Nsila8*, Gill & Boyle 2005). An additional three suggestive QTL were found on chromosomes 8, 14, and 19. The anxiety behavior locus on chromosome 8 overlapped with an anxiety locus, *Lacanx1* (Bailey *et al*. 2008), and the novelty and stress induced locomotor activity loci, *Nsila6 and Nsila7* (Gill & Boyle 2005). A previous anxiety-related locus on chromosome 14, *Axtofd3*, overlaps with our visual-cliff QTL (Turri *et al*. 2001; Henderson *et al*. 2004). In addition, the immobility locus on chromosome 19 overlaps with several loci previously associated with ethanol preference (*Alcp23/24*, Gill & Boyle 1998) and another for locomotor activity (*Bslm2*, Hitzemann *et al*. 2000). Thus, we observe some convergence between previous behavioral studies of various mouse populations and our initial characterization of the DO. However, the previously reported QTL generally span large regions, and the extent of similarity to the trait measured here varies. Ultimately the high precision of the DO population will enable identification of pleiotropic regulators of behavior and reduction of linkage-related correlation of phenotypic values and overlapping QTLs.

Not all previously reported QTL were replicated. For example, the chromosome 4 locus for novelty-induced locomotor activity (0–4 min in open-field) identified in both the BXD and CC was not detected in our DO sample. Conversely, a chromosome 8 locus for light time in the light–dark box was detected only in the DO. Several factors may account for discrepancies between studies, including different testing environments, multiple locus effects, and the allelic distribution in the populations. More advanced statistical models that account for dominance effects, polygenic influences, and genetic or environmental epistasis could be expected to reveal additional loci.

One might anticipate a large number of significant QTL in the DO, particularly because many of traits showed high proportion of genetic variation among founders. In total, more significant loci were detected than expected by chance. Eight suggestive and significant QTL were mapped for traits that had a wide range of heritability. The QTL peaks had large effect sizes. For many traits (21/38), multiple significant and/or suggestive peaks were detected (e.g. percent time light and climbing frequency). With greater sample sizes and detailed modeling afforded by this extensible population, these traits can be approached more comprehensively.

Allele effects associated with founder haplotypes in the DO can be compared to complete catalogs of sequence variants to identify possible causal variants. For many QTL, the allele effects were dichotomous suggesting that a single diallelic variant is responsible. In other cases, complex multi-state allele effects suggest that multiple variants are involved, perhaps representing allelic series of a single causal gene. Using this strategy, we narrowed the largest support interval of 7 Mb to three regions spanning less than 500 kb – the only regions that harbor private NZO/H1LtJ variants, some of which are in genes previously associated with related behavioral traits. For a few loci, a single wild-derived allele differs from all others. Due to the high levels of divergence of the wild-derived founders, the entire QTL support interval is usually polymorphic compared to the other strains (Kelada *et al*. 2012). We identified three QTL with this pattern. One of these (center time slope on chromosome 4) could not be narrowed and two others (immobility and climbing both on chromosome 6 but at different loci) contained regions populated entirely by SNPs unique to the respective wild-derived alleles preventing any further narrowing.

Validation of QTLs detected in any single study is a critical next step. The high precision of the mapping results from the DO facilitates validation by limiting the list of plausible candidates. Unfortunately, as is the case with any mapping population comprised of unique individuals, direct replication of the experiment is not possible. Access to the same allelic variants in the CC inbred strains provides a direct route to experimental validation. Knock-in transgenics made using zinc finger (Bibikova *et al*. 2003) or TAL effector (Christian *et al*. 2010) endonucleases and other technologies can also be used for validation of specific loci, and may be the most effective way to confirm single allelic effects. The narrow QTL support intervals obtained using the DO, can make directly proceeding to single-locus complementation tests and allele-specific validation more efficient and cost-effective than additional confirmatory genetic experiments.

Inbred laboratory strains display sufficient behavioral variation for QTL detection. However, it has been speculated that historical inbreeding selected for ease of handling. The kinds of measures obtained in the present study could have been targets of domesticating selection. As we previously reported, domestication likely operated on multiple loci throughout the genome, retaining different docility and wildness alleles (Philip *et al*. 2011). This motivates the question of whether heterozygous DO mice are amenable to classical and pharmacologically validated biomedical behavioral tests. Although aggressive behavior was sometimes a housing issue, there were few notable handling issues or concerns for testing validity and it did not systematically associate with the traits in this study (not shown).

Our results using the DO are consistent with studies in *Mus musculus molossinus* derived lines, indicating that wild-derived inbred strains are amenable to standard behavioral testing (Koide *et al*. 2011). Traits related to anxiety, depression, and habituation in the wild-derived progenitor strains and the DO mice were mapped using standard open-field, light–dark box, visual-cliff, and tail-suspension tests, where many results were consistent with previous studies. Our results do not indicate qualitatively different behaviors in mice with wild-derived alleles at QTL, with the exception of climbing during tail-suspension. While climbing is usually considered to be a confounding behavior, it appears to have a robust genetic origin, which we mapped to a PWK/PhJ allele, and may resemble escape or avoidance (Mayorga & Lucki 2001; Swiergiel & Dunn 2006). We interpret our results to indicate that the effects of domesticating selection have reduced the available variation for studies of anxiety related behavior in commonly used mouse populations, and by segregating the ‘lost’ alleles back into the laboratory population they are detectable sources of variation from among a broader quantitative distribution of behavior.

The DO represents a powerful system for comparatively fast, cost-effective, high precision QTL mapping. Using 280 mice in early outbreeding generations G4-5, we were able to map QTL with a resolution ranging from 1–7 Mb in ∼6 months, in contrast to an endeavor that typically requires multiple mapping and fine-mapping studies over a period of years. As outbreeding progresses, it is expected that mapping resolution will continue to improve (Svenson *et al*. 2012). Putative regulatory loci mapped in the DO can be validated with the complementary resources of the CC or their intercross progeny (Churchill *et al*. 2012). The inclusion of wild-derived alleles raises concern that conventional behavioral assays may not perform well on this population. The atypical or ‘inappropriate’ behaviors that are observed in a subset of mice on certain tests are interesting variants that can be mapped, but require careful analysis and interpretation. The increased genetic diversity in the DO introduced by novel allele combinations provides a wide spectrum of behavior extending far beyond that of historical genetic mouse populations, and holds great promise for the genetic dissection of complex behavioral traits.

## Supporting Information

**Table S1:** Summary statistics of behaviors in open-field area of progenitor and DO mice

**Table S2:** Summary statistics of behaviors in light–dark box of progenitor and DO mice

**Table S3:** Summary statistics of behaviors in visual-cliff avoidance arena of progenitor and DO mice

**Table S4:** Summary statistics of behaviors during tail-suspension test of progenitor and DO mice

**Table S5:** Genes within QTL interval on chromosome 4 for center time slope

**Table S6:** Genes within QTL interval on chromosomes 2, 6 and 19 for percent time immobile

**Table S7:** Genes within QTL interval on chromosome 11 for light time slope

**Table S8:** Genes within QTL interval on chromosome 8 for percent light time

**Table S9:** Genes within QTL interval on chromosome 14 for bottom distance traveled

**Table S10:** Genes within QTL interval on chromosome 6 for climbing

**Table S11:** Correlations among activity measures and traits with significant QTLs

**Table S12:** Principal components analysis

**Figure S1:** QTL mapping of principal components 1–6.

**Figure S2:** QTL mapping conditioned on locomotor activity – center time slope in open-field.

**Figure S3:** QTL mapping conditioned on locomotor activity – immobility in the open-field.

**Figure S4:** QTL mapping conditioned on locomotor activity – climbing frequency during tail-suspension test.

**Figure S5:** QTL mapping conditioned on locomotor activity – percent time in light in the light–dark box.

**Figure S6:** QTL mapping conditioned on locomotor activity – time spent in the bottom area of the visual cliff (ratio).

**Figure S7:** QTL alleles and the relationship between open field activity and other behavioral measures. Individual mice with wild-derived QTL alleles do not have extreme values on these correlated behaviors.
