# Supplementary material for: High-precision genetic mapping of behavioral traits in the diversity outbred mouse population
Source: Genes Brain Behav. 2013 Mar 20;12(4):424–37. doi: 10.1111/gbb.12029 (PMC3709837; doi:10.1111/gbb.12029)
Supplement: Supplementary file 1 [file gbb0012-0424-SD1.doc]

**Supplemental table 1:** Summary statistics of behaviors in open-field area of progenitor and DO mice.

|  |  | **Diversity Outbred** | | **129S1/SvImJ** | | **A/J** | | **C57BL/6J** | | **Cast/EiJ** | | **NOD/ShiLtJ** | | **NZO/H1LTJ** | | **PWK/PhJ** | | **WSB/EiJ** | |
| --- | --- | --- | --- | --- | --- | --- | --- | --- | --- | --- | --- | --- | --- | --- | --- | --- | --- | --- | --- |
| **Traits** | **Statistics** | **Female** | **Male** | **Female** | **Male** | **Female** | **Male** | **Female** | **Male** | **Female** | **Male** | **Female** | **Male** | **Female** | **Male** | **Female** | **Male** | **Female** | **Male** |
| **Open Field** | N | 144 | 139 | 8 | 8 | 8 | 8 | 8 | 8 | 8 | 8 | 8 | 8 | 8 | 8 | 8 | 8 | 8 | 8 |
| Distance (cm) traveled in first 4min | Mean ± SEM | 1412.42 ± 42.62 | 1356.11 ± 41.91 | 957.89 ± 122.59 | 736.69 ± 84.81 | 301.84 ± 18.56 | 335.7 ± 37.84 | 1774.2 ± 124.35 | 2038.83 ± 101.55 | 2898.91 ± 140.05 | 2104.63 ± 237.84 | 2281.74 ± 185.27 | 2085.14 ± 199.66 | 741.01 ± 117.95 | 612.02 ± 45.24 | 1385.27 ± 166.05 | 1551.82 ± 60.9 | 2479.51 ± 170.54 | 2596.51 ± 177.15 |
|  | Min — Max | 346.53 — 2982.08 | 387.99 — 2628.46 | 553.79 — 1560.8 | 370.98 — 1120.43 | 196.67 — 368.41 | 224.46 — 500.89 | 1380.67 — 2551.15 | 1670.68 — 2564.25 | 2233.13 — 3435.93 | 1027.42 — 3405.73 | 1580.2 — 3352.49 | 799.55 — 2794.59 | 493.5 — 1522.42 | 463.64 — 799.81 | 281.56 — 1733.2 | 1264.07 — 1807.18 | 1771.48 — 3281.78 | 2102.59 — 3634.91 |
| Total distance (cm) traveled | Mean ± SEM | 5871.48 ± 168.22 | 5801.74 ± 161.01 | 3364.67 ± 459.07 | 2932.55 ± 260.52 | 1274.45 ± 97.63 | 1351.27 ± 66.76 | 7214.78 ± 360.96 | 7566.2 ± 402.9 | 11783.83 ± 447.7 | 9752.64 ± 902.49 | 9403.58 ± 614.26 | 8697.02 ± 471.46 | 3813.79 ± 453.86 | 3179.83 ± 129.26 | 7552.93 ± 405.39 | 6843.01 ± 413.6 | 12126.94 ± 1489.16 | 11677 ± 813.63 |
|  | Min — Max | 2094.25 — 15891.94 | 1895.46 — 12728.03 | 1405.52 — 5513.76 | 1786.08 — 3722.46 | 791.53 — 1646.38 | 1084.79 — 1672.01 | 5988.44 — 9223.17 | 5763.95 — 8860.27 | 9814.47 — 13952.47 | 7938.56 — 15713 | 6863.81 — 11720.15 | 7075.91 — 10329.95 | 2498.67 — 5761.07 | 2526.86 — 3742.22 | 5342.85 — 8657.07 | 4967.56 — 9168.35 | 6645.58 — 18212.33 | 8343.73 — 14802.11 |
| Distance traveled slope | Mean ± SEM | -0.09 ± 0.01 | -0.08 ± 0.01 | -0.18 ± 0.04 | -0.1 ± 0.06 | -0.02 ± 0.04 | 0 ± 0.05 | -0.1 ± 0.01 | -0.12 ± 0.02 | -0.11 ± 0.01 | -0.05 ± 0.03 | -0.08 ± 0.02 | -0.04 ± 0.04 | -0.02 ± 0.04 | -0.07 ± 0.04 | 0.03 ± 0.06 | -0.1 ± 0.03 | -0.09 ± 0.09 | -0.1 ± 0.02 |
|  | Min — Max | -0.55 — 0.2 | -0.6 — 0.23 | -0.35 — -0.04 | -0.28 — 0.26 | -0.16 — 0.12 | -0.2 — 0.29 | -0.14 — -0.03 | -0.24 — -0.06 | -0.19 — -0.07 | -0.13 — 0.1 | -0.15 — -0.01 | -0.16 — 0.18 | -0.15 — 0.22 | -0.26 — 0.07 | -0.09 — 0.41 | -0.28 — 0.02 | -0.65 — 0.08 | -0.22 — -0.02 |
| % time in corners | Mean ± SEM | 47.98 ± 1.27 | 46.36 ± 1.05 | 10.87 ± 4.56 | 4.21 ± 2.58 | 26.93 ± 8.51 | 18.42 ± 6.9 | 4.35 ± 0.44 | 5.07 ± 0.74 | 9.06 ± 1.61 | 10.9 ± 1.81 | 7.39 ± 1.56 | 7.18 ± 1.47 | 9.26 ± 1.81 | 4.69 ± 1.58 | 9.52 ± 1.59 | 15.94 ± 2.21 | 15.12 ± 3.52 | 10.26 ± 1.72 |
|  | Min — Max | 7.63 — 93.88 | 16.1 — 89.68 | 0.44 — 31.23 | 1.02 — 22.24 | 0 — 63.5 | 0.27 — 54.2 | 2.76 — 6.32 | 2.06 — 8.05 | 3.59 — 17.51 | 2 — 16.95 | 1.83 — 14.12 | 2.82 — 15.38 | 3.27 — 17.35 | 0.93 — 14.82 | 1.67 — 14.55 | 5.99 — 24.76 | 2.67 — 34.26 | 3.31 — 16.92 |
| Time in corners slope | Mean ± SEM | 0.02 ± 0.02 | 0.01 ± 0.01 | 0.14 ± 0.45 | -0.48 ± 0.34 | 0.89 ± 0.38 | 1.03 ± 0.4 | -0.28 ± 0.06 | -0.18 ± 0.05 | -0.14 ± 0.06 | -0.12 ± 0.06 | 0.05 ± 0.1 | -0.11 ± 0.12 | 0.21 ± 0.29 | -0.04 ± 0.32 | 0.15 ± 0.21 | 0.06 ± 0.09 | 0.06 ± 0.09 | -0.02 ± 0.09 |
|  | Min — Max | -0.34 — 2.25 | -0.75 — 0.98 | -1.52 — 2.16 | -1.7 — 1.31 | -0.47 — 2.66 | -1.35 — 2.57 | -0.57 — -0.01 | -0.39 — 0.01 | -0.37 — 0.1 | -0.35 — 0.12 | -0.56 — 0.3 | -0.7 — 0.3 | -1.24 — 1.25 | -1.13 — 1.16 | -0.21 — 1.55 | -0.3 — 0.4 | -0.24 — 0.58 | -0.5 — 0.25 |
| % time in periphery | Mean ± SEM | 14.68 ± 0.77 | 11.75 ± 0.69 | 41.72 ± 9.37 | 48.42 ± 8.1 | 51.02 ± 9.53 | 57.96 ± 9.33 | 30.65 ± 2.07 | 23.63 ± 1.94 | 51.36 ± 1.84 | 48.31 ± 2.07 | 35.92 ± 2.32 | 35.02 ± 3.35 | 50.91 ± 4.09 | 33.98 ± 4.05 | 57 ± 2.45 | 56.49 ± 2.67 | 51.77 ± 5.2 | 55.8 ± 2.3 |
|  | Min — Max | 0 — 55.31 | 0.56 — 61.13 | 7.8 — 86.05 | 20.41 — 91.33 | 0 — 87.14 | 7.87 — 92.12 | 20.85 — 39.57 | 14.64 — 31.16 | 43.61 — 57.03 | 43.52 — 60.15 | 26.32 — 43.48 | 19.86 — 44.59 | 37.44 — 70.92 | 13.82 — 47.19 | 47.24 — 65.24 | 48.75 — 68.37 | 30.04 — 73 | 43.91 — 65.41 |
| Time in periphery slope | Mean ± SEM | -0.22 ± 0.07 | -0.12 ± 0.08 | -0.14 ± 0.09 | 0.31 ± 0.27 | 0.19 ± 0.32 | 0.38 ± 0.25 | -0.17 ± 0.04 | -0.14 ± 0.04 | -0.07 ± 0.03 | -0.03 ± 0.03 | 0.05 ± 0.02 | -0.05 ± 0.03 | 0.04 ± 0.05 | -0.15 ± 0.15 | 0.02 ± 0.06 | 0 ± 0.03 | -0.15 ± 0.19 | 0.02 ± 0.03 |
|  | Min — Max | -2.6 — 2.36 | -2.46 — 2.63 | -0.56 — 0.19 | -0.43 — 1.92 | -0.74 — 2.16 | -0.31 — 1.66 | -0.36 — -0.01 | -0.3 — -0.01 | -0.17 — 0.01 | -0.2 — 0.09 | -0.08 — 0.14 | -0.19 — 0.05 | -0.17 — 0.25 | -1.04 — 0.26 | -0.16 — 0.42 | -0.15 — 0.1 | -1.49 — 0.09 | -0.13 — 0.15 |
| % time in center | Mean ± SEM | 3.41 ± 0.38 | 3.14 ± 0.18 | 2.37 ± 0.85 | 2.81 ± 1.48 | 6.17 ± 4.5 | 2.74 ± 1.49 | 3.5 ± 0.64 | 3.79 ± 0.56 | 2.05 ± 0.29 | 2.72 ± 0.73 | 4.52 ± 0.29 | 4.85 ± 0.82 | 1.22 ± 0.64 | 1.5 ± 0.29 | 2.42 ± 0.44 | 1.48 ± 0.36 | 3.63 ± 2.24 | 1.6 ± 0.21 |
|  | Min — Max | 0.01 — 42.76 | 0.02 — 15 | 0.01 — 6.75 | 0.01 — 12.46 | 0.01 — 36.25 | 0.01 — 12.63 | 1.72 — 7.01 | 1.75 — 6.76 | 0.94 — 3.58 | 1.1 — 7.35 | 3.17 — 5.58 | 2.4 — 8.41 | 0.01 — 5.18 | 0.36 — 2.78 | 1.07 — 4.61 | 0.41 — 3.58 | 0.57 — 19.19 | 0.96 — 2.8 |
| Time in center slope | Mean ± SEM | -0.35 ± 0.06 | -0.43 ± 0.05 | -0.44 ± 0.24 | -0.63 ± 0.31 | -0.93 ± 0.36 | -1.02 ± 0.27 | 0.21 ± 0.1 | 0.11 ± 0.09 | 0.08 ± 0.09 | 0.1 ± 0.09 | -0.09 ± 0.06 | 0.07 ± 0.11 | 0.17 ± 0.33 | -0.21 ± 0.2 | 0.08 ± 0.2 | -0.13 ± 0.17 | -0.19 ± 0.12 | -0.18 ± 0.15 |
|  | Min — Max | -2.07 — 2.12 | -1.87 — 0.69 | -1.35 — 0.39 | -1.96 — 0.59 | -2.74 — 0 | -2.4 — 0 | -0.33 — 0.58 | -0.29 — 0.57 | -0.38 — 0.43 | -0.15 — 0.58 | -0.31 — 0.08 | -0.25 — 0.77 | -1.57 — 1.25 | -0.92 — 0.79 | -0.83 — 1.04 | -0.94 — 0.67 | -0.88 — 0.19 | -1.09 — 0.12 |
| % time immobile | Mean ± SEM | 81.31 ± 0.96 | 85.97 ± 0.78 | 79.25 ± 11.08 | 82.4 ± 5.84 | 96.57 ± 1.83 | 97.05 ± 0.92 | 55.91 ± 5.94 | 54.7 ± 6.72 | 31.76 ± 4.73 | 41.02 ± 8.54 | 42.44 ± 7.31 | 44.69 ± 5.6 | 76.73 ± 9.88 | 85.15 ± 5.13 | 50 ± 10.21 | 52.86 ± 5.92 | 44.28 ± 9.27 | 39.63 ± 2.51 |
|  | Min — Max | 38.09 — 99.22 | 52.49 — 99.4 | 59.32 — 97.55 | 72.69 — 90.77 | 93.77 — 99.11 | 95.23 — 98.12 | 48.95 — 62.91 | 45.91 — 65.55 | 27.57 — 38.24 | 25.58 — 51.07 | 32.83 — 53.59 | 36.61 — 55.07 | 62.63 — 86.91 | 77.64 — 91.3 | 37.38 — 63.29 | 40.84 — 58.98 | 34.96 — 61.66 | 35.77 — 42.9 |
|  |  |  |  |  |  |  |  |  |  |  |  |  |  |  |  |  |  |  |  |
