# Supplementary material for: High-precision genetic mapping of behavioral traits in the diversity outbred mouse population
Source: Genes Brain Behav. 2013 Mar 20;12(4):424–37. doi: 10.1111/gbb.12029 (PMC3709837; doi:10.1111/gbb.12029)
Supplement: Supplementary file 2 [file gbb0012-0424-SD2.doc]

**Supplemental table 2:** Summary statistics of behaviors in light-dark box of progenitor and DO mice.

|  |  |  |  | **Strain** | | | | | | | | | | | | | | | |
| --- | --- | --- | --- | --- | --- | --- | --- | --- | --- | --- | --- | --- | --- | --- | --- | --- | --- | --- | --- |
|  |  | **Diversity Outbred** | | **129S1/SvImJ** | | **A/J** | | **C57BL/6J** | | **Cast/EiJ** | | **NOD/ShiLtJ** | | **NZO/H1LTJ** | | **PWK/PhJ** | | **WSB/EiJ** | |
| **Trait** | **Statistics** | **Female** | **Male** | **Female** | **Male** | **Female** | **Male** | **Female** | **Male** | **Female** | **Male** | **Female** | **Male** | **Female** | **Male** | **Female** | **Male** | **Female** | **Male** |
| **Light-dark box** | N | 143 | 138 | 7 | 7 | 8 | 6 | 8 | 8 | 8 | 4 | 8 | 8 | 8 | 8 | 7 | 7 | 8 | 8 |
| Distance (cm) traveled in light | Mean ± SEM | 2980.63 ± 128.48 | 2908.17 ± 132.03 | 468.39 ± 169.73 | 1918.03 ± 816.59 | 405.35 ± 132.12 | 449.06 ± 142.68 | 4476.58 ± 256.49 | 3360.8 ± 234.48 | 5899.45 ± 480.92 | 4492.87 ± 1383.29 | 6038.08 ± 476.63 | 6054.75 ± 375.41 | 1876.85 ± 400.58 | 2075.22 ± 183.05 | 2564.61 ± 588.17 | 1219.95 ± 319.96 | 3330.03 ± 316.61 | 4115.13 ± 273.82 |
|  | Min — Max | 47.23 — 6730.48 | 70.25 — 7195.21 | 23.28 — 1297.04 | 110.15 — 4751.77 | 124.04 — 1250.92 | 140.18 — 1116.73 | 3301.15 — 5411 | 2508.54 — 4094.16 | 3312.1 — 7769.19 | 1180.57 — 7926.98 | 3480.29 — 7445.12 | 4923.5 — 8389.14 | 495.4 — 4066.87 | 1477.84 — 2814.97 | 254.07 — 4855.84 | 396.19 — 2859.67 | 2353.91 — 4666.02 | 3017 — 5287.8 |
| Number of light-dark transitions | Mean ± SEM | 31.34 ± 1.32 | 26.94 ± 1.12 | 15 ± 5.88 | 31.57 ± 8.64 | 47.5 ± 14.18 | 25.5 ± 10.95 | 32 ± 10.59 | 27.38 ± 10.32 | 21.75 ± 7.86 | 54 ± 17.14 | 64 ± 14.92 | 37.88 ± 7 | 86.25 ± 14.52 | 7.38 ± 2.15 | 37.71 ± 9.84 | 30 ± 9.8 | 55.38 ± 9.77 | 34.5 ± 7.18 |
|  | Min — Max | 0 — 78 | 5 — 68 | 3 — 49 | 1 — 58 | 2 — 139 | 0 — 63 | 0 — 74 | 0 — 71 | 2 — 56 | 23 — 103 | 36 — 166 | 12 — 73 | 42 — 166 | 0 — 16 | 0 — 74 | 6 — 63 | 3 — 95 | 14 — 73 |
| % time in light | Mean ± SEM | 36.85 ± 1.35 | 38.63 ± 1.44 | 5.37 ± 2.17 | 22.82 ± 9.12 | 3.76 ± 2.09 | 6.08 ± 3.92 | 46.06 ± 3.23 | 61.13 ± 7.83 | 53.18 ± 2.94 | 35.75 ± 9.01 | 54.13 ± 4.92 | 61.03 ± 5.87 | 26.24 ± 6.84 | 76.22 ± 8.2 | 42.07 ± 6.41 | 55.77 ± 11.67 | 64.54 ± 8.55 | 46.89 ± 3.13 |
|  | Min — Max | 0.04 — 84.17 | 1.21 — 67.3 | 0.02 — 12.44 | 0.64 — 52.04 | 0.13 — 16.79 | 0.21 — 24.91 | 28.14 — 57.7 | 24.82 — 97.1 | 45.98 — 71.72 | 10.69 — 52.56 | 37.19 — 75.35 | 40.96 — 95.52 | 4.35 — 57.54 | 45.96 — 98.17 | 20.31 — 67.37 | 24.12 — 90.61 | 37.97 — 93.9 | 35.74 — 59.87 |
| % time in light first 4mins | Mean ± SEM | 7.06 ± 0.27 | 7.28 ± 0.33 | 3.62 ± 1.63 | 4.65 ± 1.03 | 1.12 ± 0.58 | 4.09 ± 3.08 | 8.3 ± 0.53 | 8.78 ± 1.91 | 7.39 ± 0.97 | 5.1 ± 2.1 | 10.43 ± 0.42 | 11.25 ± 1.08 | 4.98 ± 0.97 | 13.32 ± 2.18 | 9.13 ± 2.1 | 12.32 ± 1.75 | 9.75 ± 1.9 | 5.64 ± 0.91 |
|  | Min — Max | 0.01 — 15.32 | 0.04 — 19.75 | 0.02 — 11.11 | 0.64 — 7.97 | 0.13 — 5.07 | 0.21 — 19.44 | 5.64 — 10.23 | 4.4 — 19.53 | 3.32 — 10.76 | 0.33 — 10.13 | 8.33 — 12.16 | 8.87 — 18.17 | 1.06 — 8.9 | 1.57 — 18.47 | 0.55 — 18.9 | 5.5 — 18.72 | 2.32 — 17.18 | 1.62 — 8.06 |
| Time (s) in light slope | Mean ± SEM | -0.08 ± 0.05 | -0.13 ± 0.05 | -1.41 ± 0.23 | -0.98 ± 0.39 | -0.93 ± 0.37 | -1.63 ± 0.33 | -0.03 ± 0.03 | 0.1 ± 0.04 | 0.03 ± 0.04 | 0.26 ± 0.2 | -0.03 ± 0.03 | -0.01 ± 0.01 | -0.43 ± 0.21 | 0.03 ± 0.11 | 0.12 ± 0.22 | -0.58 ± 0.44 | 0.11 ± 0.03 | 0.04 ± 0.08 |
|  | Min — Max | -2.37 — 1.74 | -2.4 — 1.84 | -2.18 — -0.66 | -2.41 — 0.15 | -1.94 — 0.86 | -2.89 — -0.54 | -0.14 — 0.14 | -0.01 — 0.32 | -0.11 — 0.25 | -0.02 — 0.85 | -0.19 — 0.05 | -0.05 — 0.06 | -1.73 — 0.18 | -0.58 — 0.54 | -0.34 — 1.07 | -2.94 — 0.14 | 0 — 0.24 | -0.24 — 0.37 |
|  |  |  |  |  |  |  |  |  |  |  |  |  |  |  |  |  |  |  |  |
|  |  |  |  |  |  |  |  |  |  |  |  |  |  |  |  |  |  |  |  |
