# Supplementary material for: High-precision genetic mapping of behavioral traits in the diversity outbred mouse population
Source: Genes Brain Behav. 2013 Mar 20;12(4):424–37. doi: 10.1111/gbb.12029 (PMC3709837; doi:10.1111/gbb.12029)
Supplement: Supplementary file 3 [file gbb0012-0424-SD3.doc]

**Supplemental table 3:** Summary statistics of behaviors in visual-cliff avoidance arena of progenitor and DO mice.

|  |  | **Strain** | | | | | | | | | | | | | | | | | |
| --- | --- | --- | --- | --- | --- | --- | --- | --- | --- | --- | --- | --- | --- | --- | --- | --- | --- | --- | --- |
|  |  | **Diversity Outbred** | | **129S1/SvImJ** | | **A/J** | | **C57BL/6J** | | **Cast/EiJ** | | **NOD/ShiLtJ** | | **NZO/H1LTJ** | | **PWK/PhJ** | | **WSB/EiJ** | |
| **Traits** | **Statistics** | **Female** | **Male** | **Female** | **Male** | **Female** | **Male** | **Female** | **Male** | **Female** | **Male** | **Female** | **Male** | **Female** | **Male** | **Female** | **Male** | **Female** | **Male** |
| **Visual-cliff avoidance arena** | N | 142 | 138 | 8 | 7 | 4 | 5 | 8 | 7 | 8 | 8 | 8 | 8 | 8 | 8 | 8 | 8 | 8 | 8 |
| Total distance (cm) traveled | Mean ± SEM | 9010.04 ± 263.72 | 8527.07 ± 245.55 | 2350.33 ± 192.5 | 2099.63 ± 193.47 | 2173.2 ± 326.34 | 2026.64 ± 203.96 | 6262.28 ± 374.57 | 6943.65 ± 769.26 | 10548.31 ± 559.34 | 8934.07 ± 1143.51 | 12013.48 ± 714.68 | 8929.03 ± 644.72 | 3708.55 ± 288.27 | 3108.36 ± 132.01 | 7197.09 ± 579.74 | 7805.57 ± 471.61 | 11327.22 ± 1002.12 | 9012.24 ± 825.43 |
|  | Min — Max | 2616.84 — 18350.91 | 2329.61 — 16550.77 | 1741.45 — 3208.08 | 1209.19 — 2677.35 | 1737.12 — 3121.86 | 1488.07 — 2663.51 | 4417.68 — 8107.2 | 4386.83 — 9719.62 | 8774.02 — 13162.84 | 4734.74 — 15405.6 | 8320.49 — 14173.15 | 6650.54 — 11733.88 | 2582.8 — 4675.81 | 2676.32 — 3752.93 | 4972.93 — 9770.95 | 6398.47 — 10715.19 | 8490.44 — 16282.63 | 6365.26 — 12321.55 |
| Total duration (s) immobile | Mean ± SEM | 952.42 ± 9.84 | 991.84 ± 8.37 | 1089.82 ± 24.68 | 1104.64 ± 9.55 | 1075.46 ± 45.36 | 1113.27 ± 14.32 | 788.17 ± 30.44 | 750.82 ± 50.85 | 525.72 ± 26.1 | 650.53 ± 51.33 | 494.54 ± 23.37 | 639.91 ± 24.51 | 1013.76 ± 21.75 | 1069.43 ± 9.49 | 754.31 ± 35.67 | 735.43 ± 25.6 | 601.68 ± 33.11 | 663.25 ± 27.78 |
|  | Min — Max | 679.61 — 1177.04 | 714.78 — 1175.04 | 948.28 — 1153.15 | 1083.02 — 1151.69 | 940.81 — 1138.54 | 1079.95 — 1157.69 | 638.04 — 938 | 561.63 — 921.59 | 414.48 — 635.84 | 373.97 — 852.25 | 391.93 — 584.05 | 542.94 — 757.49 | 948.88 — 1108.04 | 1033.23 — 1103.57 | 594.53 — 893.96 | 582.98 — 811.81 | 418.75 — 698.9 | 567.43 — 812.55 |
| Total transitions between top and bottom | Mean ± SEM | 53.82 ± 2.7 | 45.32 ± 2.59 | 5.75 ± 1.01 | 4.71 ± 1.15 | 7 ± 3 | 2.6 ± 0.81 | 37.75 ± 4.39 | 45.43 ± 6.84 | 63.88 ± 4.32 | 52.38 ± 9.29 | 70.63 ± 5.77 | 54.13 ± 5.58 | 14.63 ± 2.7 | 12.25 ± 0.96 | 43.38 ± 6.53 | 49.88 ± 4.56 | 66.5 ± 8.87 | 48 ± 5.41 |
|  | Min — Max | 1 — 134 | 1 — 165 | 3 — 11 | 0 — 8 | 2 — 14 | 0 — 5 | 18 — 58 | 25 — 76 | 49 — 79 | 26 — 105 | 44 — 99 | 25 — 79 | 6 — 24 | 9 — 18 | 22 — 70 | 31 — 71 | 40 — 110 | 32 — 70 |
| Distance (cm) traveled in top | Mean ± SEM | 5463.81 ± 138.95 | 5182.58 ± 128.65 | 1598.01 ± 174.73 | 1274.19 ± 113.81 | 1368.88 ± 293.82 | 1429.42 ± 201.24 | 3031.86 ± 271.35 | 3558.09 ± 448.97 | 4214.09 ± 342.48 | 3997.07 ± 640.2 | 5996.52 ± 437.84 | 4400.11 ± 420.68 | 1940.52 ± 228.93 | 1655.6 ± 108.52 | 3481.34 ± 213.22 | 3419.87 ± 270.68 | 5069.99 ± 508.38 | 3828.82 ± 343.98 |
|  | Min — Max | 2267.95 — 11005.7 | 2065.15 — 10343.52 | 630.14 — 2177.52 | 947.79 — 1659.86 | 969.86 — 2223.74 | 837.08 — 1964.49 | 1802.52 — 4552.16 | 2476.43 — 5422.11 | 2628.27 — 5865.2 | 1576.91 — 7155.95 | 4456.92 — 8003.77 | 3171.87 — 6671.96 | 1241.9 — 2808.09 | 1193.18 — 2090.04 | 2525.85 — 4207.63 | 2342.07 — 4496.82 | 3231.86 — 6959.42 | 2652.02 — 5504.51 |
| Distance (cm) traveled in top first 4min | Mean ± SEM | 1578.2 ± 36.56 | 1509.32 ± 36.11 | 637.79 ± 55.82 | 338.69 ± 89.87 | 324.17 ± 91.21 | 308.58 ± 85.24 | 900.91 ± 90.62 | 840.8 ± 57.28 | 815.88 ± 54.97 | 785.03 ± 101.54 | 1180.1 ± 123.32 | 810.39 ± 129.89 | 442.02 ± 40.84 | 365.32 ± 36.82 | 758.04 ± 53.2 | 753.87 ± 79.46 | 900.82 ± 96.63 | 597.92 ± 43.52 |
|  | Min — Max | 652.84 — 3190.61 | 534.9 — 2660.97 | 373.78 — 859.46 | 39.82 — 574.26 | 131.87 — 571.72 | 12.78 — 529.61 | 608.51 — 1380.12 | 642.66 — 1046.21 | 566.1 — 1007.2 | 333.69 — 1199.12 | 657.48 — 1712.15 | 371.73 — 1367.18 | 281.18 — 628.4 | 233.31 — 538.05 | 510.18 — 932.59 | 475.51 — 1135.78 | 493.23 — 1414.43 | 393.73 — 754.44 |
| Entries into top | Mean ± SEM | 65.92 ± 3.1 | 57.5 ± 3 | 48.38 ± 20.1 | 61 ± 44.35 | 31.5 ± 14.48 | 11.8 ± 2.89 | 67.13 ± 6.54 | 85.29 ± 11.62 | 138.13 ± 17.33 | 101.25 ± 16.98 | 146 ± 12.07 | 94 ± 10.09 | 40 ± 10.68 | 23.25 ± 4.01 | 97.63 ± 13.29 | 88.75 ± 12.13 | 186.5 ± 17.7 | 131.38 ± 22.69 |
|  | Min — Max | 6 — 157 | 6 — 189 | 10 — 150 | 5 — 326 | 9 — 74 | 5 — 20 | 51 — 108 | 51 — 140 | 83 — 216 | 53 — 170 | 99 — 187 | 46 — 121 | 10 — 98 | 16 — 45 | 30 — 139 | 48 — 129 | 121 — 250 | 41 — 225 |
| Duration (s) immobile in top | Mean ± SEM | 705.83 ± 19.87 | 702.19 ± 20.31 | 849.48 ± 115.95 | 798.06 ± 132.49 | 948.72 ± 60.68 | 909.2 ± 155.35 | 419.27 ± 25.45 | 456.85 ± 53.61 | 200.28 ± 12.46 | 274.92 ± 36.71 | 250.33 ± 18.64 | 302.99 ± 50.73 | 629.88 ± 57.3 | 705.96 ± 60.71 | 431.09 ± 53.11 | 361.54 ± 46.86 | 280.38 ± 27.88 | 287.92 ± 23.99 |
|  | Min — Max | 265.33 — 1175.38 | 183.65 — 1170.37 | 242.44 — 1140.07 | 190.26 — 1091.69 | 786.19 — 1055.12 | 302.04 — 1155.82 | 322.39 — 555.62 | 342.28 — 751.28 | 160.69 — 266.87 | 166.3 — 499.63 | 185.59 — 335.34 | 179.45 — 606.47 | 451.32 — 940.27 | 447.71 — 918.79 | 232.83 — 633.37 | 222.56 — 603.54 | 160.36 — 424.82 | 212.28 — 410.88 |
| Mean velocity in top | Mean ± SEM | 7.33 ± 0.24 | 7.16 ± 0.23 | 2.1 ± 0.24 | 2.7 ± 0.93 | 1.68 ± 0.33 | 4.84 ± 3.32 | 5.12 ± 0.34 | 5.6 ± 0.64 | 10.12 ± 0.42 | 8.38 ± 1.09 | 10.54 ± 0.59 | 8.38 ± 0.61 | 3.13 ± 0.21 | 2.5 ± 0.16 | 6.04 ± 0.63 | 6.94 ± 0.45 | 10.07 ± 0.89 | 8.27 ± 0.61 |
|  | Min — Max | 2.15 — 14.6 | 1.82 — 14.15 | 1.11 — 3.47 | 0.93 — 8.05 | 1 — 2.28 | 0.99 — 18.09 | 3.72 — 6.77 | 3.27 — 7.82 | 8.09 — 12.09 | 4.37 — 13.27 | 7.46 — 12.95 | 5.07 — 11.33 | 1.84 — 3.68 | 1.94 — 3.23 | 3.57 — 9.12 | 5.18 — 9.33 | 6.92 — 14.09 | 6.43 — 10.44 |
| Distance (cm) traveled in bottom | Mean ± SEM | 3546.23 ± 159.86 | 3344.49 ± 155.67 | 405.3 ± 57.67 | 500.53 ± 161.03 | 487.83 ± 130.38 | 529.52 ± 217.24 | 2821.06 ± 160.08 | 2940.26 ± 371.5 | 5023.94 ± 399.01 | 4019.55 ± 687.16 | 5004.77 ± 422.45 | 3869.34 ± 429.05 | 1474.95 ± 189.59 | 1204.84 ± 133.35 | 3010.14 ± 444.54 | 3532.12 ± 430.8 | 4645.8 ± 537.06 | 3616.56 ± 350.39 |
|  | Min — Max | 32.65 — 7653.95 | 24.97 — 7247.97 | 216.41 — 669.63 | 132.22 — 1282.91 | 180.02 — 812.91 | 121.17 — 1320.09 | 1929.38 — 3359.99 | 1346.87 — 4150.31 | 3614.24 — 6696.48 | 1482.11 — 7786.73 | 2721.97 — 6187.5 | 1697.76 — 5865.59 | 885.1 — 2544.32 | 630.43 — 1713.44 | 1344.98 — 5093.78 | 1871.5 — 5807.27 | 3142.39 — 7663.62 | 1917.24 — 4928.22 |
| Distance (cm) traveled in bottom first 4min | Mean ± SEM | 937.03 ± 46.51 | 824.04 ± 45.98 | 379.69 ± 55 | 349 ± 73.37 | 433.53 ± 137.07 | 220.72 ± 61.95 | 923.14 ± 97.72 | 803.12 ± 113.42 | 1186.96 ± 79.92 | 993.01 ± 159.37 | 1419.72 ± 143.87 | 1046.92 ± 166.96 | 589.37 ± 94.7 | 399.66 ± 73.58 | 781.78 ± 150.46 | 827.4 ± 106.67 | 1206.4 ± 167.67 | 948.49 ± 118.66 |
|  | Min — Max | 4.89 — 2327.23 | 7.6 — 2450.93 | 181.16 — 640.2 | 132.22 — 591.19 | 117.89 — 786.72 | 54.12 — 389.56 | 562.12 — 1309.41 | 317.4 — 1243.81 | 789.91 — 1448.6 | 422.58 — 1760.3 | 906.07 — 2093.8 | 540.88 — 2026.62 | 347.47 — 1208.72 | 192.03 — 818.1 | 261.42 — 1360.89 | 472.14 — 1182.27 | 621.8 — 1980.49 | 605.71 — 1432.79 |
| % time in bottom | Mean ± SEM | 30.11 ± 1.44 | 32.76 ± 1.59 | 3.91 ± 1.11 | 16.89 ± 9.79 | 3.6 ± 0.59 | 17.44 ± 12.67 | 44.52 ± 2.61 | 37.23 ± 3.77 | 47.79 ± 3.57 | 44.95 ± 4.9 | 42.59 ± 2.83 | 46.26 ± 4.94 | 32.99 ± 4.58 | 30.15 ± 5.87 | 37.61 ± 4.58 | 41.52 ± 5.09 | 41.38 ± 2.69 | 42.85 ± 4.52 |
|  | Min — Max | 0.04 — 66.3 | 0.02 — 78.19 | 1.27 — 11.04 | 1.91 — 70.68 | 2.47 — 5.14 | 1.25 — 67.06 | 34.27 — 54.85 | 19.49 — 49.14 | 30.37 — 56.89 | 26.19 — 68.16 | 30.37 — 57.92 | 20.83 — 63.37 | 17.07 — 50.78 | 11.62 — 57.08 | 15.19 — 54.07 | 22.2 — 61.18 | 31.02 — 52.07 | 23.19 — 57.62 |
| % time in top | Mean ± SEM | 69.62 ± 1.45 | 67.03 ± 1.59 | 77.39 ± 9.45 | 71.18 ± 11.09 | 86.92 ± 6.33 | 80.85 ± 12.96 | 50.98 ± 2.73 | 56.27 ± 3.71 | 36.85 ± 1.82 | 41.63 ± 3.01 | 49.96 ± 2.19 | 47.91 ± 5.59 | 59.96 ± 5.1 | 64.04 ± 5.22 | 53.02 ± 4.23 | 46.14 ± 4.56 | 44.41 ± 3.29 | 41.08 ± 2.62 |
|  | Min — Max | 33.61 — 99.96 | 21.02 — 99.98 | 22.8 — 97.76 | 19.71 — 95.57 | 68.96 — 97.45 | 29.94 — 98.7 | 41.01 — 60.81 | 46.21 — 76.32 | 25.84 — 41.35 | 29.62 — 55.59 | 38.99 — 56.67 | 33.11 — 75.9 | 42.23 — 82.42 | 42.15 — 82.54 | 34.16 — 70.85 | 30.07 — 69.5 | 27.19 — 58.12 | 31.18 — 52.5 |
| % time in neutral | Mean ± SEM | 0.27 ± 0.04 | 0.21 ± 0.05 | 18.7 ± 9.27 | 11.93 ± 7.14 | 9.48 ± 5.76 | 1.71 ± 0.96 | 4.5 ± 0.98 | 6.51 ± 1.49 | 15.36 ± 5.11 | 13.42 ± 4.98 | 7.45 ± 1.97 | 5.84 ± 1.9 | 7.05 ± 2.49 | 5.81 ± 2.15 | 9.37 ± 3.05 | 12.34 ± 5.08 | 14.21 ± 4.1 | 16.07 ± 6.04 |
|  | Min — Max | 0 — 2.59 | 0 — 5.08 | 0.28 — 75.93 | 0.29 — 54.2 | 0.08 — 25.89 | 0.06 — 4.87 | 2.24 — 10.82 | 4.09 — 14.54 | 3.76 — 43.79 | 2.22 — 38.61 | 3.09 — 17.75 | 1.75 — 16.63 | 0.51 — 17.92 | 0.76 — 18.21 | 0.82 — 26.99 | 1.94 — 39.34 | 5.4 — 41.79 | 2.95 — 45.63 |
| Entries into bottom | Mean ± SEM | 64.25 ± 3.17 | 56.28 ± 3.07 | 12.75 ± 2.02 | 10.14 ± 2.19 | 24 ± 3.03 | 10.4 ± 5.26 | 71.25 ± 5.05 | 74 ± 7.16 | 157.5 ± 18.44 | 116.5 ± 12.41 | 135.25 ± 14.21 | 105.38 ± 15.39 | 49 ± 11.01 | 41.5 ± 9.62 | 82.75 ± 10.6 | 126.75 ± 19.97 | 128.75 ± 18.37 | 125.25 ± 23.41 |
|  | Min — Max | 2 — 153 | 1 — 187 | 7 — 26 | 1 — 16 | 16 — 30 | 2 — 31 | 51 — 96 | 50 — 101 | 102 — 240 | 78 — 177 | 75 — 215 | 47 — 172 | 8 — 95 | 17 — 91 | 46 — 113 | 60 — 239 | 79 — 223 | 70 — 269 |
| Duration (s) immobile in bottom | Mean ± SEM | 246.59 ± 12.77 | 289.65 ± 15.2 | 27.3 ± 8.56 | 176.32 ± 114.25 | 28.78 ± 7.37 | 185.68 ± 143.38 | 338.17 ± 34.98 | 246.75 ± 35.01 | 240.57 ± 29.36 | 275.48 ± 39.35 | 209.28 ± 20.09 | 303.6 ± 38.09 | 310.93 ± 49.58 | 305.14 ± 69.45 | 248.42 ± 30.65 | 278.83 ± 37.59 | 230.78 ± 19.91 | 262.93 ± 34.35 |
|  | Min — Max | 0 — 630.96 | 0 — 782.88 | 4.74 — 80.88 | 0 — 801.87 | 13.41 — 48.52 | 1.87 — 745.75 | 186.72 — 455.46 | 132.07 — 387.45 | 131.06 — 368.77 | 134.8 — 459.73 | 150.55 — 333.13 | 133.13 — 439.51 | 162.83 — 545.15 | 85.42 — 628.5 | 81.35 — 359.63 | 144.41 — 432.7 | 171.71 — 318.18 | 126.99 — 384.85 |
| Mean velocity in bottom | Mean ± SEM | 14.04 ± 1.15 | 12.92 ± 1.15 | 12.97 ± 2.87 | 23.5 ± 15.61 | 36.91 ± 25.55 | 20.48 ± 9.4 | 5.73 ± 0.43 | 7.16 ± 0.5 | 10.24 ± 0.8 | 8.41 ± 0.97 | 10.35 ± 0.7 | 7.92 ± 0.85 | 4.76 ± 0.27 | 4.58 ± 0.44 | 7.11 ± 0.4 | 8.37 ± 0.53 | 10.43 ± 0.85 | 8.75 ± 0.86 |
|  | Min — Max | 6.4 — 148.58 | 4.56 — 124.74 | 5.57 — 26.93 | 1.96 — 116.54 | 3.84 — 112.29 | 1.77 — 47.73 | 4.15 — 7.88 | 6.21 — 9.36 | 7.55 — 13.28 | 5.06 — 14.19 | 7.59 — 12.61 | 6.04 — 13.24 | 3.43 — 5.66 | 2.67 — 5.99 | 5.81 — 8.91 | 6.03 — 10.75 | 8.16 — 14.29 | 5.68 — 13.94 |
| Distance (cm) traveled in bottom slope | Mean ± SEM | -0.25 ± 0.06 | -0.2 ± 0.05 | -2.45 ± 0.15 | -2.06 ± 0.28 | -0.83 ± 0.45 | -1.14 ± 0.42 | -0.13 ± 0.02 | -0.09 ± 0.02 | -0.07 ± 0.02 | -0.05 ± 0.04 | -0.13 ± 0.04 | -0.08 ± 0.04 | -1.06 ± 0.42 | -0.11 ± 0.06 | -0.02 ± 0.06 | -0.01 ± 0.03 | -0.11 ± 0.04 | -0.16 ± 0.08 |
|  | Min — Max | -3.31 — 1.73 | -3.06 — 1.5 | -3.08 — -2.06 | -2.81 — -0.58 | -2.13 — -0.13 | -2.07 — -0.17 | -0.21 — -0.06 | -0.17 — -0.02 | -0.18 — 0 | -0.25 — 0.12 | -0.38 — -0.02 | -0.25 — 0.02 | -3.1 — -0.05 | -0.36 — 0.07 | -0.31 — 0.21 | -0.13 — 0.15 | -0.28 — 0 | -0.56 — 0.14 |
| Time (s) in bottom slope | Mean ± SEM | -0.07 ± 0.04 | 0 ± 0.05 | -1.89 ± 0.14 | -1.7 ± 0.18 | -0.62 ± 0.45 | -0.89 ± 0.42 | 0.02 ± 0.04 | 0.04 ± 0.04 | 0.01 ± 0.02 | 0.03 ± 0.03 | -0.08 ± 0.03 | -0.07 ± 0.04 | -0.92 ± 0.38 | 0.03 ± 0.08 | 0 ± 0.05 | 0.06 ± 0.05 | -0.04 ± 0.04 | -0.13 ± 0.07 |
|  | Min — Max | -2.57 — 1.85 | -2.36 — 1.67 | -2.52 — -1.47 | -2.21 — -0.8 | -1.68 — 0.16 | -2.08 — 0.07 | -0.13 — 0.23 | -0.1 — 0.21 | -0.05 — 0.1 | -0.05 — 0.22 | -0.23 — 0.02 | -0.25 — 0.17 | -2.62 — 0.01 | -0.29 — 0.33 | -0.28 — 0.15 | -0.11 — 0.26 | -0.12 — 0.17 | -0.45 — 0.03 |
| Distance in bottom to total arena (ratio) | Mean ± SEM | 0.37 ± 0.01 | 0.37 ± 0.01 | 0.17 ± 0.02 | 0.23 ± 0.07 | 0.22 ± 0.04 | 0.25 ± 0.09 | 0.45 ± 0.01 | 0.42 ± 0.02 | 0.47 ± 0.02 | 0.44 ± 0.04 | 0.41 ± 0.02 | 0.43 ± 0.03 | 0.4 ± 0.04 | 0.39 ± 0.04 | 0.41 ± 0.04 | 0.44 ± 0.04 | 0.41 ± 0.02 | 0.4 ± 0.03 |
|  | Min — Max | 0.01 — 0.66 | 0.01 — 0.63 | 0.1 — 0.29 | 0.07 — 0.52 | 0.1 — 0.26 | 0.07 — 0.58 | 0.38 — 0.5 | 0.31 — 0.51 | 0.37 — 0.53 | 0.3 — 0.61 | 0.33 — 0.51 | 0.26 — 0.55 | 0.29 — 0.56 | 0.22 — 0.55 | 0.22 — 0.54 | 0.29 — 0.6 | 0.34 — 0.47 | 0.3 — 0.51 |
|  |  |  |  |  |  |  |  |  |  |  |  |  |  |  |  |  |  |  |  |
