# Supplementary material for: High-precision genetic mapping of behavioral traits in the diversity outbred mouse population
Source: Genes Brain Behav. 2013 Mar 20;12(4):424–37. doi: 10.1111/gbb.12029 (PMC3709837; doi:10.1111/gbb.12029)
Supplement: Supplementary file 4 [file gbb0012-0424-SD4.doc]

**Supplemental table 4:** Summary statistics of behaviors during tail-suspension test of progenitor and DO mice.

|  |  |  |  | **Strain** | | | | | | | | | | | | | | | |
| --- | --- | --- | --- | --- | --- | --- | --- | --- | --- | --- | --- | --- | --- | --- | --- | --- | --- | --- | --- |
|  |  | **Diversity Outbred** | | **129S1/SvImJ** | | **A/J** | | **C57BL/6J** | | **Cast/EiJ** | | **NOD/ShiLtJ** | | **NZO/H1LTJ** | | **PWK/PhJ** | | **WSB/EiJ** | |
| **Trait** | **Statistics** | **Female** | **Male** | **Female** | **Male** | **Female** | **Male** | **Female** | **Male** | **Female** | **Male** | **Female** | **Male** | **Female** | **Male** | **Female** | **Male** | **Female** | **Male** |
| **Tail suspension test** | N | 129 | 129 | 7 | 8 | 8 | 8 | 8 | 8 | 8 | 4 | 8 | 8 | 8 | 8 | 7 | 7 | 8 | 8 |
| Climbing frequency | Mean ± SEM | 1.58 ± 0.5 | 0.56 ± 0.22 | 0 ± 0 | 0 ± 0 | 0 ± 0 | 0 ± 0 | 0 ± 0 | 0.13 ± 0.13 | 6 ± 0.53 | 2.25 ± 1.65 | 0 ± 0 | 0 ± 0 | 0 ± 0 | 0 ± 0 | 13 ± 1.79 | 11 ± 0.72 | 14.75 ± 1.5 | 4.38 ± 1.4 |
|  | Min — Max | 0 — 59 | 0 — 25 | 0 — 0 | 0 — 0 | 0 — 0 | 0 — 0 | 0 — 0 | 0 — 1 | 4 — 8 | 0 — 7 | 0 — 0 | 0 — 0 | 0 — 0 | 0 — 0 | 5 — 20 | 8 — 14 | 9 — 23 | 0 — 11 |
| Duration (s) immobile | Mean ± SEM | 115.39 ± 3.75 | 115.94 ± 4.29 | 147.43 ± 21.46 | 116.44 ± 21.12 | 137.5 ± 21.51 | 149.9 ± 21.48 | 189.71 ± 6.44 | 172.4 ± 17.27 | 144.41 ± 33.69 | 150.28 ± 37.16 | 124.57 ± 23.66 | 143 ± 16.76 | 108.42 ± 28.08 | 57.96 ± 17.39 | 127.87 ± 34.59 | 138.86 ± 34.13 | 166.77 ± 39.57 | 132.04 ± 34.85 |
|  | Min — Max | 31.36 — 220.49 | 12.88 — 238.64 | 33.57 — 197.4 | 28.43 — 199.47 | 61.19 — 236.9 | 70.54 — 239.91 | 162.3 — 212.35 | 69.54 — 220.69 | 30.23 — 291.02 | 50.65 — 222.69 | 28.9 — 215.68 | 102.77 — 254.39 | 16.08 — 205.94 | 3.27 — 155.49 | 50.38 — 258.66 | 47.45 — 285.29 | 24.89 — 287.55 | 2.14 — 279.08 |
| Frequency of immobility | Mean ± SEM | 373.7 ± 9.33 | 390.12 ± 8.79 | 410.57 ± 42.8 | 354.38 ± 25.65 | 405.25 ± 25.96 | 389.25 ± 24.47 | 270.63 ± 18.13 | 289.5 ± 34.83 | 298 ± 63.71 | 334.75 ± 32.67 | 370.63 ± 17.01 | 321.75 ± 28 | 345.63 ± 42.69 | 257.88 ± 34.15 | 236.71 ± 41.53 | 324.29 ± 66.29 | 161.5 ± 32.16 | 243.13 ± 50.44 |
|  | Min — Max | 179 — 725 | 154 — 685 | 221 — 579 | 264 — 436 | 313 — 533 | 305 — 500 | 209 — 336 | 170 — 460 | 26 — 479 | 256 — 389 | 306 — 461 | 172 — 441 | 143 — 457 | 49 — 379 | 103 — 389 | 50 — 533 | 51 — 262 | 31 — 403 |
| Latency to first immobile | Mean ± SEM | 3.77 ± 0.44 | 3.06 ± 0.39 | 2.12 ± 0.76 | 8.57 ± 4.68 | 1.48 ± 0.75 | 1.52 ± 1.27 | 1.91 ± 1.11 | 2.36 ± 0.54 | 0.34 ± 0.11 | 0.18 ± 0.1 | 0.95 ± 0.42 | 4.26 ± 1.45 | 0.58 ± 0.19 | 3.62 ± 1.86 | 0.62 ± 0.52 | 1.56 ± 1.17 | 0.63 ± 0.35 | 4.22 ± 4 |
|  | Min — Max | 0.07 — 26.56 | 0.07 — 23.49 | 0.13 — 5.21 | 0.33 — 40.24 | 0.07 — 6.47 | 0.07 — 10.41 | 0.07 — 9.34 | 0.2 — 4.8 | 0.07 — 0.93 | 0.07 — 0.47 | 0.2 — 3.8 | 0.2 — 11.81 | 0.07 — 1.6 | 0.07 — 15.35 | 0.07 — 3.74 | 0.07 — 8.48 | 0.07 — 2.54 | 0.07 — 32.23 |
|  |  |  |  |  |  |  |  |  |  |  |  |  |  |  |  |  |  |  |  |
