# Supplementary material for: High-precision genetic mapping of behavioral traits in the diversity outbred mouse population
Source: Genes Brain Behav. 2013 Mar 20;12(4):424–37. doi: 10.1111/gbb.12029 (PMC3709837; doi:10.1111/gbb.12029)
Supplement: Supplementary file 5 [file gbb0012-0424-SD5.doc]

**Supplemental table 5:** Genes within QTL interval on chromosome 4 for center time slope.

| Chr | cM | start | end | strand NCBI Build 37 | MGI ID | Feature Type | Symbol | Name |
| --- | --- | --- | --- | --- | --- | --- | --- | --- |
| 4 | 79.08 | 148721994 | 148739386 | + | MGI:1925072 | lincRNA gene | A930028C08Rik | RIKEN cDNA A930028C08 gene |
| 4 | 78.76 | 147869292 | 147874569 | - | MGI:3605801 | protein coding gene | Angptl7 | angiopoietin-like 7 |
| 4 | 78.89 | 148501230 | 148511738 | - | MGI:1917178 | protein coding gene | Apitd1 | apoptosis-inducing, TAF9-like domain 1 |
| 4 | 78.87 | 148178538 | 148328998 | + | MGI:1196251 | protein coding gene | Casz1 | castor homolog 1, zinc finger (Drosophila) |
| 4 | 79.91 | 148960577 | 149023008 | + | MGI:1929895 | protein coding gene | Clstn1 | calsyntenin 1 |
| 4 | 78.89 | 148499143 | 148500872 | - | MGI:109538 | protein coding gene | Cort | cortistatin |
| 4 | 79.66 | 148892345 | 148940546 | + | MGI:1915756 | protein coding gene | Ctnnbip1 | catenin beta interacting protein 1 |
| 4 | 78.87 | 148478255 | 148494756 | + | MGI:1196227 | protein coding gene | Dffa | DNA fragmentation factor, alpha subunit |
| 4 | 78.76 | 147932538 | 147956510 | + | MGI:1355322 | protein coding gene | Exosc10 | exosome component 10 |
| 4 | 78.82 | 148017426 | 148045681 | + | MGI:2685418 | protein coding gene | Gm572 | predicted gene 572 |
| 4 | 79.05 | 148550428 | 148681802 | - | MGI:108426 | protein coding gene | Kif1b | kinesin family member 1B |
| 4 | 79.53 | 148859338 | 148870777 | + | MGI:1916401 | protein coding gene | Lzic | leucine zipper and CTNNBIP1 domain containing |
| 4 | 78.76 | 147976663 | 147989608 | + | MGI:1330832 | protein coding gene | Masp2 | mannan-binding lectin serine peptidase 2 |
| 4 | 78.76 | 147822720 | 147931792 | + | MGI:1928394 | protein coding gene | Mtor | mechanistic target of rapamycin (serine/threonine kinase) |
| 4 | 79.47 | 148841681 | 148859311 | - | MGI:1913704 | protein coding gene | Nmnat1 | nicotinamide nucleotide adenylyltransferase 1 |
| 4 | 78.87 | 148334644 | 148473985 | - | MGI:1927868 | protein coding gene | Pex14 | peroxisomal biogenesis factor 14 |
| 4 | 78.96 | 148524100 | 148540880 | - | MGI:97553 | protein coding gene | Pgd | phosphogluconate dehydrogenase |
| 4 | 80.15 | 149023277 | 149076680 | - | MGI:1098211 | protein coding gene | Pik3cd | phosphatidylinositol 3-kinase catalytic delta polypeptide |
| 4 | 79.4 | 148823796 | 148829087 | - | MGI:1890409 | protein coding gene | Rbp7 | retinol binding protein 7, cellular |
| 4 | 80.15 | 149118145 | 149148386 | - | MGI:1917806 | protein coding gene | Slc25a33 | solute carrier family 25, member 33 |
| 4 | 80.46 | 149270392 | 149329152 | - | MGI:1921896 | protein coding gene | Spsb1 | splA/ryanodine receptor domain and SOCS box containing 1 |
| 4 | 78.76 | 147965612 | 147969102 | + | MGI:102690 | protein coding gene | Srm | spermidine synthase |
| 4 | 78.77 | 147986491 | 148001128 | - | MGI:2387629 | protein coding gene | Tardbp | TAR DNA binding protein |
| 4 | 80.15 | 149089484 | 149112153 | - | MGI:1196277 | protein coding gene | Tmem201 | transmembrane protein 201 |
| 4 | 79.08 | 148702525 | 148800858 | - | MGI:1927086 | protein coding gene | Ube4b | ubiquitination factor E4B, UFD2 homolog (S. cerevisiae) |
| 4 | 78.76 | 147808604 | 147818880 | - | MGI:1918957 | protein coding gene | Ubiad1 | UbiA prenyltransferase domain containing 1 |
| 4 | 80.27 | 149188775 | 149189331 | - | MGI:3651735 | pseudogene | Gm13064 | predicted gene 13064 |
| 4 | 80.22 | 149167388 | 149167528 | - | MGI:3651736 | pseudogene | Gm13065 | predicted gene 13065 |
| 4 | 79.59 | 148874742 | 148875117 | + | MGI:3651971 | pseudogene | Gm13071 | predicted gene 13071 |
| 4 | 79.43 | 148831438 | 148831815 | + | MGI:3651720 | pseudogene | Gm13072 | predicted gene 13072 |
| 4 | 78.76 | 147943165 | 147943985 | - | MGI:3651444 | pseudogene | Gm13204 | predicted gene 13204 |
| 4 | 78.87 | 148470492 | 148470811 | + | MGI:3779916 | pseudogene | Gm9506 | predicted gene 9506 |
| 4 | 78.94 | 148516606 | 148517497 | - | MGI:3651234 | pseudogene | Rpsa-ps12 | ribosomal protein SA, pseudogene 12 |
|  |  |  |  |  |  |  |  |  |
