# Supplementary material for: High-precision genetic mapping of behavioral traits in the diversity outbred mouse population
Source: Genes Brain Behav. 2013 Mar 20;12(4):424–37. doi: 10.1111/gbb.12029 (PMC3709837; doi:10.1111/gbb.12029)
Supplement: Supplementary file 6 [file gbb0012-0424-SD6.doc]

**Supplemental table 6:** Genes within QTL interval on chromosomes 2, 6 and 19 for percent time immobile.

| Chr | cM | start | end | strand NCBI Build 37 | MGI ID | Feature Type | Symbol | Name |  |  |  |  |  |  |
| --- | --- | --- | --- | --- | --- | --- | --- | --- | --- | --- | --- | --- | --- | --- |
| 2 | 51.83 | 94246308 | 94251839 | + | MGI:1913707 | lincRNA gene | 2810002D19Rik | RIKEN cDNA 2810002D19 gene |  |  |  |  |  |  |
| 2 | 51.62 | 93820191 | 93823732 | + | MGI:1918102 | lincRNA gene | 4921507L20Rik | RIKEN cDNA 4921507L20 gene |  |  |  |  |  |  |
| 2 | 51.62 | 94081521 | 94081610 | - | MGI:3618711 | miRNA gene | Mir129-2 | microRNA 129-2 |  |  |  |  |  |  |
| 2 | 51.64 | 94101457 | 94101556 | - | MGI:3629629 | miRNA gene | Mir670 | microRNA 670 |  |  |  |  |  |  |
| 2 | 51.62 | 93673624 | 93690100 | - | MGI:1919717 | protein coding gene | Accs | 1-aminocyclopropane-1-carboxylate synthase homolog (Arabidopsis)(non-functional) |  |  |  |  |  |  |
| 2 | 51.62 | 93695518 | 93709314 | - | MGI:3584519 | protein coding gene | Accsl | 1-aminocyclopropane-1-carboxylate synthase homolog (Arabidopsis)(non-functional)-like |  |  |  |  |  |  |
| 2 | 51.62 | 93820707 | 93850964 | - | MGI:1916363 | protein coding gene | Alkbh3 | alkB, alkylation repair homolog 3 (E. coli) |  |  |  |  |  |  |
| 2 | 51.62 | 93482541 | 93521496 | + | MGI:108359 | protein coding gene | Alx4 | aristaless-like homeobox 4 |  |  |  |  |  |  |
| 2 | 51.84 | 94251839 | 94278293 | - | MGI:1888993 | protein coding gene | Api5 | apoptosis inhibitor 5 |  |  |  |  |  |  |
| 2 | 51.62 | 93535788 | 93662725 | - | MGI:108050 | protein coding gene | Ext2 | exostoses (multiple) 2 |  |  |  |  |  |  |
| 2 | 53.07 | 98506704 | 98507458 | - | MGI:3641657 | protein coding gene | Gm10800 | predicted gene 10800 |  |  |  |  |  |  |
| 2 | 53.07 | 98502394 | 98504240 | + | MGI:3641656 | protein coding gene | Gm10801 | predicted gene 10801 |  |  |  |  |  |  |
| 2 | 51.62 | 93400725 | 93404518 | + | MGI:3641754 | protein coding gene | Gm10803 | predicted gene 10803 |  |  |  |  |  |  |
| 2 | 51.62 | 93872846 | 93998121 | - | MGI:1926967 | protein coding gene | Hsd17b12 | hydroxysteroid (17-beta) dehydrogenase 12 |  |  |  |  |  |  |
| 2 | 52.3 | 96158326 | 97471823 | + | MGI:2442636 | protein coding gene | Lrrc4c | leucine rich repeat containing 4C |  |  |  |  |  |  |
| 2 | 51.69 | 94140924 | 94246845 | - | MGI:1921819 | protein coding gene | Ttc17 | tetratricopeptide repeat domain 17 |  |  |  |  |  |  |
| 2 | 51.62 | 93780456 | 93781035 | - | MGI:2686181 | pseudogene | Gm1335 | predicted gene 1335 |  |  |  |  |  |  |
| 2 | 52.01 | 94430978 | 94431578 | - | MGI:3650569 | pseudogene | Gm13788 | predicted gene 13788 |  |  |  |  |  |  |
| 2 | 52.01 | 95164847 | 95165141 | - | MGI:3652246 | pseudogene | Gm13795 | predicted gene 13795 |  |  |  |  |  |  |
| 2 | 52.12 | 95515934 | 95516246 | - | MGI:3652040 | pseudogene | Gm13796 | predicted gene 13796 |  |  |  |  |  |  |
| 2 | 52.12 | 95538074 | 95538193 | - | MGI:3652035 | pseudogene | Gm13797 | predicted gene 13797 |  |  |  |  |  |  |
| 2 | 52.12 | 95517874 | 95518012 | + | MGI:3652036 | pseudogene | Gm13798 | predicted gene 13798 |  |  |  |  |  |  |
| 2 | 52.26 | 95911686 | 95911851 | + | MGI:3651792 | pseudogene | Gm13800 | predicted gene 13800 |  |  |  |  |  |  |
| 2 | 52.27 | 95973592 | 95973751 | + | MGI:3651791 | pseudogene | Gm13801 | predicted gene 13801 |  |  |  |  |  |  |
| 2 | 52.4 | 97789348 | 97789836 | - | MGI:3651056 | pseudogene | Gm13803 | predicted gene 13803 |  |  |  |  |  |  |
| 2 | 53.07 | 98131560 | 98131731 | + | MGI:3651562 | pseudogene | Gm13804 | predicted gene 13804 |  |  |  |  |  |  |
| 2 | 53.05 | 98022722 | 98022825 | - | MGI:3651563 | pseudogene | Gm13805 | predicted gene 13805 |  |  |  |  |  |  |
| 2 | 53.07 | 98441187 | 98441511 | + | MGI:3651556 | pseudogene | Gm13806 | predicted gene 13806 |  |  |  |  |  |  |
| 2 | 53.37 | 99899090 | 99899273 | + | MGI:3649745 | pseudogene | Gm13808 | predicted gene 13808 |  |  |  |  |  |  |
| 2 | 53.07 | 99395408 | 99396035 | + | MGI:3649440 | pseudogene | Gm13809 | predicted gene 13809 |  |  |  |  |  |  |
| 2 | 51.62 | 93634727 | 93635241 | + | MGI:3649322 | pseudogene | Gm13818 | predicted gene 13818 |  |  |  |  |  |  |
| 2 | 52.4 | 96839781 | 96840450 | + | MGI:4937902 | pseudogene | Gm17075 | predicted gene 17075 |  |  |  |  |  |  |
| 2 | 52.4 | 96880069 | 96881469 | + | MGI:4937903 | pseudogene | Gm17076 | predicted gene 17076 |  |  |  |  |  |  |
| 2 | 52.14 | 95647921 | 95650005 | + | MGI:5010589 | pseudogene | Gm18404 | predicted gene, 18404 |  |  |  |  |  |  |
| 2 | 52.12 | 95555611 | 95556178 | + | MGI:5010620 | pseudogene | Gm18435 | predicted gene, 18435 |  |  |  |  |  |  |
| 2 | 52.01 | 94515023 | 94515896 | + | MGI:5010953 | pseudogene | Gm18768 | predicted gene, 18768 |  |  |  |  |  |  |
| 2 | 51.8 | 94224659 | 94225255 | - | MGI:3613363 | pseudogene | Itpa-ps1 | inosine triphosphatase (nucleoside triphosphate pyrophosphatase) pseudogene 1 |  |  |  |  |  |  |
| 2 | 52.4 | 96827175 | 96923718 | - | MGI:1922130 | unclassified gene | 4930445B16Rik | RIKEN cDNA 4930445B16 gene |  |  |  |  |  |  |
| 2 | 51.63 | 94092036 | 94104915 | - | MGI:3041234 | unclassified gene | E530001K10Rik | RIKEN cDNA E530001K10 gene |  |  |  |  |  |  |
| 2 | 52.01 | 94569171 | 94570895 | - | MGI:3652243 | unclassified gene | Gm13793 | predicted gene 13793 |  |  |  |  |  |  |
| 2 | 52.02 | 95234399 | 95237808 | + | MGI:3652242 | unclassified gene | Gm13794 | predicted gene 13794 |  |  |  |  |  |  |
| 2 | 52.15 | 95678245 | 95680751 | + | MGI:3652037 | unclassified gene | Gm13799 | predicted gene 13799 |  |  |  |  |  |  |
| 2 | 51.62 | 93795967 | 93797684 | - | MGI:3652053 | unclassified gene | Gm13889 | predicted gene 13889 |  |  |  |  |  |  |
|  |  |  |  |  |  |  |  |  |  |  |  |  |  |  |
| 6 | 53.19 | 1.15E+08 | 1.15E+08 | - | MGI:4937367 | lincRNA gene | Gm17733 | predicted gene, 17733 |  |  |  |  |  |  |
| 6 | 53.14 | 1.15E+08 | 1.15E+08 | - | MGI:1916221 | protein coding gene | 1500001M20Rik | RIKEN cDNA 1500001M20 gene |  |  |  |  |  |  |
| 6 | 53.6 | 1.16E+08 | 1.16E+08 | - | MGI:1917541 | protein coding gene | 2510049J12Rik | RIKEN cDNA 2510049J12 gene |  |  |  |  |  |  |
| 6 | 53.05 | 1.15E+08 | 1.15E+08 | + | MGI:1921494 | protein coding gene | Atg7 | autophagy-related 7 (yeast) |  |  |  |  |  |  |
| 6 | 53.27 | 1.15E+08 | 1.15E+08 | + | MGI:4937116 | protein coding gene | Gm17482 | predicted gene, 17482 |  |  |  |  |  |  |
| 6 | 53.05 | 1.14E+08 | 1.14E+08 | + | MGI:107619 | protein coding gene | Hrh1 | histamine receptor H1 |  |  |  |  |  |  |
| 6 | 53.61 | 1.16E+08 | 1.16E+08 | + | MGI:1914277 | protein coding gene | Mkrn2 | makorin, ring finger protein, 2 |  |  |  |  |  |  |
| 6 | 53.41 | 1.15E+08 | 1.15E+08 | + | MGI:97747 | protein coding gene | Pparg | peroxisome proliferator activated receptor gamma |  |  |  |  |  |  |
| 6 | 53.62 | 1.16E+08 | 1.16E+08 | - | MGI:97847 | protein coding gene | Raf1 | v-raf-leukemia viral oncogene 1 |  |  |  |  |  |  |
| 6 | 53.2 | 1.15E+08 | 1.15E+08 | + | MGI:103020 | protein coding gene | Syn2 | synapsin II |  |  |  |  |  |  |
| 6 | 53.29 | 1.15E+08 | 1.15E+08 | - | MGI:109125 | protein coding gene | Timp4 | tissue inhibitor of metalloproteinase 4 |  |  |  |  |  |  |
| 6 | 53.58 | 1.15E+08 | 1.16E+08 | + | MGI:2141599 | protein coding gene | Tsen2 | tRNA splicing endonuclease 2 homolog (S. cerevisiae) |  |  |  |  |  |  |
| 6 | 53.07 | 1.15E+08 | 1.15E+08 | - | MGI:2652840 | protein coding gene | Vgll4 | vestigial like 4 (Drosophila) |  |  |  |  |  |  |
| 6 | 53.63 | 1.16E+08 | 1.16E+08 | - | MGI:3801741 | pseudogene | Gm14335 | predicted gene 14335 |  |  |  |  |  |  |
| 6 | 53.21 | 1.15E+08 | 1.15E+08 | - | MGI:5010510 | pseudogene | Gm18325 | predicted gene, 18325 |  |  |  |  |  |  |
| 6 | 53.05 | 1.15E+08 | 1.15E+08 | + | MGI:5011225 | pseudogene | Gm19040 | predicted gene, 19040 |  |  |  |  |  |  |
| 6 | 53.09 | 1.15E+08 | 1.15E+08 | - | MGI:1924838 | unclassified gene | 4631423B10Rik | RIKEN cDNA 4631423B10 gene |  |  |  |  |  |  |
|  |  |  |  |  |  |  |  |  |  |  |  |  |  |  |
| 19 | 10.91 | 16059580 | 16085402 | + | ENSMUSG00000087529 | MGI:3041192 | lincRNA gene | C130060C02Rik | RIKEN cDNA C130060C02 gene | | |  |  |  |
| 19 | 13.28 | 19005095 | 19185686 | - | ENSMUSG00000036192 | MGI:1343464 | protein coding gene | Rorb | RAR-related orphan receptor beta | | |  |  |  |
| 19 | 12.26 | 16946844 | 16948130 | - | ENSMUSG00000056829 | MGI:1347468 | protein coding gene | Foxb2 | forkhead box B2 | |  |  |  |  |
| 19 | 13.18 | 18745270 | 18779278 | + | ENSMUSG00000024726 | MGI:1914633 | protein coding gene | 2410127L17Rik | RIKEN cDNA 2410127L17 gene | | |  |  |  |
| 19 | 12.82 | 17468533 | 17475839 | + | ENSMUSG00000024712 | MGI:1914688 | protein coding gene | Rfk | riboflavin kinase | |  |  |  |  |
| 19 | 10.89 | 16030264 | 16059479 | - | ENSMUSG00000041491 | MGI:1924386 | protein coding gene | Cep78 | centrosomal protein 78 | |  |  |  |  |
| 19 | 12.34 | 17030608 | 17298422 | + | ENSMUSG00000039126 | MGI:1925004 | protein coding gene | Prune2 | prune homolog 2 (Drosophila) | | |  |  |  |
| 19 | 13.17 | 18706440 | 18726684 | + | ENSMUSG00000037847 | MGI:2147434 | protein coding gene | BC016495 | cDNA sequence BC016495 | | |  |  |  |
| 19 | 10.86 | 15979168 | 16021827 | - | ENSMUSG00000024640 | MGI:2183441 | protein coding gene | Psat1 | phosphoserine aminotransferase 1 | | |  |  |  |
| 19 | 11.71 | 16691253 | 16855417 | - | ENSMUSG00000046230 | MGI:2444304 | protein coding gene | Vps13a | vacuolar protein sorting 13A (yeast) | | |  |  |  |
| 19 | 13.21 | 18824473 | 18967000 | + | ENSMUSG00000024727 | MGI:2675603 | protein coding gene | Trpm6 | transient receptor potential cation channel, subfamily M, member 6 | | | | | |
| 19 | 13.2 | 18787726 | 18792918 | + | ENSMUSG00000047044 | MGI:3583960 | protein coding gene | D030056L22Rik | RIKEN cDNA D030056L22 gene | | |  |  |  |
| 19 | 13.17 | 18653818 | 18706279 | - | ENSMUSG00000024725 | MGI:700012 | protein coding gene | Ostf1 | osteoclast stimulating factor 1 | | |  |  |  |
| 19 | 12.75 | 17400631 | 17431157 | - | ENSMUSG00000038843 | MGI:95676 | protein coding gene | Gcnt1 | glucosaminyl (N-acetyl) transferase 1, core 2 | | | |  |  |
| 19 | 11.29 | 16510157 | 16685308 | + | ENSMUSG00000024697 | MGI:95769 | protein coding gene | Gna14 | guanine nucleotide binding protein, alpha 14 | | | |  |  |
| 19 | 11.01 | 16207321 | 16461953 | + | ENSMUSG00000024639 | MGI:95776 | protein coding gene | Gnaq | guanine nucleotide binding protein, alpha q polypeptide | | | | |  |
| 19 | 13.83 | 20447918 | 20465434 | - | ENSMUSG00000024659 | MGI:96819 | protein coding gene | Anxa1 | annexin A1 |  |  |  |  |  |
| 19 | 12.86 | 17507883 | 17912122 | - | ENSMUSG00000024713 | MGI:97515 | protein coding gene | Pcsk5 | proprotein convertase subtilisin/kexin type 5 | | | |  |  |
| 19 | 12.86 | 18001014 | 18001438 | + |  | MGI:2388571 | pseudogene | Eef1a1-ps1 | eukaryotic translation elongation factor 1 alpha 1, pseudogene 1 | | | | |  |
| 19 | 11.04 | 16238802 | 16239295 | - | ENSMUSG00000057990 | MGI:2442009 | pseudogene | E030024N20Rik | RIKEN cDNA E030024N20 gene | | |  |  |  |
| 19 | 11.12 | 16341551 | 16342111 | - | ENSMUSG00000075268 | MGI:3642135 | pseudogene | Gm10819 | predicted gene 10819 | |  |  |  |  |
| 19 | 11.38 | 16551597 | 16565612 | - |  | MGI:3643833 | pseudogene | Gm8222 | predicted gene 8222 | |  |  |  |  |
| 19 | 10.93 | 16099988 | 16100252 | + | ENSMUSG00000049247 | MGI:3646957 | pseudogene | Rpl37-ps1 | ribosomal protein 37, pseudogene 1 | | |  |  |  |
| 19 | 13.17 | 18655945 | 18656718 | + | ENSMUSG00000080783 | MGI:3647615 | pseudogene | Gm8250 | predicted gene 8250 | |  |  |  |  |
| 19 | 12.1 | 16855037 | 16855593 | + | ENSMUSG00000046388 | MGI:3708646 | pseudogene | Gm9806 | predicted gene 9806 | |  |  |  |  |
| 19 | 13.17 | 18639650 | 18640245 | + |  | MGI:3779480 | pseudogene | Rpl29-ps6 | ribosomal protein L29, pseudogene 6 | | |  |  |  |
| 19 | 10.88 | 16018100 | 16018655 | + | ENSMUSG00000090147 | MGI:3781507 | pseudogene | Gm3329 | predicted gene 3329 | |  |  |  |  |
| 19 | 11.13 | 16352287 | 16353266 | + | ENSMUSG00000078528 | MGI:4937895 | pseudogene | Gm17068 | predicted gene 17068 | |  |  |  |  |
| 19 | 12.86 | 17767266 | 17768024 | + |  | MGI:5010004 | pseudogene | Gm17819 | predicted gene, 17819 | |  |  |  |  |
| 19 | 13.17 | 18279656 | 18280285 | - |  | MGI:5010795 | pseudogene | Gm18610 | predicted gene, 18610 | |  |  |  |  |
| 19 | 11.25 | 16487984 | 16488482 | + |  | MGI:5011497 | pseudogene | Gm19312 | predicted gene, 19312 | |  |  |  |  |
| 19 | 12.81 | 17460493 | 17483388 | - |  | MGI:5011513 | unclassified gene | Gm19328 | predicted gene, 19328 | |  |  |  |  |
|  |  |  |  |  |  |  |  |  |  |  |  |  |  |  |
