# Supplementary material for: High-precision genetic mapping of behavioral traits in the diversity outbred mouse population
Source: Genes Brain Behav. 2013 Mar 20;12(4):424–37. doi: 10.1111/gbb.12029 (PMC3709837; doi:10.1111/gbb.12029)
Supplement: Supplementary file 7 [file gbb0012-0424-SD7.doc]

| Chr | cM | start | end | strand NCBI Build 37 | MGI ID | Feature Type | Symbol | Name |
| --- | --- | --- | --- | --- | --- | --- | --- | --- |
| 11 | 59.01 | 95720179 | 95722358 | + | MGI:1921156 | lincRNA gene | 4833417C18Rik | RIKEN cDNA 4833417C18 gene |
| 11 | 59.01 | 95006367 | 95020361 | + | MGI:2444057 | lincRNA gene | A730090H04Rik | RIKEN cDNA A730090H04 gene |
| 11 | 59.92 | 96309637 | 96325861 | - | MGI:3649352 | lincRNA gene | Gm11529 | predicted gene 11529 |
| 11 | 59.65 | 96064768 | 96066794 | + | MGI:3649911 | lincRNA gene | Gm11538 | predicted gene 11538 |
| 11 | 59.01 | 95531266 | 95534033 | + | MGI:4937003 | lincRNA gene | Gm17369 | predicted gene, 17369 |
| 11 | 59.83 | 96178479 | 96178588 | + | MGI:3619064 | miRNA gene | Mir10a | microRNA 10a |
| 11 | 59.8 | 96126478 | 96126579 | + | MGI:2676860 | miRNA gene | Mir196a-1 | microRNA 196a-1 |
| 11 | 59.08 | 95824606 | 95824697 | - | MGI:4834235 | miRNA gene | Mir3063 | microRNA 3063 |
| 11 | 59.01 | 95691388 | 95703790 | - | MGI:1913860 | protein coding gene | Abi3 | ABI gene family, member 3 |
| 11 | 59.33 | 95930166 | 95936984 | - | MGI:107653 | protein coding gene | Atp5g1 | ATP synthase, H+ transporting, mitochondrial F0 complex, subunit c1 (subunit 9) |
| 11 | 59.02 | 95727257 | 95776205 | - | MGI:1342058 | protein coding gene | B4galnt2 | beta-1,4-N-acetyl-galactosaminyl transferase 2 |
| 11 | 59.4 | 95960640 | 95973278 | - | MGI:1343177 | protein coding gene | Calcoco2 | calcium binding and coiled-coil domain 2 |
| 11 | 59.01 | 95198332 | 95243186 | + | MGI:2144564 | protein coding gene | Fam117a | family with sequence similarity 117, member A |
| 11 | 59.21 | 95885859 | 95892145 | + | MGI:107504 | protein coding gene | Gip | gastric inhibitory polypeptide |
| 11 | 59.76 | 96112419 | 96126182 | + | MGI:2684899 | protein coding gene | Gm53 | predicted gene 53 |
| 11 | 59.01 | 95558212 | 95560457 | - | MGI:3648412 | protein coding gene | Gm9796 | predicted gene 9796 |
| 11 | 59.01 | 95698530 | 95707048 | + | MGI:893584 | protein coding gene | Gngt2 | guanine nucleotide binding protein (G protein), gamma transducing activity polypeptide 2 |
| 11 | 59.86 | 96227066 | 96229570 | + | MGI:96182 | protein coding gene | Hoxb1 | homeobox B1 |
| 11 | 59.63 | 96055630 | 96058761 | + | MGI:107730 | protein coding gene | Hoxb13 | homeobox B13 |
| 11 | 59.85 | 96212946 | 96217003 | + | MGI:96183 | protein coding gene | Hoxb2 | homeobox B2 |
| 11 | 59.84 | 96184640 | 96207999 | + | MGI:96184 | protein coding gene | Hoxb3 | homeobox B3 |
| 11 | 59.83 | 96177998 | 96182952 | + | MGI:96185 | protein coding gene | Hoxb4 | homeobox B4 |
| 11 | 59.82 | 96163909 | 96167434 | + | MGI:96186 | protein coding gene | Hoxb5 | homeobox B5 |
| 11 | 59.82 | 96153790 | 96162883 | + | MGI:96187 | protein coding gene | Hoxb6 | homeobox B6 |
| 11 | 59.82 | 96145571 | 96151650 | + | MGI:96188 | protein coding gene | Hoxb7 | homeobox B7 |
| 11 | 59.82 | 96143219 | 96146639 | + | MGI:96189 | protein coding gene | Hoxb8 | homeobox B8 |
| 11 | 59.81 | 96132771 | 96137909 | + | MGI:96190 | protein coding gene | Hoxb9 | homeobox B9 |
| 11 | 59.08 | 95818477 | 95867254 | - | MGI:1890357 | protein coding gene | Igf2bp1 | insulin-like growth factor 2 mRNA binding protein 1 |
| 11 | 59.01 | 95135573 | 95171560 | - | MGI:2182799 | protein coding gene | Myst2 | MYST histone acetyltransferase 2 |
| 11 | 59.01 | 95430132 | 95449049 | - | MGI:97323 | protein coding gene | Ngfr | nerve growth factor receptor (TNFR superfamily, member 16) |
| 11 | 59.01 | 95371159 | 95375884 | - | MGI:1336188 | protein coding gene | Nxph3 | neurexophilin 3 |
| 11 | 59.01 | 95528271 | 95542087 | + | MGI:97572 | protein coding gene | Phb | prohibitin |
| 11 | 59.01 | 95685813 | 95693454 | + | MGI:2447348 | protein coding gene | Phospho1 | phosphatase, orphan 1 |
| 11 | 59.93 | 96325907 | 96620444 | + | MGI:1925723 | protein coding gene | Skap1 | src family associated phosphoprotein 1 |
| 11 | 59.01 | 95246006 | 95253090 | + | MGI:1343133 | protein coding gene | Slc35b1 | solute carrier family 35, member B1 |
| 11 | 59.24 | 95896199 | 95908744 | + | MGI:1343161 | protein coding gene | Snf8 | SNF8, ESCRT-II complex subunit, homolog (S. cerevisiae) |
| 11 | 59.01 | 95275394 | 95354720 | + | MGI:1343085 | protein coding gene | Spop | speckle-type POZ protein |
| 11 | 59.01 | 95122843 | 95130579 | + | MGI:1931130 | protein coding gene | Tac4 | tachykinin 4 |
| 11 | 59.48 | 95995100 | 96026765 | + | MGI:2683461 | protein coding gene | Ttll6 | tubulin tyrosine ligase-like family, member 6 |
| 11 | 59.28 | 95908746 | 95926702 | - | MGI:1343160 | protein coding gene | Ube2z | ubiquitin-conjugating enzyme E2Z (putative) |
| 11 | 59.01 | 95573987 | 95696429 | + | MGI:2442221 | protein coding gene | Zfp652 | zinc finger protein 652 |
| 11 | 59.01 | 95038127 | 95038558 | + | MGI:3649789 | pseudogene | Gm11514 | predicted gene 11514 |
| 11 | 59.01 | 95323860 | 95324035 | - | MGI:3650746 | pseudogene | Gm11522 | predicted gene 11522 |
| 11 | 59.01 | 95622805 | 95622979 | + | MGI:3650073 | pseudogene | Gm11526 | predicted gene 11526 |
| 11 | 59.88 | 96253426 | 96253980 | + | MGI:3650260 | pseudogene | Gm11531 | predicted gene 11531 |
| 11 | 59.76 | 96109947 | 96110503 | + | MGI:3651911 | pseudogene | Gm11539 | predicted gene 11539 |
| 11 | 59.82 | 96152338 | 96168224 | - | MGI:1922645 | unclassified gene | 0610040B09Rik | RIKEN cDNA 0610040B09 gene |
| 11 | 59.01 | 95561420 | 95574068 | - | MGI:3044900 | unclassified gene | 1110035M17Rik | RIKEN cDNA 1110035M17 gene |
| 11 | 59.01 | 95584901 | 95588024 | + | MGI:2444371 | unclassified gene | B130006D01Rik | RIKEN cDNA B130006D01 gene |
| 11 | 59.01 | 95084604 | 95086946 | - | MGI:3649766 | unclassified gene | Gm11515 | predicted gene 11515 |
| 11 | 59.01 | 95171743 | 95185810 | + | MGI:3650534 | unclassified gene | Gm11520 | predicted gene 11520 |
| 11 | 59.01 | 95197325 | 95198722 | - | MGI:3651772 | unclassified gene | Gm11521 | predicted gene 11521 |
| 11 | 59.01 | 95719476 | 95720488 | - | MGI:3649799 | unclassified gene | Gm11527 | predicted gene 11527 |
| 11 | 59.01 | 95483974 | 95491150 | + | MGI:3649788 | unclassified gene | Gm11528 | predicted gene 11528 |
| 11 | 59.02 | 95730468 | 95737694 | + | MGI:3650775 | unclassified gene | Gm11534 | predicted gene 11534 |
| 11 | 59.59 | 96038865 | 96040114 | + | MGI:3650113 | unclassified gene | Gm11535 | predicted gene 11535 |
| 11 | 59.84 | 96197613 | 96203422 | - | MGI:3650116 | unclassified gene | Gm11536 | predicted gene 11536 |
| 11 | 59.85 | 96206795 | 96216005 | - | MGI:103211 | unclassified gene | Hoxb3os | homeobox B3, opposite strand transcript |
|  |  |  |  |  |  |  |  |  |

**Supplemental table 7:** Genes within QTL interval on chromosome 11 for light time slope.
