# Supplementary material for: High-precision genetic mapping of behavioral traits in the diversity outbred mouse population
Source: Genes Brain Behav. 2013 Mar 20;12(4):424–37. doi: 10.1111/gbb.12029 (PMC3709837; doi:10.1111/gbb.12029)
Supplement: Supplementary file 8 [file gbb0012-0424-SD8.doc]

**Supplemental table 8:** Genes within QTL interval on chromosome 8 for percent light time.

| Chr | cM | start | end | strand NCBI Build 37 | Ensembl Gene ID | MGI ID | MGI ID | Feature Type | Symbol | Name |
| --- | --- | --- | --- | --- | --- | --- | --- | --- | --- | --- |
| 8 | 53.1 | 108659300 | 108662425 | + | ENSMUSG00000086390 | MGI:1917021 | MGI:1917021 | lincRNA gene | 1810019D21Rik | RIKEN cDNA 1810019D21 gene |
| 8 | 54.33 | 110075144 | 110075213 | + | ENSMUSG00000065439 | MGI:2676825 | MGI:2676825 | miRNA gene | Mir140 | microRNA 140 |
| 8 | 53.04 | 107832264 | 107832360 | - | ENSMUSG00000070130 | MGI:3619340 | MGI:3619340 | miRNA gene | Mir328 | microRNA 328 |
| 8 | 53.04 | 108139366 | 108139473 | + | ENSMUSG00000067626 | MGI:4936879 | MGI:3837211 | miRNA gene | Mir1966 | microRNA 1966 |
| 8 | 53.38 | 109459650 | 109538092 | + | ENSMUSG00000041308 | MGI:101771 | MGI:101771 | protein coding gene | Sntb2 | syntrophin, basic 2 |
| 8 | 53.04 | 107572499 | 107582314 | + | ENSMUSG00000069922 | MGI:102773 | MGI:102773 | protein coding gene | Ces3a | carboxylesterase 3A |
| 8 | 53.04 | 107821563 | 107829269 | + | ENSMUSG00000014859 | MGI:103012 | MGI:103012 | protein coding gene | E2f4 | E2F transcription factor 4 |
| 8 | 54.08 | 109912125 | 109927106 | - | ENSMUSG00000003849 | MGI:103187 | MGI:103187 | protein coding gene | Nqo1 | NAD(P)H dehydrogenase, quinone 1 |
| 8 | 53.08 | 108583503 | 108654437 | + | ENSMUSG00000031902 | MGI:103296 | MGI:103296 | protein coding gene | Nfatc3 | nuclear factor of activated T-cells, cytoplasmic, calcineurin-dependent 3 |
| 8 | 53.04 | 108042646 | 108047884 | + | ENSMUSG00000031891 | MGI:104720 | MGI:104720 | protein coding gene | Hsd11b2 | hydroxysteroid 11-beta dehydrogenase 2 |
| 8 | 53.14 | 108939239 | 108949628 | + | ENSMUSG00000031907 | MGI:104786 | MGI:104786 | protein coding gene | Zfp90 | zinc finger protein 90 |
| 8 | 53.04 | 107782186 | 107788509 | - | ENSMUSG00000031887 | MGI:109200 | MGI:109200 | protein coding gene | Tradd | TNFRSF1A-associated via death domain |
| 8 | 53.04 | 108160468 | 108206822 | + | ENSMUSG00000005698 | MGI:109447 | MGI:109447 | protein coding gene | Ctcf | CCCTC-binding factor |
| 8 | 53.28 | 109394142 | 109406802 | + | ENSMUSG00000031910 | MGI:109599 | MGI:109599 | protein coding gene | Has3 | hyaluronan synthase 3 |
| 8 | 53.06 | 108459628 | 108462292 | - | ENSMUSG00000031897 | MGI:1096380 | MGI:1096380 | protein coding gene | Psmb10 | proteasome (prosome, macropain) subunit, beta type 10 |
| 8 | 53.32 | 109417540 | 109446993 | + | ENSMUSG00000041438 | MGI:1096573 | MGI:1096573 | protein coding gene | Cirh1a | cirrhosis, autosomal recessive 1A (human) |
| 8 | 53.59 | 109593300 | 109620447 | - | ENSMUSG00000031921 | MGI:1195972 | MGI:1195972 | protein coding gene | Terf2 | telomeric repeat binding factor 2 |
| 8 | 53.04 | 108048365 | 108089947 | - | ENSMUSG00000013160 | MGI:1201778 | MGI:1201778 | protein coding gene | Atp6v0d1 | ATPase, H+ transporting, lysosomal V0 subunit D1 |
| 8 | 53.06 | 108467490 | 108489974 | - | ENSMUSG00000017765 | MGI:1309465 | MGI:1309465 | protein coding gene | Slc12a4 | solute carrier family 12, member 4 |
| 8 | 53.04 | 107793701 | 107799745 | + | ENSMUSG00000033249 | MGI:1347058 | MGI:1347058 | protein coding gene | Hsf4 | heat shock transcription factor 4 |
| 8 | 54.38 | 110104281 | 110112382 | - | ENSMUSG00000039067 | MGI:1351511 | MGI:1351511 | protein coding gene | Psmd7 | proteasome (prosome, macropain) 26S subunit, non-ATPase, 7 |
| 8 | 53.04 | 107788548 | 107793226 | + | ENSMUSG00000033313 | MGI:1354697 | MGI:1354697 | protein coding gene | Fbxl8 | F-box and leucine-rich repeat protein 8 |
| 8 | 53.93 | 109817370 | 109903417 | + | ENSMUSG00000003847 | MGI:1859333 | MGI:1859333 | protein coding gene | Nfat5 | nuclear factor of activated T-cells 5 |
| 8 | 53.53 | 109555122 | 109569655 | + | ENSMUSG00000031913 | MGI:1890520 | MGI:1890520 | protein coding gene | Vps4a | vacuolar protein sorting 4a (yeast) |
| 8 | 53.57 | 109580777 | 109584826 | + | ENSMUSG00000031917 | MGI:1913414 | MGI:1913414 | protein coding gene | Nip7 | nuclear import 7 homolog (S. cerevisiae) |
| 8 | 53.58 | 109585384 | 109589544 | - | ENSMUSG00000031919 | MGI:1913519 | MGI:1913519 | protein coding gene | Tmed6 | transmembrane emp24 protein transport domain containing 6 |
| 8 | 53.04 | 107850262 | 107859119 | + | ENSMUSG00000014856 | MGI:1913570 | MGI:1913570 | protein coding gene | Tmem208 | transmembrane protein 208 |
| 8 | 53.07 | 108515237 | 108577740 | + | ENSMUSG00000031901 | MGI:1913619 | MGI:1913619 | protein coding gene | Dus2l | dihydrouridine synthase 2-like (SMM1, S. cerevisiae) |
| 8 | 53.71 | 109674561 | 109711370 | + | ENSMUSG00000031924 | MGI:1913677 | MGI:1913677 | protein coding gene | Cyb5b | cytochrome b5 type B |
| 8 | 54.15 | 109960298 | 110082494 | + | ENSMUSG00000031930 | MGI:1914144 | MGI:1914144 | protein coding gene | Wwp2 | WW domain containing E3 ubiquitin protein ligase 2 |
| 8 | 54.12 | 109936386 | 109948951 | - | ENSMUSG00000003848 | MGI:1914869 | MGI:1914869 | protein coding gene | Nob1 | NIN1/RPN12 binding protein 1 homolog (S. cerevisiae) |
| 8 | 53.04 | 107991393 | 107995322 | - | ENSMUSG00000014846 | MGI:1915221 | MGI:1915221 | protein coding gene | Tppp3 | tubulin polymerization-promoting protein family member 3 |
| 8 | 53.55 | 109568598 | 109571086 | - | ENSMUSG00000078931 | MGI:1915273 | MGI:1915273 | protein coding gene | Pdf | peptide deformylase (mitochondrial) |
| 8 | 53.05 | 108384534 | 108404302 | + | ENSMUSG00000008450 | MGI:1915301 | MGI:1915301 | protein coding gene | Nutf2 | nuclear transport factor 2 |
| 8 | 53.11 | 108724338 | 108734833 | - | ENSMUSG00000033106 | MGI:1916951 | MGI:1916951 | protein coding gene | Slc7a6os | solute carrier family 7, member 6 opposite strand |
| 8 | 53.04 | 108237754 | 108282564 | - | ENSMUSG00000013150 | MGI:1917825 | MGI:1917825 | protein coding gene | Gfod2 | glucose-fructose oxidoreductase domain containing 2 |
| 8 | 53.04 | 107996325 | 108008330 | - | ENSMUSG00000039199 | MGI:1918046 | MGI:1918046 | protein coding gene | Zdhhc1 | zinc finger, DHHC domain containing 1 |
| 8 | 53.04 | 108232203 | 108234068 | + | ENSMUSG00000013158 | MGI:1918296 | MGI:1918296 | protein coding gene | 4933405L10Rik | RIKEN cDNA 4933405L10 gene |
| 8 | 53.07 | 108497420 | 108503319 | - | ENSMUSG00000031898 | MGI:1919104 | MGI:1919104 | protein coding gene | Dpep3 | dipeptidase 3 |
| 8 | 53.08 | 108533521 | 108535782 | - | ENSMUSG00000045538 | MGI:1919236 | MGI:1919236 | protein coding gene | Ddx28 | DEAD (Asp-Glu-Ala-Asp) box polypeptide 28 |
| 8 | 53.04 | 108351644 | 108368576 | + | ENSMUSG00000031893 | MGI:1919486 | MGI:1919486 | protein coding gene | Tsnaxip1 | translin-associated factor X (Tsnax) interacting protein 1 |
| 8 | 53.04 | 108292208 | 108351250 | - | ENSMUSG00000037415 | MGI:1921584 | MGI:1921584 | protein coding gene | Ranbp10 | RAN binding protein 10 |
| 8 | 53.04 | 107804310 | 107813428 | - | ENSMUSG00000014837 | MGI:1921606 | MGI:1921606 | protein coding gene | 4931428F04Rik | RIKEN cDNA 4931428F04 gene |
| 8 | 53.04 | 108129129 | 108146094 | + | ENSMUSG00000038604 | MGI:1922937 | MGI:1922937 | protein coding gene | Fam65a | family with sequence similarity 65, member A |
| 8 | 53.1 | 108654219 | 108660874 | - | ENSMUSG00000084128 | MGI:1924661 | MGI:1924661 | protein coding gene | Esrp2 | epithelial splicing regulatory protein 2 |
| 8 | 53.67 | 109643010 | 109643792 | + | ENSMUSG00000040399 | MGI:1925821 | MGI:1925821 | protein coding gene | C630050I24Rik | RIKEN cDNA C630050I24 gene |
| 8 | 53.04 | 107800347 | 107805839 | + | ENSMUSG00000014776 | MGI:1925938 | MGI:1925938 | protein coding gene | Nol3 | nucleolar protein 3 (apoptosis repressor with CARD domain) |
| 8 | 53.04 | 108225054 | 108227393 | + | ENSMUSG00000005699 | MGI:1927223 | MGI:1927223 | protein coding gene | Pard6a | par-6 (partitioning defective 6,) homolog alpha (C. elegans) |
| 8 | 53.12 | 108779159 | 108789819 | - | ENSMUSG00000031906 | MGI:1927578 | MGI:1927578 | protein coding gene | Smpd3 | sphingomyelin phosphodiesterase 3, neutral |
| 8 | 53.05 | 108379003 | 108380850 | + | ENSMUSG00000036442 | MGI:1930964 | MGI:1930964 | protein coding gene | Thap11 | THAP domain containing 11 |
| 8 | 53.04 | 107897174 | 107906762 | + | ENSMUSG00000014782 | MGI:2142544 | MGI:2142544 | protein coding gene | Plekhg4 | pleckstrin homology domain containing, family G (with RhoGef domain) member 4 |
| 8 | 53.04 | 108227555 | 108231833 | - | ENSMUSG00000013155 | MGI:2142593 | MGI:2142593 | protein coding gene | E130303B06Rik | RIKEN cDNA E130303B06 gene |
| 8 | 53.1 | 108692775 | 108722604 | + | ENSMUSG00000031904 | MGI:2142598 | MGI:2142598 | protein coding gene | Slc7a6 | solute carrier family 7 (cationic amino acid transporter, y+ system), member 6 |
| 8 | 53.18 | 109206968 | 109375339 | + | ENSMUSG00000041949 | MGI:2142786 | MGI:2142786 | protein coding gene | Tmco7 | transmembrane and coiled-coil domains 7 |
| 8 | 53.55 | 109570189 | 109580589 | - | ENSMUSG00000031916 | MGI:2142885 | MGI:2142885 | protein coding gene | Cog8 | component of oligomeric golgi complex 8 |
| 8 | 53.1 | 108674299 | 108688613 | + | ENSMUSG00000031903 | MGI:2178076 | MGI:2178076 | protein coding gene | Pla2g15 | phospholipase A2, group XV |
| 8 | 53.04 | 107655700 | 107674009 | + | ENSMUSG00000060560 | MGI:2384581 | MGI:2384581 | protein coding gene | Ces4a | carboxylesterase 4A |
| 8 | 53.11 | 108734836 | 108776694 | + | ENSMUSG00000060098 | MGI:2384879 | MGI:2384879 | protein coding gene | Prmt7 | protein arginine N-methyltransferase 7 |
| 8 | 53.07 | 108508844 | 108520323 | - | ENSMUSG00000053687 | MGI:2442042 | MGI:2442042 | protein coding gene | Dpep2 | dipeptidase 2 |
| 8 | 53.04 | 107749045 | 107776953 | + | ENSMUSG00000031889 | MGI:2443049 | MGI:2443049 | protein coding gene | D230025D16Rik | RIKEN cDNA D230025D16 gene |
| 8 | 53.04 | 107836257 | 107850178 | - | ENSMUSG00000041679 | MGI:2443262 | MGI:2443262 | protein coding gene | Lrrc29 | leucine rich repeat containing 29 |
| 8 | 53.3 | 109407790 | 109417493 | - | ENSMUSG00000046691 | MGI:2443370 | MGI:2443370 | protein coding gene | Chtf8 | CTF8, chromosome transmission fidelity factor 8 homolog (S. cerevisiae) |
| 8 | 53.06 | 108417467 | 108418923 | + | ENSMUSG00000044287 | MGI:2443642 | MGI:2443642 | protein coding gene | Nrn1l | neuritin 1-like |
| 8 | 53.05 | 108368578 | 108375908 | - | ENSMUSG00000036672 | MGI:2443939 | MGI:2443939 | protein coding gene | Cenpt | centromere protein T |
| 8 | 53.05 | 108404781 | 108418808 | + | ENSMUSG00000036270 | MGI:2446249 | MGI:2446249 | protein coding gene | Edc4 | enhancer of mRNA decapping 4 |
| 8 | 53.04 | 107937471 | 107987984 | + | ENSMUSG00000054320 | MGI:2448585 | MGI:2448585 | protein coding gene | Lrrc36 | leucine rich repeat containing 36 |
| 8 | 53.04 | 107829501 | 107834523 | + | ENSMUSG00000014791 | MGI:2679007 | MGI:2679007 | protein coding gene | Elmo3 | engulfment and cell motility 3, ced-12 homolog (C. elegans) |
| 8 | 53.04 | 107853063 | 107871853 | - | ENSMUSG00000014778 | MGI:2679008 | MGI:2679008 | protein coding gene | Fhod1 | formin homology 2 domain containing 1 |
| 8 | 53.04 | 108214806 | 108222065 | + | ENSMUSG00000050357 | MGI:2685431 | MGI:2685431 | protein coding gene | Rltpr | RGD motif, leucine rich repeats, tropomodulin domain and proline-rich containing |
| 8 | 53.04 | 107872158 | 107893781 | + | ENSMUSG00000014786 | MGI:2685542 | MGI:2685542 | protein coding gene | Slc9a5 | solute carrier family 9 (sodium/hydrogen exchanger), member 5 |
| 8 | 53.04 | 107813824 | 107819998 | - | ENSMUSG00000043251 | MGI:3041195 | MGI:3041195 | protein coding gene | Exoc3l | exocyst complex component 3-like |
| 8 | 53.04 | 107906707 | 107937402 | - | ENSMUSG00000051648 | MGI:3045294 | MGI:3045294 | protein coding gene | Kctd19 | potassium channel tetramerisation domain containing 19 |
| 8 | 53.16 | 109024422 | 109031242 | + | ENSMUSG00000054069 | MGI:3045356 | MGI:3045356 | protein coding gene | 6030452D12Rik | RIKEN cDNA 6030452D12 gene |
| 8 | 53.06 | 108424341 | 108455678 | + | ENSMUSG00000048310 | MGI:3528383 | MGI:3528383 | protein coding gene | Pskh1 | protein serine kinase H1 |
| 8 | 53.04 | 107607653 | 107617829 | + | ENSMUSG00000062181 | MGI:3644960 | MGI:3644960 | protein coding gene | Ces3b | carboxylesterase 3B |
| 8 | 53.61 | 109608407 | 109609288 | - |  |  | MGI:3647574 | protein coding gene | Gm8842 | predicted gene 8842 |
| 8 | 53.04 | 108109219 | 108110100 | - | ENSMUSG00000091957 | MGI:3647575 | MGI:3647575 | protein coding gene | Gm8841 | predicted gene 8841 |
| 8 | 53.04 | 107538350 | 107544310 | + | ENSMUSG00000091813 | MGI:3648740 | MGI:3648740 | protein coding gene | Ces2h | carboxylesterase 2H |
| 8 | 53.04 | 108097554 | 108098197 | + | ENSMUSG00000038000 | MGI:87873 | MGI:4937014 | protein coding gene | Gm17380 | predicted gene, 17380 |
| 8 | 53.04 | 108222059 | 108224995 | - | ENSMUSG00000031885 | MGI:99851 | MGI:87873 | protein coding gene | Acd | adrenocortical dysplasia |
| 8 | 53.18 | 109127251 | 109194146 | + |  |  | MGI:88354 | protein coding gene | Cdh1 | cadherin 1 |
| 8 | 53.16 | 109034791 | 109080808 | + |  |  | MGI:88356 | protein coding gene | Cdh3 | cadherin 3 |
| 8 | 53.06 | 108455894 | 108457762 | - |  |  | MGI:88558 | protein coding gene | Ctrl | chymotrypsin-like |
| 8 | 53.04 | 108090600 | 108092198 | - |  |  | MGI:892013 | protein coding gene | Agrp | agouti related protein |
| 8 | 53.06 | 108463451 | 108467282 | - |  |  | MGI:96755 | protein coding gene | Lcat | lecithin cholesterol acyltransferase |
| 8 | 53.04 | 107694574 | 107741886 | + |  |  | MGI:99851 | protein coding gene | Cbfb | core binding factor beta |
| 8 | 53.04 | 107776538 | 107779053 | - | ENSMUSG00000069920 | MGI:2142841 | MGI:2142841 | pseudogene | B3gnt9-ps | UDP-GlcNAc:betaGal beta-1,3-N-acetylglucosaminyltransferase 9, pseudogene |
| 8 | 53.17 | 109096906 | 109097250 | - | ENSMUSG00000060019 | MGI:3641908 | MGI:3641908 | pseudogene | Gm10073 | predicted pseudogene 10073 |
| 8 | 53.89 | 109786646 | 109787169 | - |  |  | MGI:3642474 | pseudogene | Rps18-ps3 | ribosomal protein S18, pseudogene 3 |
| 8 | 53.04 | 108184234 | 108184687 | - | ENSMUSG00000080021 | MGI:3642938 | MGI:3642938 | pseudogene | Gm5915 | predicted pseudogene 5915 |
| 8 | 54.55 | 110214308 | 110214744 | - |  |  | MGI:3643403 | pseudogene | Gm8940 | predicted gene 8940 |
| 8 | 53.04 | 107634522 | 107635321 | + |  |  | MGI:3643513 | pseudogene | Gm8804 | predicted gene 8804 |
| 8 | 53.04 | 108027099 | 108027973 | + |  |  | MGI:3643717 | pseudogene | Gm8838 | predicted gene 8838 |
| 8 | 53.37 | 109451669 | 109452313 | - | ENSMUSG00000074108 | MGI:3648679 | MGI:3648679 | pseudogene | Rpl10-ps2 | ribosomal protein L10, pseudogene 2 |
| 8 | 54.16 | 109963051 | 109963463 | - | ENSMUSG00000059775 | MGI:3704322 | MGI:3704322 | pseudogene | Rps26-ps1 | ribosomal protein S26, pseudogene 1 |
| 8 | 53.54 | 109561380 | 109562006 | - | ENSMUSG00000085464 | MGI:3802096 | MGI:3802095 | pseudogene | Gm16209 | predicted gene 16209 |
| 8 | 53.04 | 107724593 | 107724964 | + | ENSMUSG00000000303 | MGI:88354 | MGI:5012333 | pseudogene | Gm20148 | predicted gene, 20148 |
| 8 | 53.14 | 108929519 | 108930429 | - |  |  | MGI:1915413 | unclassified gene | A930006D01Rik | RIKEN cDNA A930006D01 gene |
| 8 | 53.12 | 108807207 | 108823917 | + | ENSMUSG00000074113 | MGI:3642307 | MGI:3642307 | unclassified gene | Gm10629 | predicted gene 10629 |
| 8 | 53.04 | 108148236 | 108161778 | - | ENSMUSG00000087436 | MGI:3801720 | MGI:3779534 | unclassified gene | Gm5914 | predicted gene 5914 |
| 8 | 53.06 | 108458233 | 108469743 | + | ENSMUSG00000083734 | MGI:3802095 | MGI:3801720 | unclassified gene | Gm16156 | predicted gene 16156 |
| 8 | 53.53 | 109553575 | 109555088 | - | ENSMUSG00000089432 | MGI:3837211 | MGI:3802096 | unclassified gene | Gm16208 | predicted gene 16208 |
| 8 | 53.11 | 108761803 | 108949628 | - | ENSMUSG00000090526 | MGI:4937014 | MGI:4936879 | unclassified gene | Gm17245 | predicted gene, 17245 |
| 8 | 53.04 | 107813889 | 107816007 | + | ENSMUSG00000061048 | MGI:88356 | MGI:5012348 | unclassified gene | Gm20163 | predicted gene, 20163 |
| 8 | 53.05 | 108400410 | 108403236 | - | ENSMUSG00000031896 | MGI:88558 | MGI:5012380 | unclassified gene | Gm20195 | predicted gene, 20195 |
| 8 | 53.15 | 108951850 | 108968160 | + | ENSMUSG00000005705 | MGI:892013 | MGI:5012415 | unclassified gene | Gm20230 | predicted gene, 20230 |
| 8 | 53.18 | 109192784 | 109221853 | - | ENSMUSG00000035237 | MGI:96755 | MGI:5012439 | unclassified gene | Gm20254 | predicted gene, 20254 |
|  |  |  |  |  |  |  |  |  |  |  |
