# Supplementary material for: High-precision genetic mapping of behavioral traits in the diversity outbred mouse population
Source: Genes Brain Behav. 2013 Mar 20;12(4):424–37. doi: 10.1111/gbb.12029 (PMC3709837; doi:10.1111/gbb.12029)
Supplement: Supplementary file 9 [file gbb0012-0424-SD9.doc]

**Supplemental table 9:** Genes within QTL interval on chromosome 14 for bottom distance traveled.

| Chr | cM | start | end | strand NCBI Build 37 | MGI ID | Feature Type | Symbol | Name |
| --- | --- | --- | --- | --- | --- | --- | --- | --- |
| 14 | 11.91 | 22650491 | 22665101 | + | MGI:106915 | protein coding gene | Vdac2 | voltage-dependent anion channel 2 |
| 14 | 11.85 | 22552617 | 22570403 | - | MGI:1351599 | protein coding gene | Dusp13 | dual specificity phosphatase 13 |
| 14 | 12 | 22803184 | 22808823 | - | MGI:1353644 | protein coding gene | Zfp503 | zinc finger protein 503 |
| 14 | 11.73 | 22319076 | 22491354 | + | MGI:1858746 | protein coding gene | Myst4 | MYST histone acetyltransferase monocytic leukemia 4 |
| 14 | 11.86 | 22569753 | 22617947 | + | MGI:1914880 | protein coding gene | Samd8 | sterile alpha motif domain containing 8 |
| 14 | 11.92 | 22665083 | 22668199 | - | MGI:1916406 | protein coding gene | Comtd1 | catechol-O-methyltransferase domain containing 1 |
| 14 | 12.03 | 22838934 | 23875307 | + | MGI:1923883 | protein coding gene | 1700112E06Rik | RIKEN cDNA 1700112E06 gene |
| 14 | 11.58 | 21850664 | 21871730 | - | MGI:1929212 | protein coding gene | Ap3m1 | adaptor-related protein complex 3, mu 1 subunit |
| 14 | 11.82 | 22495804 | 22533798 | - | MGI:3647127 | protein coding gene | Dupd1 | dual specificity phosphatase and pro isomerase domain containing 1 |
| 14 | 11.58 | 21871855 | 22267769 | + | MGI:87930 | protein coding gene | Adk | adenosine kinase |
| 14 | 11.53 | 21554097 | 21613310 | - | MGI:88259 | protein coding gene | Camk2g | calcium/calmodulin-dependent protein kinase II gamma |
| 14 | 11.53 | 21655884 | 21662610 | + | MGI:97611 | protein coding gene | Plau | plasminogen activator, urokinase |
| 14 | 11.53 | 21748655 | 21852895 | + | MGI:98927 | protein coding gene | Vcl | vinculin |
| 14 | 11.53 | 21675233 | 21675643 | - | MGI:3645269 | pseudogene | Gm6128 | predicted pseudogene 6128 |
| 14 | 11.92 | 22666800 | 22676148 | + | MGI:2442125 | unclassified gene | A430057M04Rik | RIKEN cDNA A430057M04 gene |
| 14 | 11.86 | 22560765 | 22565854 | + | MGI:3801894 | unclassified gene | Gm15935 | predicted gene 15935 |
|  |  |  |  |  |  |  |  |  |
