# Supplementary material for: High-precision genetic mapping of behavioral traits in the diversity outbred mouse population
Source: Genes Brain Behav. 2013 Mar 20;12(4):424–37. doi: 10.1111/gbb.12029 (PMC3709837; doi:10.1111/gbb.12029)
Supplement: Supplementary file 10 [file gbb0012-0424-SD10.doc]

**Supplemental table 10: Genes within QTL interval on chromosome 6 for climbing.**

| Chr | cM | start | end | strand NCBI Build 37 | MGI ID | Feature Type | Symbol | Name |  |  |
| --- | --- | --- | --- | --- | --- | --- | --- | --- | --- | --- |
| 6 | 45.74 | 98875332 | 99216526 | - | MGI:1914004 | protein coding gene | Foxp1 | forkhead box P1 | ENSMUSG00000035158 | MGI:104554 |
| 6 | 45.67 | 98188008 | 98292748 | - | MGI:2685611 | protein coding gene | Gm765 | predicted gene 765 | ENSMUSG00000030067 | MGI:1914004 |
| 6 | 45.05 | 97757052 | 97971343 | + | MGI:104554 | protein coding gene | Mitf | microphthalmia-associated transcription factor | ENSMUSG00000090667 | MGI:2685611 |
| 6 | 45.1 | 97840282 | 97840533 | - | MGI:3782979 | pseudogene | Gm15531 | predicted gene 15531 | ENSMUSG00000083086 | MGI:3782979 |
| 6 | 45.71 | 98225530 | 98226107 | + | MGI:3645233 | pseudogene | Gm7892 | predicted gene 7892 |  |  |
|  |  |  |  |  |  |  |  |  |  |  |
