# Supplementary material for: High-precision genetic mapping of behavioral traits in the diversity outbred mouse population
Source: Genes Brain Behav. 2013 Mar 20;12(4):424–37. doi: 10.1111/gbb.12029 (PMC3709837; doi:10.1111/gbb.12029)
Supplement: Supplementary file 11 [file gbb0012-0424-SD11.doc]

**S11**

**Correlations among activity measures and traits with significant QTLs**

|  | **OF total distance (cm) traveled** | **OF time in center slope** | **OF % time immobile** | **LD distance (cm) traveled in light** | **LD % time in light slope** | **LD % time in light** | **VC total distance (cm) traveled** | **VC distance (cm) traveled in bottom (ratio)** | **TST climbing frequency** |
| --- | --- | --- | --- | --- | --- | --- | --- | --- | --- |
| **OF total distance (cm) traveled** | 1.0000 |  |  |  |  |  |  |  |  |
| **OF time in center slope** | 0.2663 | 1.0000 |  |  |  |  |  |  |  |
| **OF % time immobile** | -0.7758 | -0.1949 | 1.0000 |  |  |  |  |  |  |
| **LD distance (cm) traveled in light** | 0.5941 | 0.1960 | -0.4915 | 1.0000 |  |  |  |  |  |
| **LD % time in light slope** | 0.1159 | 0.0492 | -0.1818 | 0.2293 | 1.0000 |  |  |  |  |
| **LD % time in light** | 0.3290 | 0.1419 | -0.2910 | 0.7947 | 0.2157 | 1.0000 |  |  |  |
| **VC total distance (cm) traveled** | 0.6497 | 0.1778 | -0.6113 | 0.6610 | 0.1721 | 0.4168 | 1.0000 |  |  |
| **VC distance (cm) traveled in bottom (ratio)** | 0.1994 | -0.0119 | -0.2371 | 0.3712 | 0.1260 | 0.2557 | 0.5561 | 1.0000 |  |
| **TST climbing frequency** | 0.3172 | 0.0556 | -0.3592 | 0.0586 | 0.0274 | 0.0223 | 0.1497 | 0.1477 | 1.0000 |
