# Supplementary material for: High-precision genetic mapping of behavioral traits in the diversity outbred mouse population
Source: Genes Brain Behav. 2013 Mar 20;12(4):424–37. doi: 10.1111/gbb.12029 (PMC3709837; doi:10.1111/gbb.12029)
Supplement: Supplementary file 12 [file gbb0012-0424-SD12.doc]

**Table S12**

**Principal Components Analysis**

Eigenvalues

| **Number** | **Eigenvalue** | **Percent** | **Percent** | **Cum Percent** | **ChiSquare** | **DF** | **Prob>ChiSq** |
| --- | --- | --- | --- | --- | --- | --- | --- |
| 1 | 4.1744 | 27.830 | 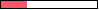 | 27.830 | 1374.11 | 119.000 | <.0001* |
| 2 | 1.5504 | 10.336 | 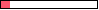 | 38.166 | 781.403 | 104.000 | <.0001* |
| 3 | 1.2528 | 8.352 | 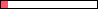 | 46.518 | 685.752 | 90.000 | <.0001* |
| 4 | 1.1613 | 7.742 | 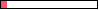 | 54.260 | 626.293 | 77.000 | <.0001* |
| 5 | 1.1202 | 7.468 | 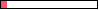 | 61.728 | 568.767 | 65.000 | <.0001* |
| 6 | 1.0116 | 6.744 | 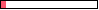 | 68.472 | 502.184 | 54.000 | <.0001* |
| 7 | 0.8589 | 5.726 | 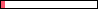 | 74.197 | 439.447 | 44.000 | <.0001* |
| 8 | 0.8270 | 5.513 | 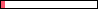 | 79.711 | 393.111 | 35.000 | <.0001* |
| 9 | 0.7733 | 5.155 | 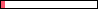 | 84.866 | 335.279 | 27.000 | <.0001* |
| 10 | 0.6585 | 4.390 | 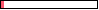 | 89.256 | 264.808 | 20.000 | <.0001* |
| 11 | 0.5648 | 3.765 | 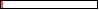 | 93.021 | 197.323 | 14.000 | <.0001* |
| 12 | 0.4207 | 2.805 | 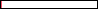 | 95.826 | 122.933 | 9.000 | <.0001* |
| 13 | 0.3250 | 2.167 | 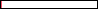 | 97.993 | 67.180 | 5.000 | <.0001* |
| 14 | 0.1787 | 1.192 | 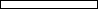 | 99.184 | 9.751 | 2.000 | 0.0076* |
| 15 | 0.1223 | 0.816 | 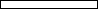 | 100.000 | 0.000 | 0.000 | . |

Loading Matrix

|  | **Prin1** | **Prin2** | **Prin3** | **Prin4** | **Prin5** | **Prin6** |
| --- | --- | --- | --- | --- | --- | --- |
| OF distance (cm) traveled first 4 min | 0.77051 | -0.33276 | -0.13266 | 0.07754 | -0.14276 | 0.09826 |
| OF total distance (cm) traveled | 0.81338 | -0.42481 | 0.02882 | 0.16204 | -0.00399 | 0.00757 |
| OF % time in center | -0.01601 | 0.02559 | 0.09732 | 0.34516 | 0.47983 | 0.63155 |
| OF % time in center slope | 0.23363 | -0.25501 | 0.46162 | 0.38435 | -0.08224 | -0.09878 |
| OF fecal boli count | -0.14643 | 0.21834 | 0.52520 | 0.14559 | 0.31817 | 0.20724 |
| OF % time immobile | -0.77176 | 0.36727 | 0.00435 | -0.04773 | -0.09263 | 0.07192 |
| VC total transitions between top and bottom | 0.73559 | 0.29692 | -0.10300 | 0.14700 | -0.08579 | 0.02769 |
| VC time (s) in bottom first 4 min | 0.63963 | 0.52321 | -0.29153 | -0.01558 | -0.07410 | 0.19413 |
| VC time (s) in bottom slope | 0.16760 | 0.35309 | 0.34209 | 0.41215 | 0.09202 | -0.56623 |
| VC % time in bottom | 0.63067 | 0.65389 | -0.12308 | 0.10704 | -0.01075 | -0.07100 |
| LDBOX number of light-dark transitions | 0.60085 | -0.10290 | 0.21537 | -0.36590 | -0.03426 | 0.09588 |
| LDBOX % time in light | 0.52675 | 0.12527 | 0.34959 | -0.22373 | -0.14117 | 0.24780 |
| LDBOX % time in light slope | 0.26385 | 0.06548 | 0.51003 | -0.51504 | 0.00326 | -0.11783 |
| TST climbing frequency | 0.36297 | -0.23765 | -0.26762 | 0.06365 | 0.54266 | -0.26268 |
| TST immobility frequency | -0.17709 | -0.09891 | 0.08869 | 0.42690 | -0.64495 | 0.16602 |

|  | **Prin7** | **Prin8** | **Prin9** | **Prin10** | **Prin11** | **Prin12** | **Prin13** | **Prin14** | **Prin15** |
| --- | --- | --- | --- | --- | --- | --- | --- | --- | --- |
| OF Distance (cm) traveled first 4 min | -0.01673 | 0.15064 | -0.20540 | -0.16064 | -0.01635 | -0.05631 | 0.33996 | 0.05205 | -0.17104 |
| OF Total distance (cm) traveled | -0.02957 | 0.11216 | -0.16541 | -0.05827 | -0.05631 | -0.03581 | 0.06007 | -0.08000 | 0.26853 |
| OF % time in center | 0.46234 | -0.00106 | 0.11747 | -0.11342 | -0.02460 | -0.00469 | -0.00219 | -0.01561 | -0.00983 |
| OF % time in center slope | -0.23217 | -0.56227 | 0.31556 | -0.16572 | 0.06518 | -0.01346 | 0.02774 | -0.00423 | -0.02723 |
| OF Fecal boli count | -0.56773 | 0.32755 | -0.20889 | 0.05888 | 0.11000 | 0.01055 | 0.00847 | 0.02317 | -0.01240 |
| OF % time immobile | 0.02507 | -0.13759 | 0.06012 | 0.05413 | 0.10031 | 0.16913 | 0.42052 | -0.03873 | 0.10169 |
| VC total transitions between top and bottom | -0.04898 | -0.01499 | -0.05115 | -0.08136 | -0.08715 | 0.54941 | -0.09054 | -0.01847 | -0.02363 |
| VC time (s) in bottom first 4 min | -0.15962 | -0.04810 | 0.22281 | -0.08459 | 0.06862 | -0.15170 | -0.00066 | 0.26405 | 0.08170 |
| VC time (s) in bottom slope | 0.39907 | 0.08470 | -0.20289 | 0.06926 | 0.02487 | -0.03796 | 0.04282 | 0.12220 | 0.00651 |
| VC % time in bottom | -0.04866 | 0.00914 | 0.07242 | -0.03026 | 0.05284 | -0.21846 | 0.01617 | -0.28611 | -0.04092 |
| LDBOX number of light-dark transitions | 0.20462 | -0.06744 | -0.04550 | 0.15730 | 0.59953 | 0.06153 | -0.03484 | -0.00844 | -0.00545 |
| LDBOX % time in light | 0.05764 | -0.24443 | -0.10095 | 0.50158 | -0.35187 | -0.05192 | 0.03868 | 0.01463 | -0.01351 |
| LDBOX % time in light slope | 0.12905 | 0.31275 | 0.38921 | -0.29487 | -0.17258 | 0.03325 | 0.05389 | -0.00591 | 0.00901 |
| TST climbing frequency | -0.10948 | 0.12568 | 0.42465 | 0.38417 | 0.00794 | 0.07544 | 0.10395 | 0.00176 | -0.01907 |
| TST immobility frequency | 0.06145 | 0.40208 | 0.31679 | 0.22848 | 0.07457 | 0.00614 | -0.03792 | -0.01208 | -0.00575 |
