# Supplementary figures and images for: High-precision genetic mapping of behavioral traits in the diversity outbred mouse population
Source: Genes Brain Behav. 2013 Mar 20;12(4):424–37. doi: 10.1111/gbb.12029 (PMC3709837; doi:10.1111/gbb.12029)

**Figure S1**

**QTL mapping principal components 1-6**

**
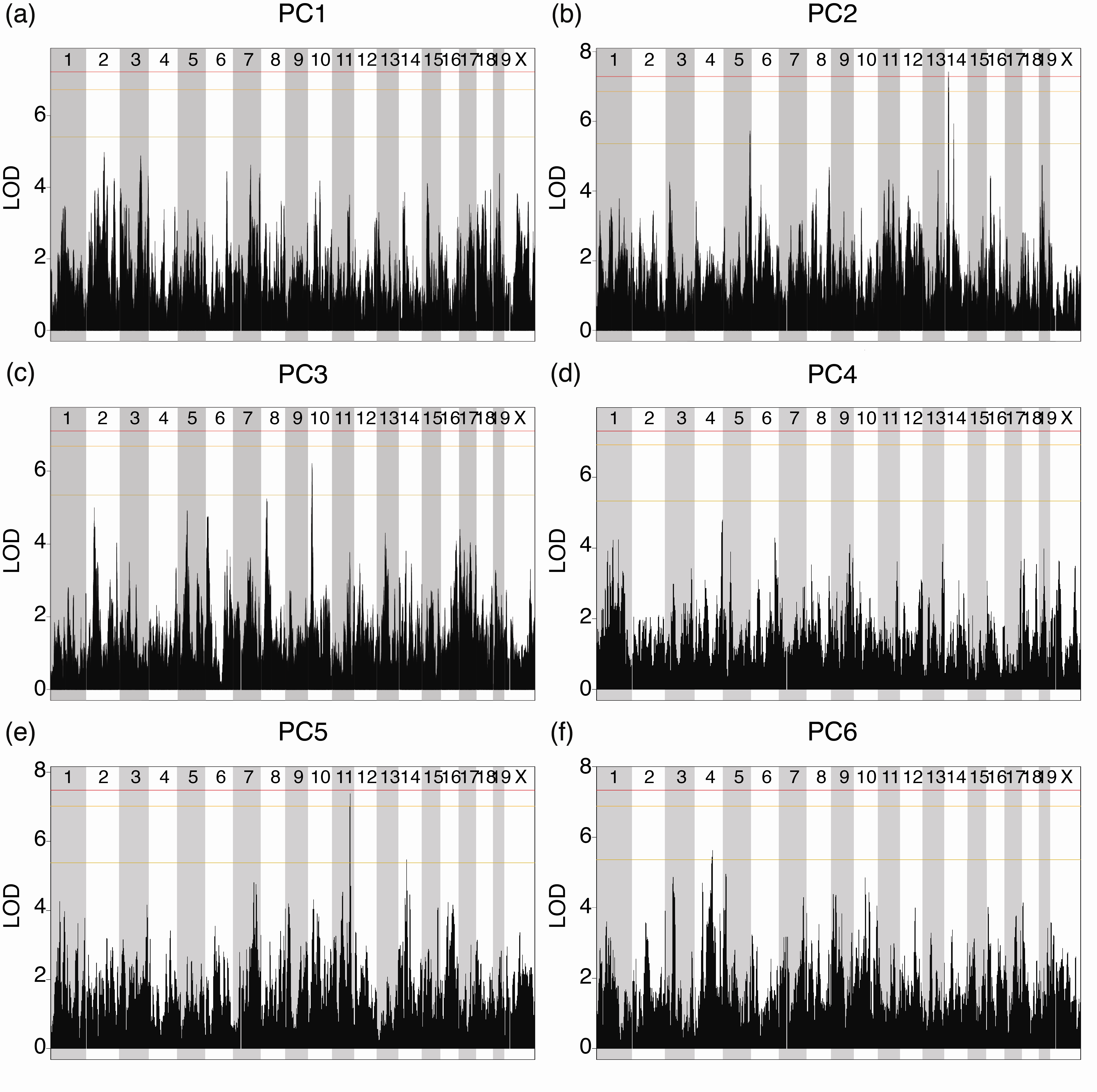
**

**QTL mapping conditioned on locomotor activity**

Supplement: Supplementary file 13 [file gbb0012-0424-SD13.doc]

**Figure S2 Center time slope in open-field**

**
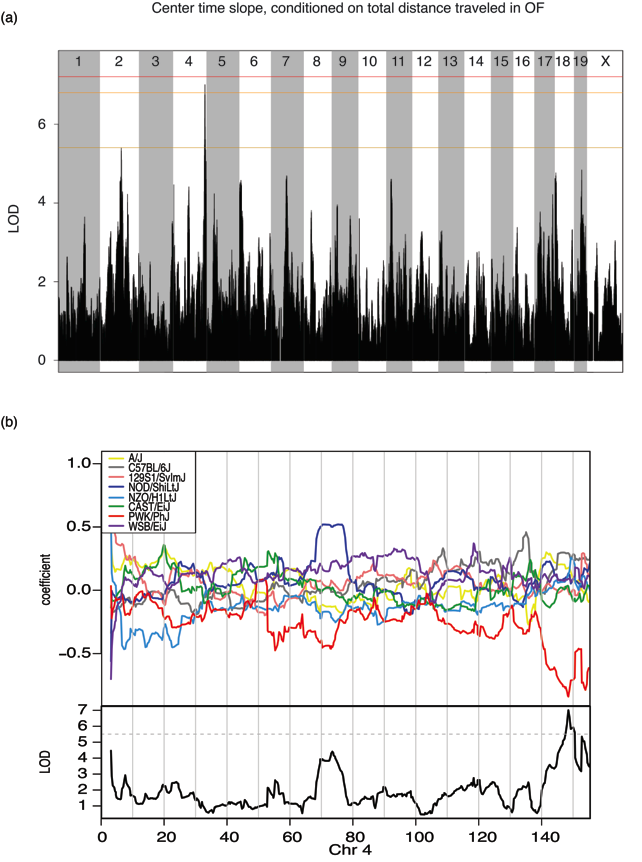
**

Supplement: Supplementary file 14 [file gbb0012-0424-SD14.doc]

**Figure S3 Immobility in the open-field**

**
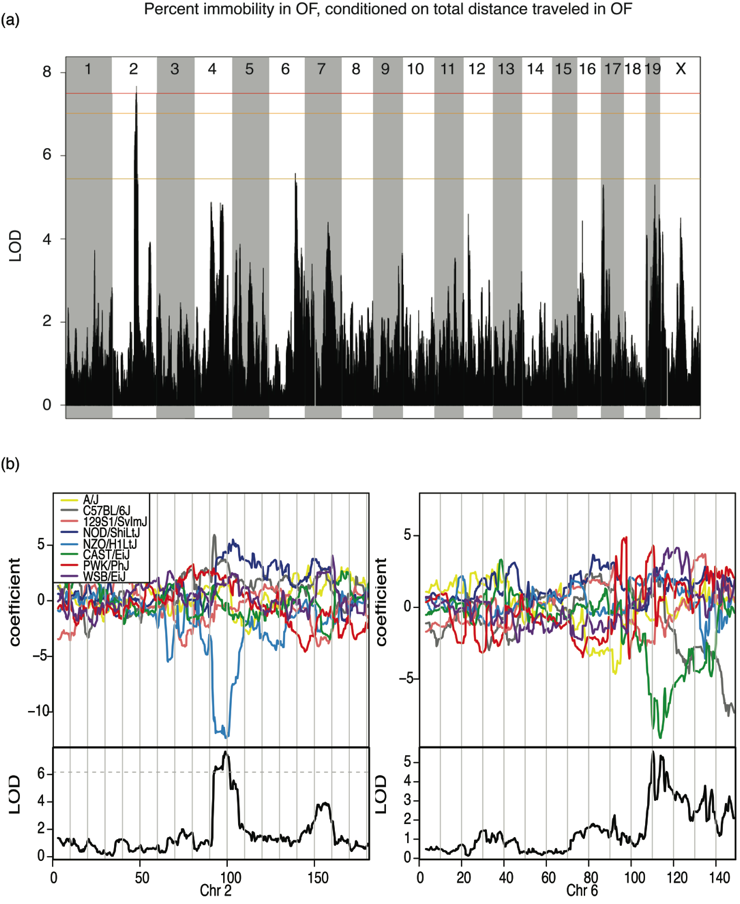
**

Supplement: Supplementary file 15 [file gbb0012-0424-SD15.doc]

**Figure S4 Climbing frequency during tail-suspension test**

**
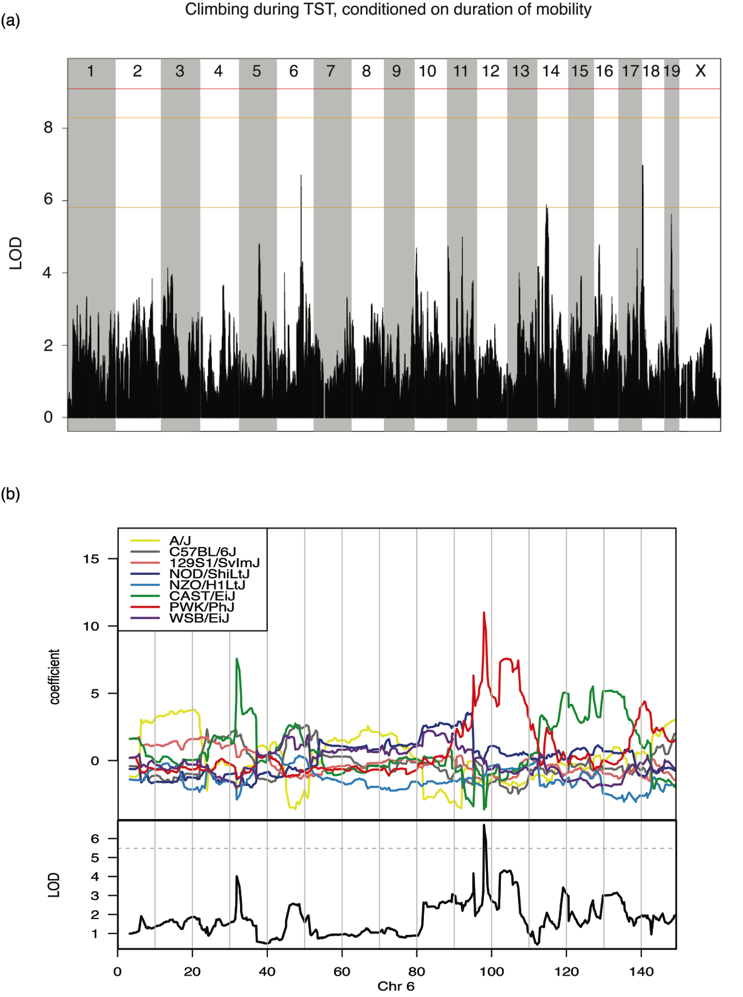
**

Supplement: Supplementary file 16 [file gbb0012-0424-SD16.doc]

**Figure S5 Percent time in light in the light-dark box**

**
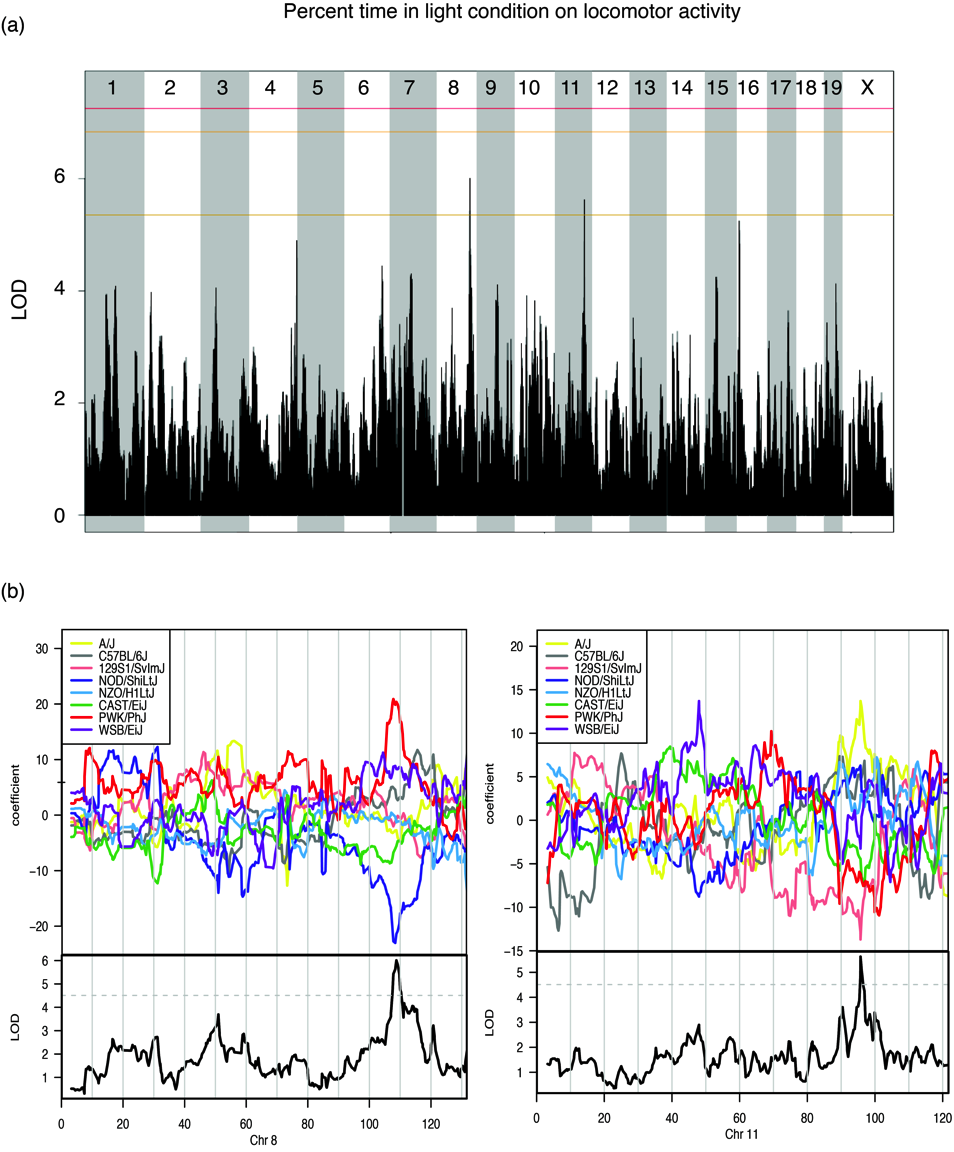
**

Supplement: Supplementary file 17 [file gbb0012-0424-SD17.doc]

**Figure S6 Time spent in the bottom area of the visual cliff (ratio)**

**
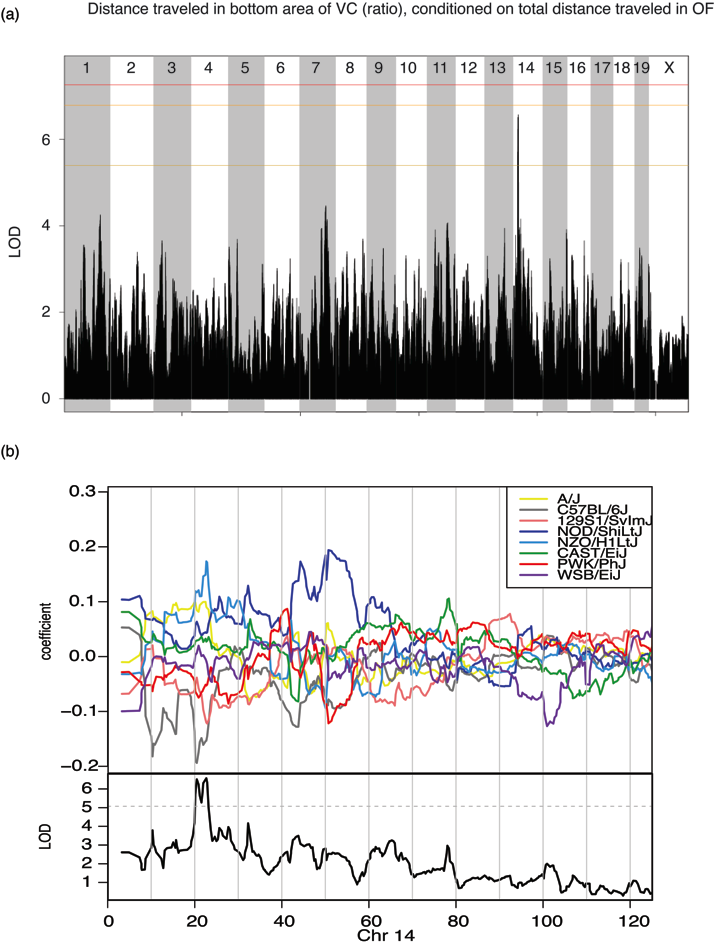
**

Supplement: Supplementary file 18 [file gbb0012-0424-SD18.doc]
