# Supplementary material for: High-precision genetic mapping of behavioral traits in the diversity outbred mouse population
Source: Genes Brain Behav. 2013 Mar 20;12(4):424–37. doi: 10.1111/gbb.12029 (PMC3709837; doi:10.1111/gbb.12029)
Supplement: Supplementary file 19 [file gbb0012-0424-SD19.doc]

**Figure S7**

**Phenotypic values for QTL associated with wild-derived alleles vs. locomotor activity**.

**Wild-derived QTL allele—green (CAST) or red (PWK).**


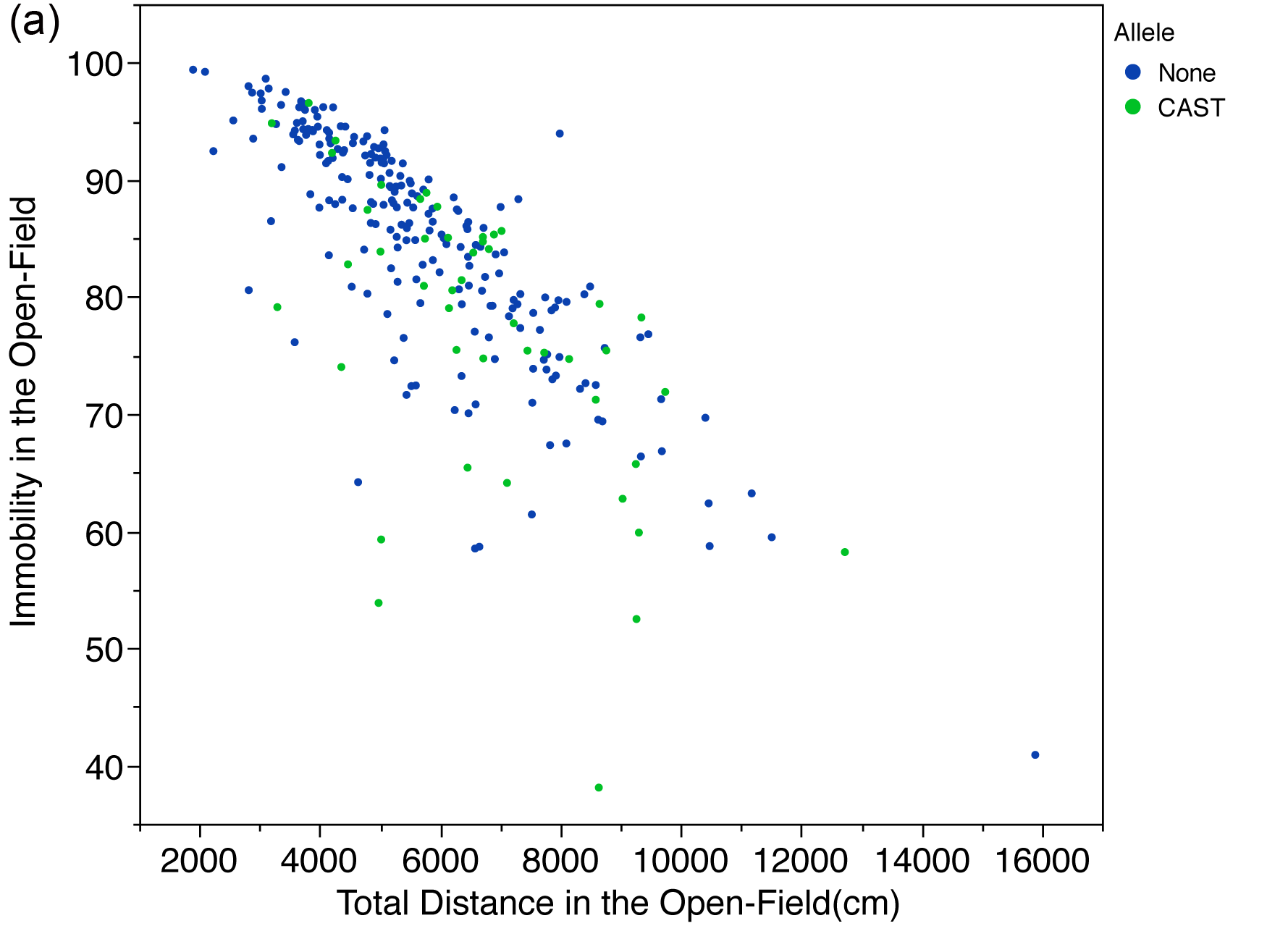


**
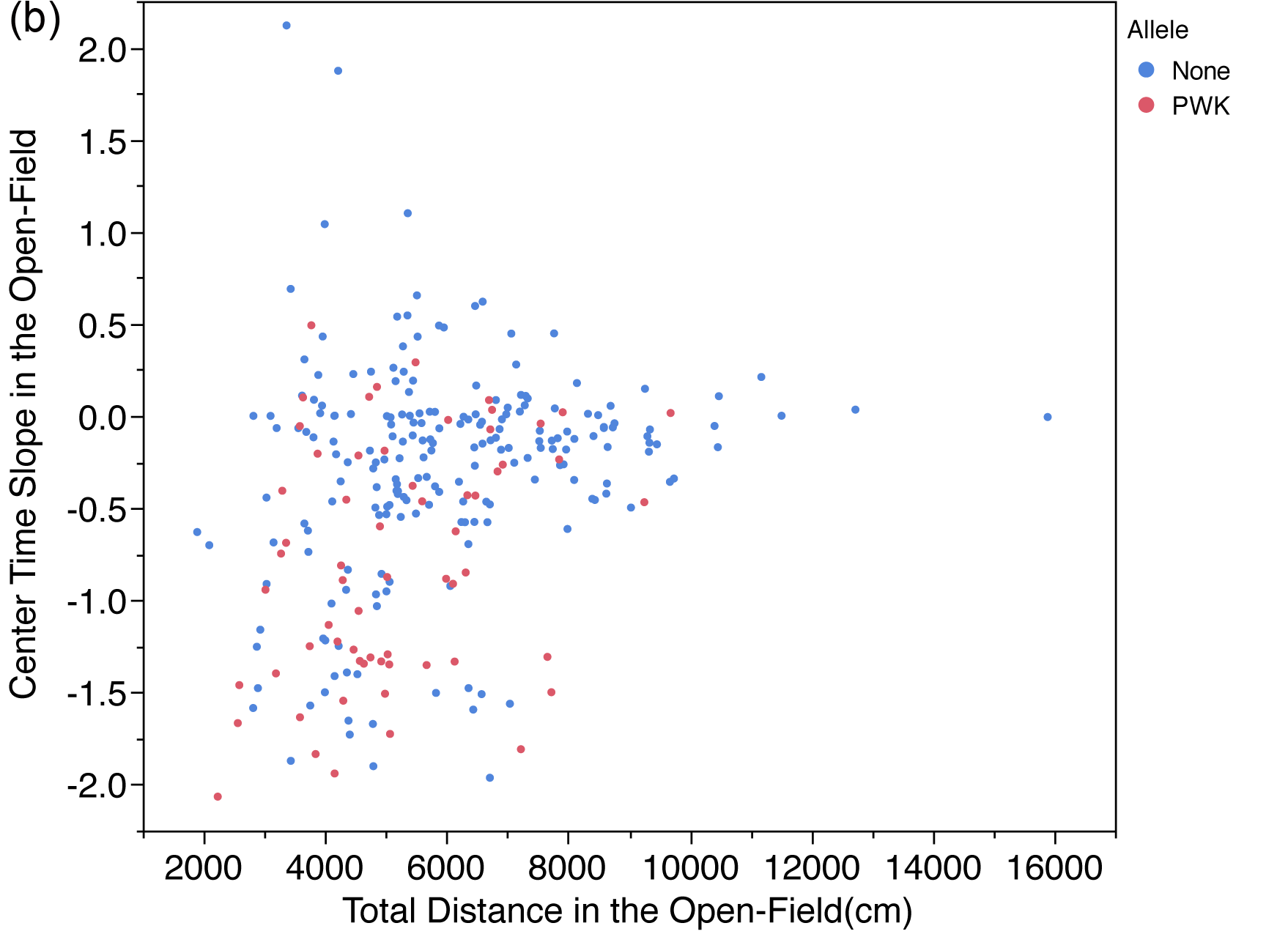
**

**
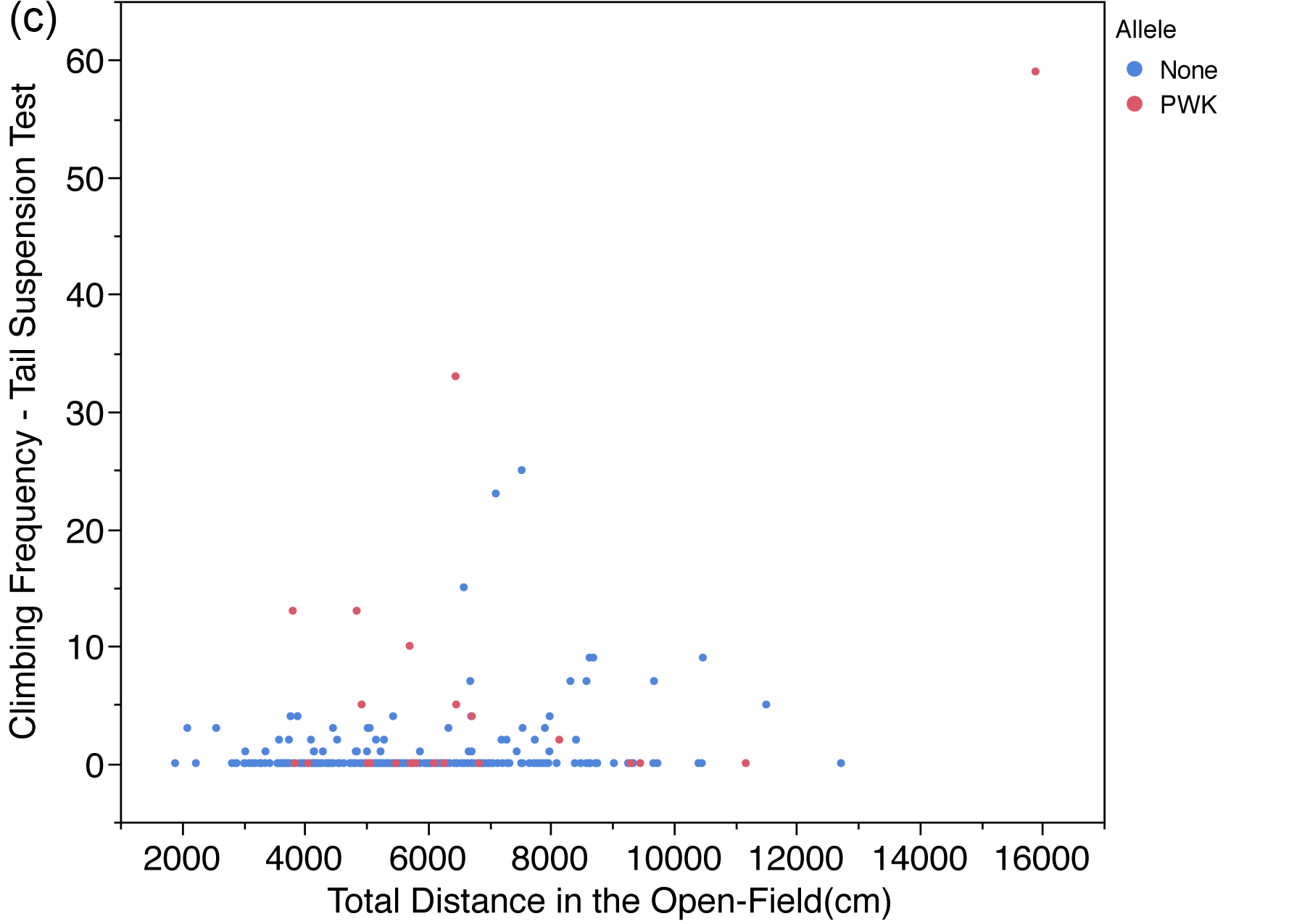
**
